# Policies and strategies on active and healthy ageing: a scoping review of the recommendations of European and international agencies

**DOI:** 10.3389/fpubh.2025.1712417

**Published:** 2026-01-07

**Authors:** Silvia Caristia, Erica Viola, Thellenxa Kalemi, Davide Servetti, Samuele Poy, Fabrizio Faggiano

**Affiliations:** 1 Department of Sustainable Development and Ecological Transition, University of Piemonte Orientale, Vercelli, Italy; 2 School of Medicine, University of Piemonte Orientale, Vercelli, Italy; 3 Department of Law and Political, Economic and Social Sciences, University of Piemonte Orientale, Alessandria, Italy

**Keywords:** ageing, health promotion, active and healthy ageing, health in all policies, international and European organizations, equity, sustainability, age-friendly policies

## Abstract

**Background:**

Over the past six decades, life expectancy has significantly increased, with a concurrent decline in fertility rates, leading to unprecedented demographic shifts. These changes have deeply changed population structures and presented challenges for welfare systems, particularly regarding sustainability and intergenerational equity.

**Aim:**

This study aimed to review recommendations from major international organizations [e.g., the World Health Organisation (WHO), Organisation for Economic Co-operation and Development (OECD), European Union (EU)] to promote active and healthy ageing (AHA).

**Methods:**

A scoping review was conducted to identify policy-relevant documents published between 2008 and 2023 by major international and European organizations. The search strategy, carried out via Google using targeted search strings and snowballing methods, identified 33 reports to be included. Inclusion criteria required that documents be targeted to policymakers and present recommendations for promoting AHA. Documents focused solely on treatment, frailty, or child/youth interventions were excluded. Data extraction was carried out independently by two reviewers.

**Results:**

The elaboration of the 33 included policy-oriented reports published between 2008 and 2023 yielded 554 actions. These were classified across 19 policy sectors and grouped into 14 cross-sectoral strategies to support healthy ageing. The most represented sectors were health (37.5% of actions), labor, social welfare, and civil rights. Strategies included enhancing access to quality services, reducing non-communicable diseases, supporting prolonged working lives, enabling ageing in place, tackling socio-economic divides, and fostering better laws. Specific interventions ranged from tax incentives for a healthy diet to flexible retirement policies, caregiver support, and urban planning for inclusive environments.

**Conclusion:**

There is a need for a multi-sectoral approach to policymaking, as healthy ageing promotion cannot rely solely on health policy. Focusing on preventive rather than disease-oriented actions, AHA policies must be grounded in equity, sustainability, and long-term planning. Despite limited rigorous evidence—often based on expert consensus—the study offers a practical classification system to guide national and local policy development. It provides a comprehensive framework to help governments shift from reactive healthcare to proactive, integrated approaches that promote lifelong wellbeing and support the sustainability of welfare systems amid demographic and epidemiological transitions.

## 1 Introduction

An average European born in 1960 could expect to live for 69 years; a European born in 2023 could expect to live for 81 years [data for European Union (EU) countries] (1). The same data at a world level was comparable, even at a lower level: an average world citizen could expect to live 51 years if born in 1960 and 73 years if born in 2023 (1). The gain in life expectancy of 22 years in less than 62 years is extraordinary: it has never happened in human history!

This increase can be attributed to several factors. Diseases that were once considered fatal are now manageable due to significant advancements in medical treatments (2). Notably, improvements in the management of major causes of death, such as cancer and cardiovascular diseases, have substantially enhanced survival rates. Furthermore, there has been a dramatic decline in infant mortality, driven by improved vaccine coverage, better treatment of infectious diseases, and, crucially, enhancements in living conditions, housing, and nutrition (3). Additionally, exposure to key health risk factors, including tobacco smoking, alcohol abuse, and air pollution, has decreased markedly (4).

A decline in fertility rates has paralleled the mortality trends. In Europe, the average woman in 2023 was having 1.4 children, compared to 2.6 children in 1960, and the same data at a world level were 4.7 and 2.2, respectively (1). Italy ranks among the European countries with the lowest average number of children per woman, second only to Spain (1.2 in 2023) (1).

The result is an increase in survival and a decline in population replacement, the Demographic Winter (5). These two processes are leading to a significant distortion of the population structure, which has been shaped like a pyramid. In the second part of the 20th century, the structure of the population in rich societies began to appear like a barrel, with a big bulge in the middle (the Baby Boom generation), older people, and fewer children.

However, population ageing can be seen as a threat to societies, governments, and economies. The costs of health care, long-term care, and pensions are expected to rise disproportionately, and public revenues may collapse due to a shortage of productive workers caused by large retirements of the older population and the lack of replacement due to the fall in fertility.

Nevertheless, there is vast research arguing that population ageing need not be a threat to the sustainability of states or their health care systems (6). This requires the extensive adoption of policies able to contrast the negative effects of this demographic transition, allowing to avoid a political divide between different generations. This can be done by adopting a life-course perspective that maximizes the health and wellbeing of the whole population.

It is a significant challenge for policymakers, demanding innovative solutions across multiple domains. Several approaches, though not alternatives, can be recognized in the literature: one approach involves reorganizing health services to better address the needs of aging populations. In this context, the literature emphasizes the need to rethink care models by ensuring multi-actor governance systems and integrated services, while reshaping social policies and the roles of various actors in policy design and implementation (7, 8). The COVID-19 pandemic has further underscored the urgency for innovative, sustainable solutions in health system organization within the social investment paradigm (9, 10), reinforcing the critical role of local governments in responding to the growing demand for community-centered services. In response, many local governments are increasingly adopting cooperative public-private governance models, which streamline administrative processes and build trust within communities (11), especially as the national healthcare system continues to lose credibility.

Another essential strategy to address population aging is fostering intergenerational relationships through specific programs. In countries with weak welfare systems, informal intergenerational transfers are essential (12, 13). This aligns with the concept of intergenerational solidarity, which emphasizes mutual support across generations (14, 15). As family structures change, the importance of intergenerational connections beyond the nuclear family grows (14, 16). Examples include intergenerational tutoring to enhance digital literacy, community gardens promoting collaboration, and shared housing or volunteer programs that build social connections. Research shows that these programs not only improve the health and wellbeing of older adults but also foster social cohesion and offer benefits to younger participants as well (17, 18).

A further strategy is to address individual risks through targeted policies or recommendations serving as a key lever for promoting healthy aging.

To use the health sector as an example, the global burden disease (GBD) estimated that in 2023 more than 50% of the disability adjusted life years (DALYs) of the western European population were avoidable (https://www.healthdata.org/research-analysis/g), as they were mainly attributable to five preventable risk factors: tobacco use, hypertension, overweight, high plasma glucose and other nutritional disorders (4). Moreover, a similar risk profile can be found at the global level (19). Similarly, the Organisation for Economic Co-operation and Development (OECD) showed that a significant prevalence of the disease burden in older adults can be prevented by tackling key risk factors across the life course: 45% of dementia cases are caused by 14 modifiable risk factors, as well as insufficient physical activity negatively impacts healthy ageing, increasing the risk of cardiovascular diseases, depression, and several other conditions in later life (20). Putting into practice actions to prevent these determinants could largely reduce the burden of disease in the population and contribute to make sustainable the welfare system.

International organizations [World Health Organisation (WHO), OECD, UN, EU, etc.] are promoting or producing their own scoping reviews of the scientific literature to provide recommendations for promoting healthy aging to their member states, adopting all the different approaches cited below. But these are still scattered across documents, and a systematic collection of them is still lacking.

In this paper, we will focus on these recommendations for actions and policies to produce a collection useful to policy-makers in countries most acutely facing the demographic-epidemiological transition, such as those in Europe.

The work has been developed in the context of a large Italian research project (AGE-IT, https://www.ageit.eu/wp/), in which a work package is focused on the alignment of policies on active and healthy ageing. This paper presents the results of a review of documents of European interest, prepared by international and European organizations that recommended actions and policies to promote active and healthy aging (AHA).

## 2 Methods

### 2.1 Study design

We conducted a scoping review of the reports published by international and European Agencies and Institutions, focusing on the problem of the ageing of the population, looking for recommendations of policies intended to promote AHA. We assumed a definition of policies as actions, regulations, programs, and action plans developed in both national contexts (e.g., a minimum pension) and local contexts (e.g., the installation of water fountains in urban development). Since this is not a systematic review, the protocol was not registered and is not available in English.

### 2.2 Inclusion criteria for reports

We included all papers and documents targeted to policymakers published by an international or European body, published from 2008 to 2023.

Eligible papers were analyzed to extract recommendations for policies that correspond to these criteria:

- targeted to adults, older people, professionals, decision-makers (e.g., national and local politicians, managers and directors of public services, local health authorities), or had an impact on all populations;
- aimed to promote AHA;
- providing examples of actions, legislation, regulations, programs, or plans;
- targeted to European countries, even if not exclusively;

We excluded policies that were oriented to children or young people (e.g., increasing low-cost nursery spots to foster the female workforce as part of an action to promote paid work).

### 2.3 Information source and search strategy

We conducted a Google search for reports and papers. Scientific databases such as Medline were excluded because we were only looking for reports targeted to decision-makers to adopt policies to promote AHA. We used the following search string: ‘site (europa.eu OR who.int OR oecd.org OR un.org) AND (policy OR normative OR priorities OR strategy OR plan OR regulation OR framework OR reforms) AND (“active aging” OR “healthy aging” OR “active and healthy aging”). The search was launched on the 30th of August, 2023. Additional documents were identified using a snowball method through references from records found via Google searches and by consulting the websites of international agencies engaged in health and active ageing promotion.

### 2.4 Selection and data extraction

A reviewer selected the reports to include based on the titles obtained through the Google search. Four reviewers evaluated the inclusion of records for full-text consultation under a double-blind protocol. The included reports were then reviewed to extract the recommendations for actions or policies (hereinafter referred to as actions) by two of us independently, and conflicts were discussed and resolved within the wider project team.

We collected the following data for each policy included: the general goal of the AHA domain, an example of policy (law, action, program, plan, intervention), the target population (adults, adults and old age people, old age people, people with disability/chronic disease, young and old age people, professionals/decision-makers), and the reference from which the example was extracted. Double records were removed during the data extraction double-comparison process.

### 2.5 Analysis and synthesis

To describe the policy sector of action, we used the Comparative Agendas Project’s typology (21). Actions were then classified into 14 Strategies, defined as a group of cross-sectoral actions targeted to a broad goal of the AHA concept, by the authors, after consultation with a panel of experts. To every action, up to four Strategies were assigned. See Supplementary material for a complete list of Strategies and related actions.

Data were analyzed using descriptive tables, which showed absolute and relative prevalence by the type of aim, strategy, and policy sector. Supplementary material shows the definition of strategies, the actions included in each strategy, and the reports from which they have been extracted.

## 3 Results

**Figure 1:**
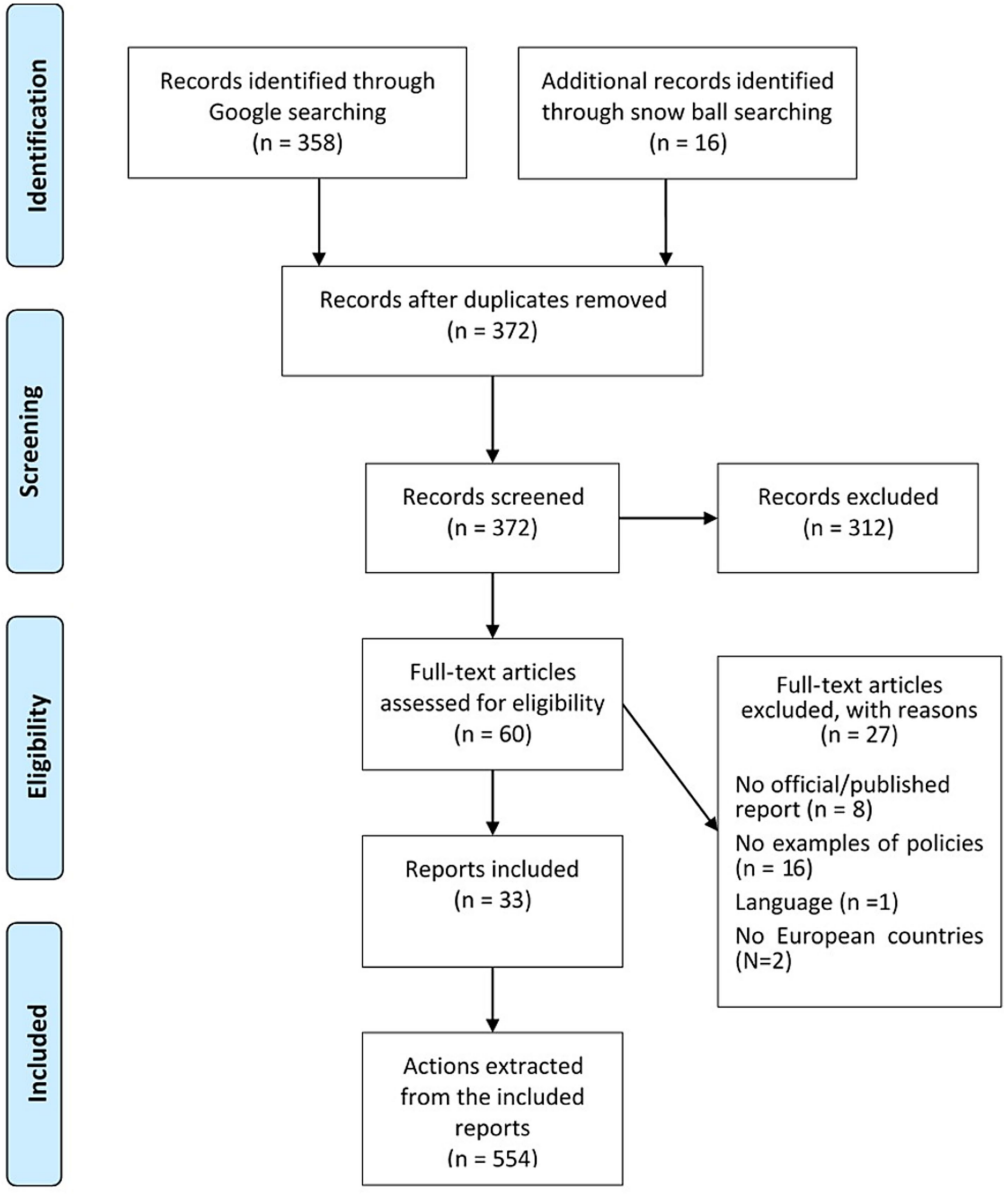
PRISMA flow diagram of the selection process.

The Google search strategy resulted in 296 records retrieved, of which 15 were added through snowball searching. After deduplication, 233 abstracts were submitted to a preliminary review, and 178 were excluded for not relevance. At the end of the process, 55 reports remained (Figure 1). From them, the 33 reports published from 2008 to 2023 were included (Table 1).

**Table 1:** Results of the selection and extraction process.

	*N*
Number of reports included	33
Time period of publication	2008–2023
Number of actions	554
Number of strategies	14
Number of policy sectors	19

All in all, relevant reports were published by a limited number of international or European organizations, including, United Nations (UN), OECD, WHO, European Commission (EC), comprising EC Directorates and European projects, like for example the European Observatory for Health System and Policies (22) (see Supplementary material for the full list of reports included).

The reports were independently reviewed by two reviewers (SC and EV), and the actions recommended to promote AHA were extracted. All in all, 554 actions were extracted and then classified into 19 policy sectors and 14 strategies by two authors independently (SC and EV) (Table 1). In case of disagreement, a third reviewer was consulted. Figure 1 shows the complete selection process and the reasons for exclusion.

We found actions linked to all policy sectors except Defence and Public Lands policies (19 sectors out of 21). Health actions accounted for over 37.5% of all actions (*N* = 208), followed by labor (*N* = 86, 15.5%), social welfare (*N* = 45, 8.1%), and civil rights (*N* = 39, 7.0%) (Table 2). Conversely, we found only one action pertained to international affairs and <5 to Culture and Foreign trade. Housing accounted for 6.5% of actions, with Immigration, Law and crime, and Culture accounting for about 1.0% (Table 2).

**Table 2:** Distribution of actions in different policy sectors by target population (*N* = 554).

Policy sector	Target *N* (%)			Total *N* (%)
	All population	Older people	Professionals/policy-makers	
Macroeconomics	14 (73.7%)	3 (15.8%)	2 (10.5%)	19 (3.4%)
Civil rights	12 (30.8%)	20 (51.3%)	7 (17.9%)	39 (7.0%)
Health	109 (52.4%)	62 (29.8%)	37 (17.8%)	208 (37.5%)
Agriculture	7 (100.0%)	–	–	7 (1.3%)
Labor	56 (69.8%)	21 (24.4%)	5 (5.8%)	86 (15.5%)
Education	6 (54.5%)	3 (27.3%)	2 (18.2%)	11 (2.0%)
Environment	9 (81.8%)	2 (18.2%)	–	11 (2.0%)
Energy	5 (100.0%)	–	–	5 (0.9%)
Immigration	3 (60.0%)	–	2 (40.0%)	5 (0.9%)
Transports	8 (53.3%)	6 (40.0%)	1 (6.7%)	15 (2.7%)
Law and crime	2 (33.3%)	3 (50.0%)	1 (16.7%)	6 (1.1%)
Social welfare	19 (42.2%)	25 (55.6%)	1 (2.2%)	45 (8.1%)
Housing	11 (30.6%)	24 (69.4%)	–	36 (6.5%)
Domestic commerce	14 (60.9%)	9 (39.1%)	–	23 (4.1%)
Technology	2 (16.7%)	6 (50.0%)	4 (33.3%)	12 (2.2%)
Foreign trade	2 (100.0%)	–	–	2 (0.4%)
International affairs	1 (100.0%)	–	–	1 (0.2%)
Governmental operations	4 (21.0%)	6 (31.6%)	9 (47.4%)	19 (3.4%)
Culture	2 (50.0%)	2 (50.0%)	–	4 (0.7%)
Total	290 (52.3%)	193 (34.8%)	71 (12.8%)	554

A large portion of the sample selected, 52.3%, consisted of actions targeted to the general population (Table 2), particularly in some policy areas where these types of actions were prevalent. Activities connected to the foreign trade sector, Energy, or Agriculture were completely aimed at the entire population; Macroeconomic policies proposed were mostly aimed at this target (73.7%). Conversely, Technology policies, civil rights, law and crime, social welfare, and housing were mainly proposed for older people. Finally, 17.9% of health interventions were recommended to professionals and/or decision-makers (Table 2).

The analysis of the strategies presented in Table 3 can be helpful to policy-makers who want to propose AHA policies.

**Table 3:** Distribution of actions by policy sector and strategy (*N* = 554).

Policy sector	*N* (%)*	Tackling socio-economic divide (*N* = 146, 26.3%)*	Reduction of the burden of NCD (*N* = 129, 23.3%)*	Access to quality health and social care (*N* = 150, 17.1%)*	Support for a longer working life (*N* = 68, 12.3%)*	Support permanence at home (*N* = 54, 9.7%)*	Social and cultural engagement (*N* = 54, 9.7%)*	Market regulation (*N* = 30, 5.4%)*	Home and cities adaptation (*N* = 56, 10.1%)*	Environmental interventions (*N* = 30, 5.4%)*	Communication. Media and advertising (*N* = 93, 16.8%)*	Fostering better laws (*N* = 76, 13.7%)*	Monitoring and evaluation for better policy making (*N* = 53, 9.6%)*	Research and development (*N* = 23, 4.1%)*	Miscellanea (*N* = 13, 2.3%)*
Health	208 (37.5%)	30 (20.5%)	69 (53.5%)	89 (59.3%)	8 (11.8%)	22 (40.7%)	9 (16.7%)	7 (23.3%)	10 (17.9%)	5 (16.7%)	33 (35.5%)	25 (32.9%)	33 (62.3%)	11 (47.8%)	2 (15.4%)
Labor	86 (15.5%)	28 (19.2%)	17 (13.2%)	14 (9.3%)	48 (70.6%)	9 (16.7%)	2 (3.7%)	1 (1.3%)	2 (3.6%)	2 (6.7%)	6 (6.5%)	7 (9.2%)	1 (1.9%)	1 (4.3%)	–
Social welfare	45 (8.1%)	23 (15.7%)	2 (1.6%)	17 (11.3%)	–	8 (14.8%)	12 (22.2%)	–	2 (3.6%)	1 (3.3%)	8 (8.6%)	2 (2.6%)	1 (1.9%)	1 (4.3%)	–
Housing	36 (6.5%)	9 (6.2%)	3 (2.3%)	2 (1.3%)	–	14 (25.9%)	11 (20.4%)	–	27 (48.2%)	3 (8.3%)	3 (3.2%)	2 (2.6%)	1 (1.9%)	–	–
Civil rights	39 (7.0%)	21 (14.4%)	1 (0.8%)	8 (5.3%)	5 (7.4%)	–	5 (9.3%)	–	1 (1.8%)	–	6 (6.5%)	12 (15.8%)	5 (9.4%)	4 (17.4%)	5 (38.5%)
Domestic commerce	23 (4.1%)	5 (3.4%)	12 (9.3%)	3 (2.0%)	1 (1.5%)	1 (1.9%)	5 (9.3%)	9 (30.0%)	2 (3.6%)	1 (4.3%)	6 (6.5%)	3 (3.9%)	1 (1.9%)	–	2 (15.4%)
Macroeconomics	19 (3.4%)	5 (3.4%)	9 (7.0%)	1 (0.7%)	5 (7.4%)	–	1 (1.9%)	6 (20.0%)	–	–	1 (1.1%)	4 (5.3%)	–	–	–
Governmental operations	19 (3.4%)	8 (5.5%)	–	–	–	–	–	–	–	2 (6.7%)	4 (3.2%)	8 (10.5%)	10 (18.9%)	1 (4.3%)	–
Transports	15 (2.7%)	3 (20.0%)	2 (1.6%)	2 (1.3%)	–	–	2 (3.7%)	–	10 (17.9%)	–	3 (3.2%)	2 (2.6%)	–	1 (4.3%)	–
Technology	12 (2.2%)	5 (3.4%)	–	5 (3.3%)	–	–	2 (3.7%)	1 (1.3%)	1 (1.8%)	–	7 (7.5%)	2 (2.6%)	–	3 (13.0%)	–
Environment	11 (2.0%)	2 (1.4%)	3 (2.3%)	–	–	–	–	–	1 (1.8%)	10 (33.3%)	4 (3.2%)	3 (3.9%)	1 (1.9%)	–	–
Education	11 (2.0%)	5 (3.4%)	2 (1.6%)	3 (2.0%)	1 (1.5%)	–	1 (1.9%)	–	–	–	2 (2.1%)	1 (1.3%)	–	–	–
Agriculture	7 (1.3%)	–	6 (4.7%)	1 (0.7%)	–	–	–	5 (16.7%)	–	1 (3.3%)	4 (3.2%)	3 (3.9%)	–	–	–
Law and crime	6 (1.1%)	1 (0.7%)	1 (0.8%)	–	–	–	–	–	–	–	2 (2.1%)	2 (2.6%)	–	–	2 (15.4%)
Immigration	5 (0.9%)	1 (0.7%)	–	5 (3.3%)	–	–	–	–	–	–	1 (1.1%)	–	–	–	1 (7.7%)
Energy	5 (0.9%)	–	–	–	–	–	–	–	–	5 (16.7%)	–	–	–	–	–
Culture	4 (0.7%)	–	–	–	–	–	4 (7.4%)	–	–	–	3 (3.2%)	–	–	–	–
Foreign trade	2 (0.4%)	–	2 (1.6%)	–	–	–	–	1 (1.3%)	–	–	–	–	–	1 (4.3%)	–
International affairs	1 (0.2%)	–	–	–	–	–	–	–	–	–	–	–	–	–	1 (7.7%)

*Percentages are calculated on the total of actions included (*N* = 554); the percentages in the main table body are column percentages.

### 3.1 Strategy 1: tackling the socio-economic divide

This study aimed at 146 actions to tackling the social-economic divide, such as ensuring equitable access to care and affordable housing, providing subsidies for home modifications and adaptation for long-term care, and improving the social protection system by ensuring minimum pensions rather than supporting informal caregivers, like credit pension or increasing men’s participation in family work. Health and labor sectors accounted for 20% of these measures (20.5% and 19.2%, respectively). Social welfare (15.7%) and civil rights sectors (14.4%) were also well represented (Table 3). Table 4 shows that 28.8% of these actions were classified solely in this Strategy category, while 26.0% were also Strategies in support of Access to quality health and social care, 17.1% were also in Support permanence at home, 13.0% in Communication, media and advertising, and 8.9% in Home and cities adaptation.

**Table 4:** Interconnections between strategies.

Strategies	Tackling socio–economic divide*	Reduction of the burden of NCD*	Access to quality health and social care*	Support for a longer working life*	Support permanence at home*	Social and cultural engagement*	Market regulation*	Home and cities adaptation*	Environmental interventions*	Communication, media, and advertising*	Fostering better laws*	Monitoring and evaluation for better policy making*	Research and development*	Miscellanea*	Total
Tackling the socio–economic divide	**42 (28.8%)**	10 (6.8%)	38 (26.0%)	17 (11.6%)	25 (17.1%)	11 (7.5%)	2 (1.4%)	13 (8.9%)	3 (2.0%)	19 (13.0%)	18 (12.3%)	9 (6.2%)	1 (0.7%)	3 (2.0%)	146
Reduction of the burden of NCD	10 (7.7%)	**34 (26.4%)**	9 (7.0%)	9 (7.0%)	5 (3.9%)	4 (3.1%)	25 (19.4%)	8 (6.2%)	8 (6.2%)	32 (24.8%)	17 (13.2%)	7 (5.4%)	1 (0.8%)	1 (0.8%)	129
Access to quality health and social care	28 (25.3%)	9 (6.0%)	**53 (35.3%)**	6 (4.0%)	27 (18.0%)	8 (5.3%)	3 (2.0%)	5 (3.3%)	5 (3.3%)	24 (16.0%)	10 (6.7%)	7 (4.8%)	4 (2.7%)	4 (2.8%)	150
Support for a longer working life	16 (23.5%)	9 (13.2%)	6 (8.8%)	**31 (45.6%)**	8 (11.8%)	1 (1.5%)	0 (0.0%)	0 (0.0%)	1 (1.5%)	2 (2.9%)	7 (10.3%)	1 (1.5%)	2 (2.9%)	0 (0.0%)	68
Support permanence at home	25 (46.3%)	5 (9.3%)	27 (50.0%)	8 (14.8%)	**1 (1.8%)**	3 (5.6%)	0 (0.0%)	12 (22.2%)	2 (3.7%)	10 (18.5%)	4 (7.4%)	1 (1.8%)	1 (1.8%)	0 (0.0%)	54
Social and cultural engagement	11 (20.4%)	4 (7.4%)	8 (14.8%)	1 (1.8%)	3 (5.6%)	**16 (29.6%)**	1 (1.8%)	12 (22.2%)	2 (3.7%)	13 (24.1%)	4 (7.4%)	2 (3.7%)	2 (3.7%)	0 (0.0%)	54
Market regulation	2 (6.7%)	25 (83.3%)	3 (10.0%)	0 (0.0%)	0 (0.0%)	1 (3.3%)	**1 (3.3%)**	0 (0.0%)	0 (0.0%)	7 (23.3%)	7 (23.3%)	0 (0.0%)	1 (3.3%)	0 (0.0%)	30
Home and cities adaptation	13 (23.2%)	8 (14.3%)	5 (8.9%)	0 (0.0%)	12 (21.4%)	12 (21.4%)	0 (0.0%)	**17 (30.4%)**	7 (12.5%)	3 (5.4%)	5 (8.9%)	2 (3.6%)	0 (0.0%)	0 (0.0%)	56
Environmental interventions	3 (10.0%)	8 (26.7%)	5 (16.7%)	1 (3.3%)	2 (6.7%)	2 (6.7%)	0 (0.0%)	7 (23.3%)	**8 (26.7%)**	4 (13.3%)	6 (20.0%)	1 (3.3%)	0 (0.0%)	0 (0.0%)	30
Communication, media, and advertising	19 (20.4%)	32 (34.4%)	24 (25.8%)	3 (3.2%)	10 (10.8%)	13 (14.1%)	7 (7.5%)	3 (3.2%)	4 (4.3%)	**8 (8.6%)**	8 (8.6%)	8 (8.6%)	3 (3.2%)	2 (2.2%)	93
Fostering better laws	18 (23.7%)	17 (22.4%)	10 (13.2%)	7 (9.2%)	4 (5.3%)	4 (5.3%)	7 (9.2%)	5 (6.7%)	6 (7.9%)	8 (10.5%)	**17 (22.4%)**	8 (10.5%)	6 (7.9%)	2 (2.6%)	76
Monitoring and evaluation for better policy making	9 (17.0%)	7 (13.2%)	7 (13.2%)	1 (2.0%)	1 (2.0%)	2 (3.8%)	0 (0.0%)	2 (3.8%)	1 (2.0%)	8 (15.1%)	8 (15.1%)	**21 (39.6%)**	2 (3.8%)	1 (2.0%)	53
Research and development	1 (4.3%)	1 (4.3%)	4 (17.4%)	2 (8.7%)	1 (4.3%)	2 (8.7%)	1 (4.3%)	0 (0.0%)	0 (0.0%)	3 (13.0%)	6 (26.1%)	2 (8.7%)	**7 (30.4%)**	0 (0.0%)	23
Miscellanea	3 (23.1%)	1 (7.7%)	4 (30.8%)	0 (0.0%)	0 (0.0%)	0 (0.0%)	0 (0.0%)	0 (0.0%)	0 (0.0%)	2 (15.4%)	2 (15.4%)	1 (7.7%)	0 (0.0%)	**4 (30.8%)**	13

*Percentages were calculated on the total column, reporting the row amounts; the column total exceeds 100% because each action can be classified under more than one category and up to a maximum of four strategies. The diagonal shows actions classified in only one strategy. The numbers in bold correspond to the diagonal.

### 3.2 Strategy 2: reduction of the burden of non-communicable diseases (NCDs)

In total, 23.3% of actions (*N* = 129) promoted the reduction of the burden of NCDs; they ranged from prevention actions, such as increasing sobriety checkpoints and smoke-free areas or restricting advertising of unhealthy foods, to air quality recommendations, supporting agreements between gyms and enterprises to promote physical exercises in workplace settings rather than improving communication flows between researchers and decision-makers. Health policies accounted for clearly half of the activities in this strategy, but so did labor policies (13.2%), domestic commerce (9.3%), macroeconomics (7.0%), and agricultural policies (4.6%) (Table 3). Table 4 reveals that a quarter of these measures were classified only as Reduction of the burden of NCD (26.4%), while the other quarter was categorized as well as Communication and media initiatives (24.8%). About 19% were also Market regulation strategies, with 13.2% recommending the Development of better legislation.

### 3.3 Strategy 3: access to quality health and social care

In total, 150 actions (27.1%) aimed to improve access to quality health services, primarily through interventions in the sectors of health (59.3%) and social welfare (11.3%), but also in the sectors of labor (9.3%), civil rights (5.3%), technology (3.3%), domestic commerce (2.0%), immigration (3.3%), transportation (1.3%), housing (1.3%), and agriculture (0.7%) (Table 3). Table 4 illustrates the interconnections among strategies, since every action could be classified into up to 4 strategies. Among them, 35.3% of actions included in the strategy for access to quality services were related solely to this strategy; however, 25.3% of them contributed to tackling the socio-economic divide, 18.0% to supporting permanence at home, and 16.0% to communication and media interventions.

### 3.4 Strategy 4: support for a longer working life

To keep older people in the paid labor market, international agencies recommended 68 actions such as adapting work practices and working time to workers’ aging, promoting job-sharing, part-time work, working from home, and flexible working practices, rather than supporting mobility of older workers across labor sectors. Significant social security activities included ensuring the right to work beyond the pensionable age, discouraging the mandatory retirement age, with work-time and mansion adaptation, or long-term leave, and reducing working hours (mixed income or partial retirement plans). Clearly, almost all initiatives indicated to enhance longer working life were of the labor sector (70.6%), but we also found a few policies contributing to the health sector (11.8%) (Table 3). Half of these actions were classified only as this approach (45.6%); it is worth noting that 23.5% were also strategies tackling the socio-economic divide, and 13.2% in support of NCDs burden of disease reduction (Table 4).

### 3.5 Strategy 5: support permanence at home

In this study, 54 activities were proposed to promote older people’s ability to stay at home, with the majority (40.7%) being focused on health policies relating to long-term care in the community and home assistance, integrated care, and person-centered pathways. 25.9% of the actions were related to the Housing sector, such as subsidies for home adaptation, including heating and fuel efficiency, designing homes to facilitate community inclusion, addressing later-life homelessness, and planning and developing dementia-friendly communities. Social welfare and labor policy accounted for approximately 15% of them (14.8 and 16.7, respectively). Among these two last sectors, we found 14 actions (25.9%) that support informal caregivers, such as financial assistance for informal carers of older people, raising the limit on the number of hours paid work outside the home, flexible working time arrangements to combine work and caregiving for older workers, and implementing caregiver relief. Only 1.8% of these actions were unique to this strategy. Conversely, half of the measures (50.0%) were strategies to improve Access to quality health and social care, with the other half (46.3%) aimed at Tackling socio-economic divide. Finally, about 20% included home/cities adaptation and/or Communication and media initiatives (22.2 and 18.5%, respectively) (Table 4).

### 3.6 Strategy 6: social and cultural engagement

The sample’s 54 actions aimed at culture and social interaction were primarily focused on social welfare (22.2%), Housing policies (20.4%), and health (16.7%). This approach included recommendations to increase leisure opportunities and accessibility, as well as to expand and promote older people’s organizations, intergenerational relationships, and multigenerational co-housing. Approximately a third of these measures were only classified as part of this strategy (29.6%), whereas roughly a quarter were also communication, media, and advertising (24.1%), followed by house and cities adaptation (22.2%) and tackling socio-economic divide strategies (20.4%) (Table 4).

### 3.7 Strategy 7: market regulation

These actions (*N* = 30) are mainly focused on reducing the burden of NCD (83.3%), such as limiting the availability and/or advertising of alcohol, tobacco, and unhealthy foods and drinks (Table 4).

### 3.8 Strategy 8: home and city adaptation

In this study, 56 activities promoted home, community, and city adaptation, with a prevalence of housing policies (48.2%); examples are urban design of public areas and residences enabling community involvement or physical activity, while also ensuring accessible and inexpensive public transportation (17.9%). Actions connected to this strategy were predominantly classified in this group (30.4%) or in combination with Support permanent at-home strategy, Social and cultural engagement, and tackling socio-economic divide (all around 20%) (Table 4).

### 3.9 Strategy 9: environmental interventions

The 30 actions are mainly classified in the health (16.7%) and Environment (33.3%) sectors, and are focused on climate protection.

### 3.10 Strategy 10: communication, media, and advertising

Recommendations promoting communication (including social marketing and advertising control) represents the 16.8% of the total sample, mostly in the health sector (*N* = 33, 35.5%) (Table 3), and in the Fostering better laws strategy, which accounted for 13.7%. Among them, 24.8% addressed NCDs, 16.0% supported Access to quality health and social care, 12.3% reduced the tackling of the socio-economic divide, and 24.1% also encouraged social and cultural participation (Table 4).

### 3.11 Strategy 11: fostering better laws

Among this last strategy, we gathered recommendations for how to act in support of AHA, such as supporting the narrative to foster the inclusion of ageing in all policies, assisting trade unions in refocusing on ageing, planning for a national program in ageing and health, designing win-win solutions, or aligning with international standards, guidelines, programs, and protocols such as the European Sustainable and Smart Mobility Strategy or the EU recommendations on air quality. Once again, the health sector had the highest representation (32.9%), followed by civil rights (15.8%), Government operations (10.5%), and labor (9.2%) (Table 3). Moreover, 22.4% of measures promoting better laws were only classified in this strategy; another 22.4% were also indicated for reducing the burden of NCDs and for tackling the socio-economic divide (23.7%) (Table 4).

### 3.12 Strategy 12: monitoring and evaluation for better policymaking

Most part (62.3%) of them (*N* = 53) were classified into the health sector (Table 3). The other Strategies in which they are classified are Tackling socio-economic divide, Reduction of burden of NCD, and Access to quality health and social care (17.0, 13.2 and 13.2% respectively) (Table 4).

### 3.13 Strategy 13: research and development

In this study, 23 actions contribute to this strategy, mainly included in the health, civil rights, and technology sectors (47.8, 17.4, and 13.0% respectively). 26.1% of them are also classified in the Fostering better laws strategy (Table 4).

### 3.14 Strategy 14: miscellanea

A few actions (*N* = 13) did not fit any strategy classification and are put in the Miscellanea group. They include actions plans for humanitarian emergencies, support of immigration in specific contexts, actions to respond to older people abuse and others (Supplementary material).

Tables in the Supplementary material list all the actions classified in their strategies.

## 4 Discussion

Welfare systems in industrialized countries undergoing demographic transition need to adapt their welfare system urgently in order to become more resilient. International authorities, such as the WHO, EU, and OECD, have developed in recent years a body of recommendations targeted at policymakers, which is still not organized.

Our study has the ambition to contribute to the process of adaptation of national welfare systems by extracting and organising the recommendation proposed by the most influential international authorities to make them more easily accessible. This is intended as a preliminary task to make facilitate the identification of possible gaps of the European Countries regulatory systems, to adapt them to the demographic transition.

The study resulted in a body of 554 distinct actions or policies across 19 policy sectors, organized into 14 strategies. In accordance with the objectives of the study, they are actions or policies aimed at promoting AHA, and do not include actions aimed at people with a disease, for example, the hospital or territorial management of chronic diseases and frailty, or access to services for diagnosis and treatment.

Most parts of the economic sectors are involved. If health is the sector most involved with 208 pertinent actions, other sectors are largely represented. For example: labor (86 recommendations), social welfare (45), housing (36), and civil rights (39). This broad representation of economic sectors demonstrates that adapting to an ageing population in European countries requires a profound intersectoral rethinking of the role of the State.

The strategy focused on primary prevention of diseases (Reduction of the burden of NCD) includes 129 actions, whereas the strategy promoting Access to quality health and social care includes 150. They range from individual interventions, e.g., promotion of behavioral intervention for muscle preservation, to the recommendation of tax benefits to increase physical activity or for active mobility.

Sixty-seven actions are aimed at Supporting a longer working live, as an essential element of the healthy and active ageing, together with an important element of welfare sustainability; they range from recommendations to have more flexible working time arrangements to combine work and caregiving, and flexible retirement schedules to training and retraining to adapt the needs of older workers, and to mixed income combinations of paid work and pensions to allow progressive reduction of working hours.

A further relevant strategy is Support permanence at home, collecting actions to support the permanence of older people in their home, and to prevent their institutionalization. It includes interventions to adapt apartments to enable older people to age in the place that is right for them, actions to facilitate the work of informal caregivers, to ensure to them a legal status and to reduce their costs as well, to promote autonomy and health. Financial aid is also a frequent option recommended by documents.

A relevant group of actions is included in two very significant strategies: Fostering better laws and Monitoring and evaluation for better policy making. These two strategies, including 76 and 53 actions respectively, itemize principles that should be a base for good laws, as for example aligning AHA actions to green and blue transitions strategies, designing intergenerational win-win solutions, combining welfare policies for both older and younger people.

The strategy of tackling the socio-economic divide aims to increase equity. The intervention to promote AHA showed a geographical trend in Italy (23), favoring Northern areas, whereas the evidence indicates that older people with middle- and higher-socioeconomic status have higher levels of behavioral promotion of AHA (24).

The list of recommendations we elaborate from the international authorities can be considered as benchmark for national policies, and the implementation at a large scale of them can contribute to the reduction of inequalities among countries and regions. The intrinsic multisectoral approach is essential to promote environmental conditions that favor behavior change. For example, physical activity can be improved by interventions at the level of occupation, of leisure time, of urban organization, and of housing.

There are several limitations of this study. The first is the timeframe: we decide to focus on a window of 15 years, but the debate about the need of policy-adaptations to population ageing is much older. For example, the Second World Assembly on Ageing, which adopted the Madrid International Plan of Action on Ageing (MIPAA) in 2002 (25). Also in 2002, the European Commission and the United Nations Economic Commission for Europe (UNECE) developed the Active Ageing Index (AAI), a tool to monitor the progress of policies across Europe (26, 27). But these reports are largely background to the work done in the following years, and we are confident that the main topics are represented in our work. The second limitation is the lack of evidence of effectiveness in most of the actions collected. We conducted an *a posteriori* search for evidence, but we found explicit citations to literature only for approximately 50% of actions, and only a smaller part was an evaluation study. For this reason, the actions presented in this paper cannot be defined as evidence-based recommendations, but at most as good practices. In essence, we have to rely on the authority of the producing bodies. This limitation does not facilitate the identification of strategies and actions to be implemented as a priority, and for this reason will be the focus of a further work to be done.

Another limit of the study is to have excluded from the objective actions and policies addressed to the improvement of treatment facilities, of nursing homes and in general to the chronic diseases. However, this was a preliminary choice to limit our research to interventions promoting healthy and active aging, mainly of a preventive nature. The legislation of European Countries is still very much focused on responding to the health needs of sick people, disability, frailty, etc.

However, this study does have some strengths. The first is the proposal to classify actions into strategies. The action-based strategy framework is probably the most important contribution of this study. The concept of strategy developed in the context of this project has the aim to foster multi-sectorial approaches of policy making by grouping meaningful actions that can contribute to major objectives of AHA such as prevention of chronic disease, fostering a longer occupational life, etc. (28). Strategies are cross-sectoral in their essence, because they involve several sectors of the public policy to reach specific objective. This is the richness but also the complexity of this work. Other frameworks of actions and policies have been proposed to organize recommendations, as that proposed in 2002 by UNECE (26)—composed by 10 Commitments, from Mainstreaming ageing in all policy fields, to Promote regional co-operation—or that proposed by Lucanthone in 2022, grouping actions into 7 Challenges, from Narrow policy approach, to Mainly work oriented approach (28). Both classifications were intended to be general enough to allow their use in different countries and at different levels of government, from national to local ones. Our proposal to group the actions in strategies is based on the need to be as concrete as possible in order give to policy-makers evidence-based recommendations easy to be adopted. For this reason, they are not alternatives, but rather complementary.

In our mind, a further strength of this study, is to have focused the general topic of the promotion of active and healthy ageing. In some EU countries debate on ageing population is often limited to and confused with that of chronicity and the increase of prevalence of chronic patients. This is the case, for example, of Italy, especially before the adoption of an organic legislative reform in 2023–24 (29). The need to increase personnel to manage chronic patients, the emergence of telemedicine and of the long-term care, are the most frequent topics of debate. Several authors underline that further improvements of care for chronic patient leads to the paradoxical result of increasing the demand, as well as the costs of care (30, 31). Only an approach prioritizing the promotion of AHA can contribute to the sustainability of the welfare and to the wellbeing of the whole population.

In conclusion, the adaptation to the population ageing is a priority of many countries undergoing a dramatic epidemiologic and demographic transition. Over the past years, international agencies have devoted significant effort to developing numerous policies and actions to promote AHA.

But, in our knowledge, there is currently no a reference documentation yet summarizing all the needed policy adaptations in all sectors involved in the promotion of AHA. In this study, we collected 554 different actions addressed to almost all policy sectors, in which we recognized 14 main strategies. This body of recommendations for policies and actions can be of great help as reference for the national, regional and local administrations in the elaboration strategies to react to the acute problem of ageing of the population.

Only a small number of actions proposed by the source documents are provided with robust evidence of effectiveness. Most are rational responses to factors proven to be risk factors of unhealthy ageing trajectories. Despite this, which potentially weakens the single recommendation, we believe this effect only marginally reduces the utility of our comprehensive work.

On the other hand, the limited availability of scientific evidence supporting the single recommendation emphasizes the need for an effort in elaborating a framework of assessment methods to evaluate at least the most important actions included in this report.

## Data Availability

The original contributions presented in the study are included in the article/Supplementary material; further inquiries can be directed to the corresponding author.
