# Supplementary material for: Policies and strategies on active and healthy ageing: a scoping review of the recommendations of European and international agencies
Source: Front Public Health. 2026 Jan 7;13:1712417. doi: 10.3389/fpubh.2025.1712417 (PMC12819296; doi:10.3389/fpubh.2025.1712417)
Supplement: Supplementary file 1 [file Data_Sheet_1.PDF]

Supplementary Materials

1 Detailed list of Strategies and definitions

Strategy	Aim
Strategy 1: Tackling socio-economic divide	To fill the gap between the socio-economically advantaged and disadvantaged older people, fostering greater equity and inclusivity within the society, and reducing disparities among people.
Strategy 2: Reduction of the burden of NCD	To prevent or manage non-communicable diseases (NCDs), enhance overall public health outcomes and promote healthier lifestyles.
Strategy 3: Access to quality health and social care	To guarantee that older people can obtain high-quality healthcare and social services.
Strategy 4: Support for a longer working life	To enable older workers to remain productive, maintain financial stability, and stay active for as long as possible.
Strategy 5: Support permanence at home	To promote independence, comfort, and a higher quality of life for older adults, enabling them to remain at home for as long as possible.
Strategy 6: Social and cultural engagement	To promote social inclusion, enhance well-being, and foster a sense of belonging by keeping individuals connected to their communities and engaged in meaningful interactions.
Strategy 7: Market regulation	To create a more inclusive and equitable market environment for ageing populations, protecting and ensuring fair access in various markets (e.g. financial services, housing, healthcare, goods…).
Strategy 8: Home and cities adaptation	To enhance independence, safety, and overall quality of life for older adults in their living environment (homes, cities and communities).
Strategy 9: Environmental interventions	To improve environmental quality, addressing current challenges and by creating healthier and more sustainable environments.
Strategy 10: Communication, media, and advertising	To provide correct and suitable information that supports well-being, ability to make informed choices, support access to services, and enhances quality of life.
Strategy 11: Fostering better laws	Advocating for legislative changes to enhance the well-being and protect the rights of older adults.
Strategy 12: Monitoring and evaluation for better policy making	To create and implement policies that effectively address the needs of the ageing population and promote their overall well-being.
Strategy 13: Research and Development	To advance knowledge and create innovative solutions to enhance the quality of life for older adults.
Strategy 14: Miscellanea	To address a diverse range of needs and challenges that do not fall under specific, predefined categories, ensuring comprehensive support and solutions for various issues impacting the target population.

2 Detailed list of actions, stratified by strategies

Strategy 1: Tackling socio-economic divide

ID	Actions	Policy sectors	Target	Report
204	To offer good services in deprived areas	Social Welfare	All population	WHO, 2021b
304	To assess age-friendliness of communities and apply an equity lens	Health	Old age people	WHO, 2019c
377	To promote sanitation, heating and fuel efficiency in housing, and support home repair, maintenance, assessment and modification through measures such as subsidies	Domestic commerce	Old age people	WHO, 2019c; UN Women, 2023
89	To develop accountability systems, such as the use of equality impact evaluation and indicators, to strengthen personal and community skills	Health	All population	WHO, 2019c
86	To design win-win solutions that benefit both older and younger populations while rejecting zero-sum game narratives	Social Welfare	All population	WHO, 2022b
145	To ensure that captioning is available for TV broadcasts for those with hearing impairments	Technology	All population	WHO, 2015a
419	To reduce air pollution exposure among vulnerable groups, particularly older adults with pre-existing conditions and those from lower socioeconomic backgrounds, through education and communication initiatives	Environment	Old age people	WHO, 2019c
49	To promote bank use safety – this can be monitored to detect suspicious patterns and may help to identify older people at risk of financial abuse	Law and Crime	Old age people	WHO, 2015a
239	to improve older people's health literacy by offering access to resources, offering instruction on the usage of information technology	Education	Old age people	WHO, 2019b; WHO, 2019c
16	A sufficient supply of acceptable and inexpensive housing, which may include market-based housing, low-cost housing, or homes in assisted-living rental communities	Housing	Old age people	WHO, 2015a
461	To ensure the strengthening of legal security of tenure for all the protection of citizens from illegal eviction (for older people)	Housing	All population	WHO, 2019c
302	To create a case for investment by examining and presenting the costs and advantages of pursuing, preventing and reducing the effects of elder abuse	Civil Rights	Old age people	WHO, 2022a
471	To support “Housing First” policies to address later-life homelessness	Housing	Old age people	WHO, 2019c
172	To Establish priority action areas to combat older people abuse	Civil Rights	Old age people	WHO, 2022a
233	To implement policies that support equitable access to quality and affordable housing for older people: social housing, assisted-living facilities, continuing-care communities and shared-living arrangements, including hostels and care homes	Housing	Old age people	WHO, 2015a; WHO, 2019c; WHO, 2021b
200	Free cessation treatments (e.g. smoking cessation medications, behavioural counselling, personalised feedback, physician advice, follow-up)	Health	All population	WHO, 2019a
208	Catastrophic medical expenses must be covered by the health insurance system	Health	All population	WHO, 2015a
500	To target assistance towards those most in need of a healthy diet	Health	Old age people	OECD, 2009
321	Health systems should be centered on the inherent abilities and functional capabilities of the seniors: To meet the requirements of senior citizens, make sure that sectors—especially health and social services—cooperate	Health	Old age people	WHO, 2015b; WHO, 2017b

206	To guide research and innovation to ensure that public and private sector developers and providers (including health and care services, medical devices, and pharmaceuticals) meet the needs of older adults, including those with limited resources	Health	Professionals/policy makers	WHO, 2015b; WHO, 2017b
507	To develop technological and social innovations for home- and community-based services for older populations	Health	Old age people	WHO, 2015a; WHO, 2017b; EC, 2022b
493	To support training programmes for informal carers (e.g. new technologies, access to health services, gerontological skills, combating ageism, etc.)	Social Welfare	All population	WHO, 2012; WHO, 2015b; WHO, 2016; OECD, 2017a; EC, 2018; WHO, 2019c; EC, 2022a
187	To implement financial aid for informal carers of seniors	Social Welfare	All population	UN, 2022
406	To provide food subsidies and non-stigmatizing food banks to improve food security	Social Welfare	Old age people	WHO, 2019c
147	To ensure that information about transport options and timetables are available in accessible formats	Transportation	Old age people	WHO, 2015a
453	To promote social prescribing, which connects patients to local non-clinical sources of support	Social Welfare	Old age people	WHO, 2021a
84	To design housing that facilitates community integration	Housing	All population	WHO, 2015a
203	Global, regional and national responses to climate change should ensure that attention is paid to older people and avoid institutional and interpersonal discrimination according to age	Environment	Old age people	UN, 2022
181	To expand housing options and assist with home modifications that allow older adults to age in a place suited to their needs without financial burden	Social Welfare	Old age people	WHO, 2015b; WHO, 2017b
404	Providing community education about disaster risk-management through brochures, posters, television and radio can be used to increase the visibility of older people and highlight both their needs and capacities and attention to the type of communication made to the seniors (consider sensory loss and low literacy)	Domestic commerce	All population	WHO, 2015a
372	To promote networks among stakeholders to foster ‘age-friendly’ cities and communities: Multi-interest participatory platforms (ensuring adequate representation will allow smaller settlements, such as towns and rural communities, to be included in national discussions, and provide a voice for underrepresented groups, including ethnic minorities, people with disabilities, indigenous populations, and those in low-income neighbourhoods)	Housing	All population	WHO, 2023
298	Loans, grants, or direct transfers (subsidies) for home modifications aimed at enhancing physical accessibility, comfort, and safety should be provided directly to older adults or to landlords whose older residents meet specific criteria, such as income level. Landlords must agree not to raise an older person's rent as a result of these adaptations	Housing	Old age people	WHO, 2015a
396	To provide information in formats such as large print, “easy read” and pictures that meet the needs of older people, enabling them to make free and informed decisions	Health	Old age people	WHO, 2015b;WHO, 2017b
102	To develop tax benefits to promote physical activity	Macroeconomics	All population	EC, 2022a
234	To implement subsidies to increase the intake of fruits and vegetables	Social Welfare	All population	EC, 2022a
499	To support purchasing of healthy foods in low-income population between targeted transfers	Domestic commerce	All population	OECD, 2017a
497	To promote tailored advocacy on healthy ageing	Health	All population	WHO, 2015b; WHO, 2017b;

				WHO, 2021b; UN, 2020
159	To ensure access to essential medicines, vaccines, and innovations by facilitating the early market entry of generic essential medicines	Health	All population	WHO, 2019b
158	To ensure access to essential medicines, vaccines, and innovations aimed at optimising older people's intrinsic capacities and functional abilities, including strategies such as reducing prices for essential medicines	Health	Old age people	WHO, 2015b; WHO, 2017b; WHO, 2019b; UN, 2020; EC, 2021b; WHO, 2021b
300	To establish long-term care insurance, with individuals contributing proportionally to their income through payroll or pension contributions, ensuring coverage is extended to all, regardless of income level or the availability of alternative informal caregiving options	Health	All population	WHO, 2019a; EC, 2021a; EC, 2021b
277	To introduce or strengthen legislation that promotes non-discrimination, on the basis of aspects such as gender, in the provision of health services	Civil Rights	Old age people	WHO, 2019c
8	To acknowledge the right of older people with significant losses of capacity to appropriate care and support, and to enshrine this aspect in national legislation to ensure access to quality services, with special attention to poor and marginalised older individuals	Civil Rights	Old age people	WHO, 2016
276	To introduce or strengthen legislation that promotes non-discrimination, on the basis of aspects such as age, in the provision of health services	Civil Rights	Old age people	WHO, 2019c
226	To determine the economic impact of informal, unpaid labor by include it in jurisdictional estimates	Macroeconomics	All population	WHO, 2019c
165	To equalize the pensionable age of men and women	Labor	All population	WHO, 2019c
420	To lower income-based obstacles to enrolling in pension plans	Labor	All population	WHO, 2019c
519	To provide unemployment insurance	Labor	All population	WHO, 2015a
38	To assure a minimum pension	Labor	Old age people	EC, 2021a
39	To provide a residence-based or assistance-based pension, add previously uninsured workers (including those working informally), and expand social pension	Social Welfare	Old age people	WHO, 2015a; WHO, 2019c; EC, 2021a
62	To mix contributory and non-contributory public pension plans based in legislation or create universal public non-contributory pensions that would permit the upkeep of living standards	Labor	All population	WHO, 2019c
79	Co-payment reductions or exemptions for long-term care	Health	All population	OECD, 2017a
390	To give free or low-cost computer and internet access in, for example, community centers and libraries	Technology	Old age people	WHO, 2015a; WHO, 2019c
340	To address digital literacy: age-inclusive, inexpensive access to the internet	Education	All population	WHO, 2015a; OECD, 2019a; OECD, 2019b; UN, 2020; EC, 2022a
407	To provide social assistance within the home and community for the poorest and oldest individuals (often overlapping categories) and for those who lack family support (for example, through targeted cash transfers delivered within family or community networks)	Social Welfare	Old age people	WHO, 2015a
227	To implement evidence-based older people abuse prevention and response programmes	Civil Rights	Old age people	WHO, 2015b
127	To encourage public debate on healthy ageing and health equity	Government Operations	Professionals/policy makers	WHO, 2019c

154	To ensure that the media presents a balanced portrayal of aging	Civil Rights	All population	WHO, 2016; UN, 2020
465	To strengthen the administrative health capabilities	Health	Old age people	EC, 2022b
520	A universal and comprehensive health system for long-term care, provided at home, in communities, or within institutions, enables older adults who depend on care to continue their daily lives. Universal access to healthcare and social services must be guaranteed, with financial protection mechanisms, including cost-sharing regulations to support low-income families, particularly older individuals. This includes affordable coverage for medicines and assistive devices. Implementing universal health coverage should focus on reducing out-of-pocket payments, extending coverage, and expanding the necessary services for older people. Publicly funded, accessible health services, including healthcare and long-term care, are essential for supporting the well-being of older adults	Health	All population	WHO, 2012; WHO, 2015b; WHO, 2016; OECD, 2017b; OECD, 2019a; OECD, 2019b; WHO, 2019c; UN, 2020; WHO, 2021b
92	To develop and ensure gender-responsive, affordable, accessible, and sustainable mobility by adhering to accessibility standards in buildings and implementing safe transport systems, pavements, and roads	Transportation	All population	UN, 2020
386	To provide accessible publicly funded social services, such as housing, sanitation, food security, and employment services	Social Welfare	All population	WHO, 2019c
273	Information about services should be available in different languages	Immigration	All population	EC, 2022a
207	Health Equity Audits	Health	All population	EC, 2022a
339	Plan to address health literacy using a community-based approach: health education, teach people how to care for themselves and each other as they get older; educate and empower people on the services use; health information and media campaign (development of websites and brochures, and other publicity measures)	Education	All population	WHO, 2015b; WHO, 2017b; OECD, 2017a; OECD, 2019a; OECD, 2019b; UN 2020; EC, 2021b; EC, 2022a
97	Developing efficient, cost-effective, and scalable solutions to ageism: To develop a bundle of cost-effective interventions for all the major forms of abuse and for numerous sectors to prevent and respond to older people abuse; Implementation scientists and applicable toolkits should be used to widely disseminate and scale up interventions	Civil Rights	Old age people	WHO, 2022a
98	To develop national standards, guidelines, protocols and accreditation for provision of community social care (i.e. to promote human rights for older people and their caregivers)	Social Welfare	Old age people	UN, 2020
417	To generate funding to combat ageism and prevent its consequences	Civil Rights	All population	WHO, 2021b; WHO, 2022a
15	To address incentives for costly care for older persons nearing the end of life	Social Welfare	Old age people	WHO, 2019a
36	Annuitization of pensions: assure a high enough replacement rate from annuities by preventing early lump-sum withdrawals on a sufficiently high threshold of pension assets (in contrast to a flat-rate pension)	Macroeconomics	All population	OECD, 2017a; OECD, 2017b
73	In contrast to inequalities in the pension treatment of different types of pensioners: a single framework covering all workers in an identical and financially sustainable fashion, limiting inequality, improving labor mobility, and lowering expenses	Labor	Old age people	OECD, 2017a; OECD, 2017b
337	To arrange a pension system redistribution to compensate for the impact of time spent in employment and caring for children without motivating workers to stay out of work: pension credits	Labor	All population	OECD, 2017a; OECD, 2017b

402	Providing enough public old-age pensions: In cases when required pension system replacement rates are low, raising the mandatory component is preferable to subsidizing voluntary private schemes. Consider redistribution methods in pension plans to account for inequalities in prior wages, such as applying greater replacement rates to individuals with lower labor salaries	Labor	Old age people	OECD, 2017a; OECD, 2017b; WHO, 2019c
496	To ensure survivor benefits and pensions for widows and widowers at a minimum level	Labor	Old age people	OECD, 2017a; OECD, 2017b; WHO, 2019c
506	Tax-funded minimum pensions	Labor	Old age people	UN, 2020; EC, 2021a; WHO, 2021b
349	To plan to reduce health inequalities, specifically by addressing social determinants (i.e. education level)	Education	All population	EC, 2022a
350	To plan to reduce health inequities, specifically by tackling social causes (i.e., poverty)	Social Welfare	All population	EC, 2022a
299	To cover long-term care costs through a comprehensive social protection system, ensuring that out-of-pocket expenses for recipients of home care remain low enough for them to afford other essential living expenses, such as food, housing, and heating	Health	All population	WHO, 2017b; OECD, 2017a; UN, 2020; WHO, 2020; EC, 2021a; EC, 2022b
509	Telehealth services for rural populations or remote monitoring of chronic patients (in rural areas) (cancer patients and survivors)	Health	Old age people	WHO, 2015a; OECD, 2017a; WHO, 2019c; OECD, 2019a; EC, 2021b
457	To steward the development of the infrastructure and support necessary to ensure that long-term care is included under universal health coverage	Health	All population	WHO, 2015a; WHO, 2015b; WHO, 2017b
328	Payment and reimbursement according to paths	Health	All population	EC, 2022b
222	To identify access to long-term care as a public health priority and a human right, including essential aids such as hearing and vision devices, basic mobility equipment (e.g. canes and walkers), personal hygiene tools, and adjustable furniture or bedding solutions	Health	All population	WHO, 2015a; WHO, 2015b
111	To develop new models of care facilities (small-scale living facilities, mixed-care facilities, e-village for people with dementia)	Health	All population	OECD, 2019a
537	To improve accessibility for older adults with mobility issues and reduced intrinsic capacity in healthcare settings by training providers to enhance communication with visually or hearing-impaired individuals	Health	Professionals/policy makers	WHO, 2019c
182	Expand range of activities for insurance	Health	All population	EC, 2022b
220	Horizontal integrated care: coordination among different service levels, health/social services, and across different setting and providers	Health	Professionals/policy makers	OECD, 2009; WHO, 2015a; WHO, 2016; OECD, 2015; WHO, 2021b
414	To implement a basic package of support for home care and informal caregivers, including alternative options for day care	Social Welfare	All population	WHO, 2012
319	To offer community-based service provision for older people in need of long-term care, including those with disabilities	Health	All population	WHO, 2019c; EC, 2021a
190	Flexible academic course scheduling	Education	All population	EC, 2018

286	Leaves for informal caregivers of ill, handicapped, or older people: i.e., respite care	Social Welfare	All population	WHO, 2015a; WHO, 2015b; WHO, 2017b; OECD, 2017b; WHO, 2019c; EC, 2021a; UN Women, 2023
118	Digital interventions include instruction in using the Internet and computers, as well as help for video communication	Social Welfare	Old age people	OECD, 2017b; WHO, 2021a
162	Ensuring that persons with a minority racial or ethnic origin may fully realize their potential would result in improved social and economic consequences for everybody	Civil Rights	All population	EC, 2021a
264	To increase participation of women in paid work	Civil Rights	All population	EC, 2021a
259	To boost employment of persons with disabilities, which needs appropriate accommodations and an accessible work environment	Civil Rights	All population	EC, 2021a
263	To enhance men's engagement in unpaid jobs (such as domestic chores and family care) to minimize gender inequality in the workplace and education	Civil Rights	All population	EC, 2021a; EC, 2022b
442	Facilitate return-to-work programmes for people with mental health problems; tackling stigma and stereotypes, preventing stress, burn-out and bullying; right to disconnect	Labor	All population	EC, 2022a
327	Pay transparency	Labor	All population	WHO, 2022b
258	To encourage and support the labor engagement of persons living with diabetes or CVD	Labor	All population	EC, 2022a
534	To ensure well-designed sickness programs for workers	Labor	All population	OECD, 2017a; OECD, 2017b; EC, 2021b
330	Pension credits for informal caregivers of non-autonomous seniors, recognizing paid and unpaid services as care providers	Social Welfare	All population	WHO, 2019c; WHO, 2021b; EC, 2022b
271	To refocus the informal care workforce on tasks best suited for informal caregivers, such as mental health and spiritual support, rather than traditional formal care tasks	Health	All population	WHO, 2022b
191	Allow flexible working time arrangements to combine work and caregiving for older workers: flextime	Labor	All population	WHO, 2015a; WHO, 2015b; OECD, 2017a; OECD, 2017b; WHO, 2017b; WHO, 2019a; EC, 2021a; EC, 2022b; EC, 2022a; UN Women, 2023
324	To pay informal caregivers: cash-for-care incentives in the form of vouchers, home-care grants, direct payments, personal budgets, tax refunds, or cash transfer programs	Social Welfare	All population	WHO, 2015a; WHO, 2016; OECD, 2017b; WHO, 2019a; EC, 2022b; UN Women, 2023

498	To provide targeted relief services, such as holiday care, for non-autonomous individuals to support caregivers	Social Welfare	All population	EC, 2022b
444	Rights of collective bargaining	Labor	All population	EC, 2022b
538	Age management in collective bargaining	Labor	Old age people	OECD, 2017b
491	To support the labour unions	Labor	All population	EC, 2022b
536	To enhance knowledge of worker rights	Labor	All population	EC, 2022b
539	To improve working conditions for those providing services to older people by empowering care workers with meaningful decision-making authority	Labor	All population	WHO, 2015a; WHO, 2015b
418	Support recognition of legal status of worker for informal caregivers	Labor	All population	EC, 2022b
482	Support for informal caregivers: raising the non-taxable income threshold	Labor	All population	EC, 2022b
483	Support of informal caregivers: increasing the ceiling on the number of hours in paid work outside the home	Labor	All population	EC, 2022b
308	Mobility policies: car-sharing, free public transport for commuting to work, and accessible transportation to healthcare facilities or workplaces	Domestic commerce	Old age people	WHO, 2019a; EC, 2021a
269	Individual and relationship-level interventions target those at risk of loneliness and social isolation, such as cognitive behavioural therapy to address maladaptive social cognition, which shows promise in reducing feelings of loneliness	Health	Old age people	WHO, 2021a
134	To engage civil society and private sector groups to support marginalised, excluded and vulnerable groups	Civil Rights	All population	UN, 2020
6	To enhance accessibility of public transport (including cross-border options and accommodations for disabled individuals) in both rural and urban areas to enable older people to fully participate in family, community life, and the workforce	Transportation	Old age people	WHO, 2015a; EC, 2018; WHO, 2019c
460	Strategies and programs related to ICTs should incorporate accessibility requirements for digital information, products, and services designed to reduce social isolation and loneliness among older adults	Technology	Old age people	WHO, 2021a
476	To support community activities for vulnerable older adults, including those living in nursing and care homes	Housing	Old age people	WHO, 2019b; EC, 2022b
459	Strategies and programs related to ICTs should provide appropriate digital knowledge and training to help older adults adopt new technologies	Technology	Old age people	WHO, 2012: WHO, 2021a
384	To safeguard the right to stay offline and offer alternatives for individuals who cannot or do not want to connect digitally	Civil Rights	Old age people	WHO, 2021a
452	Smart home solutions to prevent incidents and eliminate barriers to independence: fiscal support for adapted housing solutions and 'smart homes' with sensors and automated systems for electrical appliances, lighting and heating	Domestic commerce	Old age people	WHO, 2019a; EC, 2021a
546	Support flexible working time arrangements to combine work and caregiving for older workers: part-time working	Labor	All population	WHO, 2015a; WHO, 2015b; OECD, 2017a; OECD, 2017b; WHO, 2017b; WHO, 2019a; EC, 2021a; EC, 2022b; EC, 2022a; UN Women, 2023
547	To promote working arrangements that are enable a variety of employee normal working patterns in order to support a balance between work and family responsibilities: telework or ICT-mobile work	Labor	All population	UN Women, 2023

548	Campaign to promote the rights of individuals with disabilities and their caregivers, particularly labor rights pertaining to leave, flexible working arrangements (e.g., reduced hours, flexible hours, condensed hours), and part-time employment	Labor	All population	UN Women, 2023
549	To provide specific programs of employment for caregivers through employment services institutions	Labor	All population	UN Women, 2023
550	To establish a mobile service to visit and care for handicapped people during the day, whether a guardian or caregiver is employed	Health	All population	UN Women, 2023
552	Municipal multi-care facilities, such as those for healthy aging, childcare/kindergarten, and women's activity centers, should be promoted. Examples of activities include: skill transmission from older to younger women (such as sewing, knitting, weaving, cooking, art creation, and so on); knowledge transfer across generations - such as teaching fundamental IT and social media tools to older women; old age people who were doctors, teachers, etc., assisting others and/or teaching youngsters in the centers	Social Welfare	All population	UN Women, 2023
553	Advocacy/Media efforts to enhance knowledge of unpaid care work and gender stereotypes, employing local celebrities or artists that advocate for gender equality	Social Welfare	All population	UN Women, 2023
554	The establishment of multi-care centers at the municipal level should be based on the voluntary contributions of staff members who also use the facility, the symbolic fee paid by beneficiaries, the support and sponsorship of family members, the payment made by working women for cooking, sewing, and weaving classes, and the payment made by working women for child care and educational activities	Social Welfare	All population	UN Women, 2023
555	Helping elderly and disabled individuals exercise their rights (by completing the required paperwork and sending it to the appropriate organizations)	Civil Rights	All population	UN Women, 2023
556	To make plans to reduce digital inequality through e-governance, assisting underprivileged groups left out of traditional social protection programs and enabling seniors and people with disabilities to access digital services	Technology	Professionals/policy makers	UN, 2022
557	A more thorough approach places more emphasis on ability, affordability, and accessibility in order to assess and eliminate barriers to e-government. In addition to assessing the level of vulnerability and digital inclusion, these indicators can be used to direct proactive preventive actions and the development of targeted solutions. Developers have access to comprehensive data about digital access, affordability, and ability, which they may use to fill in some gaps in the design and implementation of e-government, increasing overall utilization rates and user satisfaction	Governmental Operations	Professionals/policy makers	UN, 2022
558	The integrated e-government architecture focuses on improving data, design, and delivery (enablers) to eliminate barriers related to ability, affordability, and access in order to guarantee that no one is left behind. This framework necessitates close coordination between departments, ministries, and affiliated organizations because digital projects typically have multiple facets	Governmental Operations	Professionals/policy makers	UN, 2022
559	A dynamic data-design-delivery framework that integrates the quickly evolving digital age tools and technology and promotes the development of well-thought-out and grounded e-government	Governmental Operations	Professionals/policy makers	UN, 2022
564	The integrated e-government architecture focuses on improving data, design, and delivery (enablers) to eliminate barriers related to ability, affordability, and access: monitoring, evaluation, and learning framework. This ensures that no one is left behind	Governmental Operations	Professionals/policy makers	UN, 2022
565	Ensuring that adequate financial, political and human resources to the e-governement goals	Governmental Operations	Professionals/policy makers	UN, 2022
566	Governments can streamline and optimize the use of IT resources by utilizing cloud technology (public, private, hybrid, and multi-vendor), which encourages the adoption of new digital technologies: Cloud technology offers	Governmental Operations	Professionals/policy makers	UN, 2022

567	To find the most effective ways to engage people, meet their needs, and assess the effects of digital services, governments should use data-driven, experimental, and AI-assisted data gathering techniques in addition to dynamic simulation models. New strategies that use systems thinking, foresight, pilot projects, and sandboxes to create and validate conceptual frameworks for innovative solutions should be created in order to take use of data-driven policy modeling	Governmental Operations	Professionals/policy makers	UN, 2022
568	Enacting comprehensive national legislation to combat the problem of elder abuse includes establishing standards for what behaviors are acceptable and unacceptable, protecting survivors legally, coordinating the response of all pertinent parties, and punishing those who engage in such abuse. In addition to taking into consideration the unique needs of older people as a non-homogeneous group—particularly the intersectional and cumulative factors that increase inequality and raise risks in older age—laws and policies pertaining to violence, abuse, and neglect must be in line with human rights, including the principles of equality and non-discrimination	Civil Rights	Professionals/policy makers	UNECE, 2022; UN, 2023
569	Adopting and implementing national action plans that enable states to consider the underlying causes of abuse and its effects on their elderly populations is one way to prevent elder abuse. These plans should include and define goals, priorities, assigned responsibilities, a schedule and evaluation system, and sufficient funding for implementation	Civil Rights	Professionals/policy makers	UN, 2023
571	Financial assistance for older adults fleeing violent conditions to live independently and in safe homes	Housing	Old age people	UN, 2023
572	Cooperation between social services, the criminal justice system, the health sector, and civil society to stop elder abuse: When older people ask for assistance leaving violent environments, develop a top-notch multisectoral response	Civil Rights	Professionals/policy makers	UN, 2023
573	Key players in avoiding old people abuse include police officers, prosecutors, attorneys, notaries, judges, psychosocial counselors, and financial community members who participate in training and capacity-building initiatives	Labor	Professionals/policy makers	UN, 2023
574	To guarantee older people who have experienced violence and abuse have access to legal aid and support: assistance with decision-making and legal support	Civil Rights	Old age people	UN, 2023
575	To make ageism a top political priority	Civil Rights	Professionals/policy makers	UN, 2023
576	To include senior citizens in the design and development of more age-friendly goods and services	Macroeconomics	Professionals/policy makers	UNECE, 2022
577	To encourage designers, companies, and public organizations to offer more intelligent digital, financial, and other services in order to foster innovation for the silver economy and value older people's continuous output and purchasing power as well as their participation to social and economic activities	Macroeconomics	Professionals/policy makers	UNECE, 2022

Strategy 2: Reduction of the burden of NCD

ID	Actions	Policy sectors	Target	Report
419	To reduce air pollution exposure among vulnerable groups, particularly older adults with pre-existing conditions and those from lower socioeconomic backgrounds, through education and communication initiatives	Environment	Old age people	WHO, 2019c
240	To improve health literacy among older adults by providing access to resources in age-friendly formats, while considering equity factors such as language barriers in the delivery of health information	Education	Old age people	WHO, 2019c

200	Free cessation treatments (e.g. smoking cessation medications, behavioural counselling, personalised feedback, physician advice, follow-up)	Health	All population	WHO, 2019a
194	For long-term care in communities and homes, professionals should inform caregivers of vulnerable older adults about the risks of heat waves and appropriate responses, providing recommendations for body cooling (e.g. applying ice towels, minimising clothing, using water-soaked cotton clothing, and employing fans with water applied to the body at air temperatures above 38°C, as older adults have lower sweating rates with advanced age)	Health	Professionals/policy makers	UN, 2022
500	To target assistance towards those most in need of a healthy diet	Health	Old age people	OECD, 2009
356	Preventive home visits for early detection of dementia, fall prevention, vaccinations, exercise promotion, and health literacy programs	Health	Old age people	OECD, 2009; WHO, 2015a; OECD, 2019a; EC, 2022a
358	Primary care services for older people should integrate health promotion, prevention, and disease management	Health	Old age people	OECD, 2009; WHO, 2021b
333	Personalised medicine for the prevention of cardiovascular diseases (CVD)	Health	All population	EC, 2022a
270	Infectious disease control programmes in institutions, extending beyond hospitals to take in other facilities, including those for older people	Health	Old age people	WHO, 2012
59	To implement cognitive brain-training programmes	Health	Old age people	WHO, 2019b
347	To plan an increase in the capacity of healthcare systems to identify and treat individuals with unhealthy diets and to enhance brief interventions	Health	All population	EC, 2022a
365	To promote and support multi-sectoral and intersectoral collaboration with diverse stakeholders (partnership) to design and evaluate actions to foster functional ability in ageing	Health	Old age people	WHO, 2015b; WHO, 2016; WHO, 2017b; WHO, 2020
95	To develop contingency plans for humanitarian emergencies that ensure an inclusive response for all ages. Emergency supplies, such as medical equipment, backup generators, food, and water, should be prepared and stockpiled for long-term care facilities, including healthcare centers. Older adults should be actively involved in developing policies, legislation, and programs, as well as in monitoring their implementation	Domestic commerce	Old age people	WHO, 2015a; UN, 2020; UN, 2022
348	To plan an increase in the capacity of health systems to identify and treat individuals with issues related to physical activity or inactivity, and to enhance brief interventions and physical activity prescriptions	Health	All population	WHO, 2019b; EC, 2022a
235	To develop implementing bodies (e.g., labor inspection agencies and occupational health services) that provide employers with guidelines, run information campaigns, and carry out preventive actions on workplace safety	Labor	All population	OECD, 2017b
343	To improve health information and raise awareness of NCDs and their risk factors among professionals in various sectors (e.g. healthcare, education, workplace)	Labor	All population	EC, 2022a
381	To promote complementary educational measures for managers on prevention of psychosocial risks and harassment at work	Labor	All population	EC, 2022a
241	To improve physical aspects in workplace	Labor	All population	EC, 2021a
422	To reduce exposure to hazardous substances and radiation	Labor	All population	EC, 2021b
492	To support mental health in the workplace	Labor	All population	EC, 2022b
427	Workplace safety regulations: Strictly enforce regulations to protect older workers from injuries	Labor	All population	OECD, 2017a; OECD, 2017b; WHO, 2019c;

				WHO, 2021b; EC, 2022b
120	To display distances in kilometers and travel times in minutes for car, public transportation, and walking	Transportation	All population	WHO, 2020
521	To establish goals for nutrition and involve older adults in group food preparation within community housing or meal arrangements	Health	Old age people	OECD, 2009
508	Technologies can enhance an older person’s safety and security at home. For instance, sensors and cameras can monitor the environment and analyse data to detect events such as falls, smoke alarm activations, or instances of wandering	Housing	Old age people	WHO, 2015a
320	To orient health systems around the intrinsic capacity and functional ability of older people: To adapt information systems for collecting, analysing, and reporting data on intrinsic capacity and trends in capacity. Data on intrinsic capacity and functional ability should be collected, analysed, and reported, disaggregated by age, sex, and other intersectional variables	Health	Old age people	WHO, 2015b; WHO, 2017b; WHO, 2021b
342	To plan improvements in health information to increase awareness of NCDs and their risk factors among professionals in various sectors (e.g. health, education, etc.)	Health	Professionals/policy makers	EC, 2022a
344	To plan improvements in health information to increase awareness of NCDs and their risk factors among the general population and vulnerable groups	Health	All population	EC, 2022a
416	To raise awareness of the benefits of an active and healthy lifestyle and to incentivize prevention over treatment	Health	All population	EC, 2018
230	To implement national immunization schedules, including for older age groups	Health	All population	WHO, 2012
56	To utilise communication policies, such as informative media campaigns, labelling requirements, and advertising restrictions, to shift consumer preferences away from harmful behaviours	Health	All population	OECD, 2019a
303	To implement a mass media campaign focused on prevention and health promotion	Health	All population	OECD, 2019a; EC, 2022b
490	To support the integrated development of tools and apps for data analytics that promote a healthy and active lifestyle	Health	All population	EC, 2018
396	To provide information in formats such as large print, “easy read” and pictures that meet the needs of older people, enabling them to make free and informed decisions	Health	Old age people	WHO, 2015b;WHO, 2017b
174	EU Mobile App for Cancer Prevention	Health	All population	EC, 2021b
345	To plan to improve screening for high-risk diabetes, CVD, and frailty	Health	All population	WHO, 2017c; EC, 2022a; EC, 2022b
357	To implement preventive and brief interventions for NCDs	Health	All population	EC, 2022a
528	To promote vaccinations for cancer prevention	Health	All population	EC, 2021b
379	To promote the development of globally competitive products, including wearable technologies, functional foods, personalized nutrition, and preventive medicine	Foreign Trade	All population	EC, 2018
515	To tighten limits on the concentrations of specific pollutants in surface or groundwater that could contribute to cancer incidence, including through the consumption of fish and shellfish	Environment	All population	EC, 2021b
251	To improve urban settings by reducing air and noise pollution through active mobility initiatives (such as walking and cycling), green infrastructure, and tactical urbanism, which reorganises public space to prioritise pedestrians and cyclists over private motorised vehicles	Housing	All population	WHO, 2019c; EC, 2022a
504	To impose taxes on alcohol	Macroeconomics	All population	OECD, 2009; WHO, 2019a; OECD, 2017a; EC, 2021b; OECD, 2019a

428	To make regulations on the availability of alcohol: i.e. limited hours of alcohol sale	Health	All population	WHO, 2019a; WHO, 2019b; OECD, 2019a; EC, 2022a
336	To place health warnings on alcohol content	Health	All population	WHO, 2019b
106	To provide a bundle of scientifically reliable information, based on non-industry-funded research, for the general public	Health	All population	EC, 2022a
107	To develop of a package of scientifically accurate information, based on research that is not industry-funded, for health professionals	Health	Professionals/policy makers	EC, 2022a
223	To identify and treat individuals at risk of/with harmful consumption of alcohol by enhancing screening and brief interventions, including pregnant women	Health	All population	EC, 2022a; EC, 2022b
289	To implement legislation and regulations to restrict alcohol advertising, with a focus on more effectively framing such advertising, particularly in digital and social media	Agriculture	All population	OECD, 2019a; WHO, 2019b; WHO, 2019a; EC, 2022a
484	To support prevention policies to avoid unhealthy behaviours; encourage behavioural changes, awareness benefits of active and healthy life-style	Health	All population	WHO, 2017b; EC, 2018; OECD, 2019a; OECD, 2019b; EC, 2021a; EC, 2022a
112	To provide nutritional guidance for the seniors to ensure that their meals are balanced. Dietary demands may fluctuate during the seniors's lifespan, necessitating recurring and adaptable counsel. Such advise should be designed in a way that allows the seniors to internalize the information	Health	Old age people	OECD, 2009
229	Implement a mass media campaign on healthy diets, including social marketing, to lower the intake of total fat, saturated fats, sugars, and salt, and encourage the intake of fruits and vegetables	Health	All population	EC, 2021b; EC, 2022a
231	To implement nutrition education and counselling in different settings (for example, in preschools, schools, workplaces and hospitals) to increase the intake of fruits and vegetables	Health	All population	WHO, 2015a; EC, 2022a
212	To promote healthy diet program	Health	All population	EC, 2022a
367	To promote functional foods and personalised nutrition and preventive medicine	Health	All population	OECD, 2017a; WHO, 2017b; EC, 2022a
290	To implement legislation and regulations to restrict the advertising of unhealthy foods	Agriculture	All population	OECD, 2019a
232	To implement nutrition labelling to reduce total energy intake (kcal), sugars, sodium and fats	Agriculture	All population	OECD, 2019a; WHO, 2019b; EC, 2022a
409	Provide water fountains in workplaces	Labor	All population	EC, 2022a
326	To create partnerships to promote health across sectors, professionals, between public, private, and non-government agencies	Health	All population	OECD, 2009
101	To develop tax benefits to the promotion of active mobility	Macroeconomics	All population	EC, 2022a
369	To promote investment in active mobility infrastructures	Transportation	All population	EC, 2021b
502	To implement tax on sugar and soft drinks	Macroeconomics	All population	EC, 2021b
505	To impose taxes on unhealthy foods and other economic initiatives that boost the cost of healthy and sustainable food options	Macroeconomics	All population	WHO, 2019a; WHO, 2019b;

				WHO, 2019c; OECD, 2017a; EC, 2021b
217	To increase taxes on tobacco	Macroeconomics	All population	OECD, 2009; WHO, 2019a; EC, 2021b; EC, 2002a
503	To impose taxes on novel tobacco products	Macroeconomics	All population	WHO, 2019b; EC, 2021b
458	Stop subsidising tobacco farming through its common agricultural policy (EU tobacco control framework)	Macroeconomics	All population	EC, 2022a
295	To promote the limitation of portions and food package sizes	Health	All population	EC, 2022a
433	To replace trans-fats and saturated fats with unsaturated fats	Agriculture	All population	EC, 2022a
128	To encourage healthy nutrition through the provision of nutritionally balanced dishes in canteens / snack machines / institutions for the seniors or social services serving the seniors in a non-institutional environment	Labor	All population	OECD, 2009; EC, 2022a
429	Regulations on the availability of unhealthy foods	Domestic commerce	All population	WHO, 2019a
44	Ban all flavours in every tobacco product and strengthen the ban by prohibiting flavour accessories, additives (EU tobacco control framework)	Domestic commerce	All population	EC, 2022a
46	To ban the display of tobacco products at points of sale (EU tobacco control framework)	Domestic commerce	All population	EC, 2022a
462	Strengthen packaging rules by introducing mandatory plain standardised packaging with graphic health warnings covering 80% of the front and the back of all tobacco products and introducing pack inserts (EU tobacco control framework)	Domestic commerce	All population	WHO, 2019b; EC, 2022a
510	The advertising, promotion, and sponsorship (TAPS) ban should include tobacco brand names and corporate promotion (EU tobacco control framework)	Domestic commerce	All population	EC, 2022a; EC, 2021b
129	To encourage local availability of healthy and affordable food (in restaurants, etc.)	Domestic commerce	All population	OECD, 2009
48	Ban tobacco product filters and filters marketed for use with tobacco products (EU tobacco control framework)	Domestic commerce	All population	EC, 2022a
294	To limit cross-border purchasing of tobacco and related products by private individuals	Foreign Trade	All population	EC, 2022a
102	To develop tax benefits to promote physical activity	Macroeconomics	All population	EC, 2022a
362	To promote programmes or exercise supported by the advice of a health professional accompanied by written material or advice from a primary-care professional and/or exercise specialist / provide advice about physical activity in all health and social care settings for older people, specifically targeting sedentary people, with a focus on promoting moderate intensity physical activity (particularly walking) and providing ongoing support	Health	Old age people	OECD, 2009; WHO, 2012
535	To promote exercise of moderate intensity and involved frequent professional contacts, when the interventions were: home-based; unsupervised and informal; used walking as the promoted exercise.	Health	Old age people	OECD, 2009
219	To promote home-based physical activity interventions	Health	Old age people	WHO, 2015a
334	To promote physical activity program	Health	All population	OECD, 2019b; EC, 2022a
130	To encourage the creation of internal sports teams	Labor	All population	EC, 2022a
131	To encourage the practice of physical activity by providing access to dedicated areas on the premises or by facilitating access to dedicated external structures	Labor	All population	EC, 2022a
472	To support agreements between gyms and enterprises to promote physical activity in workplace setting	Labor	All population	EC, 2022a

250	To enhance urban settings by promoting physical activity (e.g., safe walking areas, parks, and cycle paths) and emotional well-being through active mobility (walking and cycling), green infrastructure, and tactical urbanism that reorganises public space to prioritise pedestrians and cyclists over private motorised vehicles	Housing	All population	WHO, 2012; EC, 2022a; UN, 2022f
464	To make strengthen the role of primary care physicians in addressing heavy alcohol consumption	Health	All population	OECD, 2019a
315	To promote new models of health promotion and disease prevention in older age, to ensure these strategies are evidence-based	Health	Old age people	WHO, 2016
487	To support smoking quite with benefits by job contracts	Labor	All population	EC, 2022a
70	To promote continue to provide data on vaccine-preventable diseases and vaccination coverage among older people in order to obtain a better understanding of disease epidemiology	Health	All population	WHO, 2012
533	To promotion weight control program	Health	All population	EC, 2022a
234	To implement subsidies to increase the intake of fruits and vegetables	Social Welfare	All population	EC, 2022a
499	To support purchasing of healthy foods in low-income population between targeted transfers	Domestic commerce	All population	OECD, 2017a
501	To promote tax relief for e-bike purchase	Domestic commerce	All population	EC, 2021a
94	To develop and implement targeted community programmes for physical activity among older people, including a combination of individual and group-based behaviour change approaches with support and follow-up	Health	Old age people	WHO, 2012ff
228	Implement evidence-based multicomponent falls prevention programs, including tailored exercise programs, physical therapy, and balance retraining. Increase access to preventive measures for high-risk groups of older adults, such as the use of assistive devices like hip protectors. Address environmental hazards through modifications and conduct medication reviews. Additionally, improve training and access to relevant information for informal caregivers in the community	Health	Old age people	WHO, 2012; WHO, 2016; WHO, 2017b; WHO, 2019b; OECD, 2019a
170	To establish programs to improve diet by implementing national regulations on prepared or processed foods	Agriculture	All population	OECD, 2009
341	To plan to effectively promote strategies such as shelf placement, promotional policies, packaging of private brands, and nudging in major distribution channels (supermarkets) to encourage consumers to choose healthier options	Domestic commerce	All population	EC, 2022a
77	To create cooperation and sharing of experience and good practice on effective measures to increase physical activity levels among older persons, in order to support their implementation and evaluation	Health	Old age people	WHO, 2012
96	To develop coordinated approaches to more effectively frame advertising of food high in fat, sugar or salt, in particular on the digital and social media areas	Agriculture	All population	EC, 2022a
83	To promote deliver community-based interventions to prevent functional decline and care dependency	Health	Old age people	WHO, 2015b; WHO, 2017b; WHO, 2019c
275	To promote interventions incorporating self-monitoring and regular contact with an exercise specialist in a centre-based environment that promotes activity of moderate intensity	Health	Old age people	OECD, 2009
317	To conduct a nutritional assessment of older individuals through anthropometric measurements, clinical biochemistry, and dietary evaluation	Health	Old age people	WHO, 2015a
322	To orient health systems around the intrinsic capacity and functional ability of older adults. Sustainably finance the programs, services, and system realignments needed to promote Healthy Ageing	Health	Old age people	WHO, 2015b; WHO, 2017b
85	To redact design/review and pilot-test evidence-based guidelines for population and/or opportunistic screening on diabetes, CVD and clinical risk factors, including, for example, the joint definition of ethical guidance, minimum standards, and packages for the training of healthcare professionals	Health	Professionals/policy makers	EC, 2022a

450	To set national targets for providing one balanced meal a day	Health	Old age people	OECD, 2009
10	To promote adapted physical activity for seniors and Behavioral Interventions for Muscle Preservation	Health	Old age people	WHO, 2019a; EC, 2018; OECD, 2017a; EC, 2021b; WHO, 2017b; EC, 2022a; WHO, 2020; WHO, 2017c; OECD, 2019a
93	To develop and implement guidelines for prevention	Health	Old age people	EC, 2022b
32	To align air quality standards with EU and WHO recommendations	Environment	All population	EC, 2021b
50	Reiterate the ban on smoking in public spaces	Health	All population	OECD, 2019a
385	To protect people from tobacco smoke by implementing new smoke-free spaces	Health	All population	OECD, 2009; EC, 2022a
169	To establish extended smoke-free areas	Labor	All population	EC, 2022a
201	Provide free smoke areas in workplaces	Labor	All population	EC, 2022a
133	To enforce existing regulations on tobacco availability to limit access	Macroeconomics	All population	WHO, 2019a; EC, 2022a; OECD, 2019a
132	Enforce a minimum number (per 100 000 people) of sobriety checkpoints	Law and Crime	All population	EC, 2022a
164	To ensure the implementation of legislation related to novel tobacco products and avoid legislative gaps in response to new forms of use	Domestic commerce	All population	EC, 2022a
497	To promote tailored advocacy on healthy ageing	Health	All population	WHO, 2015b; WHO, 2017b; WHO, 2021b; UN, 2020
53	To promote campaigns to raise awareness of ageism and knowledge on healthy ageing	Civil Rights	All population	WHO, 2015a; WHO, 2015b; WHO, 2017b; UN, 2020; EC, 2022a; UN 2022
339	Plan to address health literacy using a community-based approach: health education, teach people how to care for themselves and each other as they get older; educate and empower people on the services use; health information and media campaign (development of websites and brochures, and other publicity measures)f	Education	All population	WHO, 2015b; WHO, 2017b; OECD, 2017a; OECD, 2019a; OECD, 2019b; UN 2020; EC, 2021b; EC, 2022a
524	To promote use of digital tools to support health promotion, prevention, and management	Health	All population	EC, 2022a
249	To improve the availability of non-communicable disease (NCD) data for decision-makers by creating mechanisms that enable effective communication between researchers and policymakers. To establish more effective methods to bridge the gap between knowledge generation and its application, considering the policy context. To facilitate the creation of relevant and timely evidence, conduct pertinent research on ageing and health, and identify cost-effective health system interventions suitable for local settings. To enhance communication by synthesising and packaging research findings in a way that policymakers	Health	All population	WHO, 2015b; WHO, 2016; WHO, 2017b; EC, 2022a

	can easily use. To empower decision-makers to utilise this information by fostering a culture that values evidence and its application.			
530	Vertical integrated care: coordination between a wide range of services, including health promotion and disease prevention, screening, early detection and acute care, ongoing management of chronic conditions, rehabilitation and palliative care	Health	Old age people	OECD, 2009; WHO, 2015a; WHO, 2016; WHO, 2021b
473	To support an interconnected person-centred pathway in health systems that provides health promotion, disease prevention, screening and early detection, treatment, rehabilitation and support to quality of life for patients with chronic diseases; Ensure the establishment of formal mechanisms for ability-oriented, person centred integrated long term care, for example through case management, advance care planning and collaboration between paid and unpaid caregivers	Health	All population	WHO, 2015a; WHO, 2015b; WHO, 2016; WHO, 2017b; EC, 2018; UN, 2020; EC, 2021a; EC, 2021b; EC, 2022a; EC, 2022b
274	Integration of health promotion and disease prevention in the health system	Health	All population	EC, 2022a
332	To promote personalised medicine for cancer prevention	Health	All population	EC, 2021b
442	Facilitate return-to-work programmes for people with mental health problems; tackling stigma and stereotypes, preventing stress, burn-out and bullying; right to disconnect	Labor	All population	EC, 2022a
403	To provide bicycle garages	Labor	All population	EC, 2022a
447	To promote screening activities in workplaces	Health	All population	WHO, 2019a
293	To promote lifestyle management activities and guidelines in workplaces to improve health and health behaviours	Health	All population	WHO, 2019a; OECD, 2017a
454	To offer social skills training, psychoeducation for coping and understanding, cognitive behavioural therapy, mindfulness training, and psychopharmacology, including antidepressants	Health	Old age people	WHO, 2021a
117	To promote digital interventions include telephone befriending	Social Welfare	Old age people	WHO, 2021a

Strategy 3: Access to quality health and social care

ID	Actions	Policy sectors	Target	Report
215	Helplines to provide information to anonymous callers and referrals for actual/potential victims of age discrimination	Civil Rights	Old age people	WHO, 2015a
183	Expanding training from academic centres into primary-care settings and communities	Education	Old age people	WHO, 2015a
23	Advocacy for better services	Social Welfare	Old age people	WHO, 2017c
311	Multidisciplinary teams of professionals from various disciplines who cooperate to address and resolve cases of older people abuse	Civil Rights	Old age people	WHO, 2015a
200	Free cessation treatments (e.g. smoking cessation medications, behavioural counselling, personalised feedback, physician advice, follow-up)	Health	All population	WHO, 2019a
144	Safe transport options for access to basic services, food and healthcare services	Transportation	Old age people	WHO, 2015a
194	For long-term care in communities and homes, professionals should inform caregivers of vulnerable older adults about the risks of heat waves and appropriate responses, providing recommendations for	Health	Professionals/policy makers	UN, 2022

	body cooling (e.g. applying ice towels, minimising clothing, using water-soaked cotton clothing, and employing fans with water applied to the body at air temperatures above 38°C, as older adults have lower sweating rates with advanced age)			
532	Wearables and mobile health applications to data collection for self-monitoring of health status	Health	All population	WHO, 2015a
109	Development of an operational definition of “frailty”	Health	Old age people	WHO, 2017c
103	To develop the market for mHealth devices and services tailored for older adults (i,e, digital clinical records, automated reminders, prompts, and warnings integrated into clinical health-record systems to assist healthcare personnel in meeting quality standards, systematically documenting diagnostic test results, and recording the care provided)	Health	Old age people	Who, 2015a; EC, 2018; EC, 2021a; EC, 2022b
545	To provide clear and accessible information concerning health and social services available for older people	Health	Old age people	WHO, 2015a
310	Multidisciplinary team in health care: trained nurses or other health workers who may complement physicians in key functions (assessment, treatment management, self-management support and follow-up), general practitioners, social workers, community-based workers and geriatricians for consultation and support; pharmacists, dietitians, rehabilitation therapists and psychologists; lay health workers	Health	Old age people	WHO, 2015a
211	More general competencies in communication, teamwork, information technology and public health for health workers	Health	Professionals/policy makers	WHO, 2015a
401	To provide training and transfer of knowledge and guidance for initiatives to improve the quality of care provided in resource-constrained settings and health care systems in transition	Health	Professionals/policy makers	WHO, 2012
195	Formal personnel who provide long-term care should have opportunities to increase their knowledge, capacity and skills with regard to aspects of climate change that affect older people	Health	Professionals/policy makers	WHO, 2015a; UN, 2022
255	To incorporate active ageing training modules into medical and health curricula at all levels	Education	Professionals/policy makers	WHO, 2017b
185	To extend the existing staff’s roles in delivering care for older people	Health	Professionals/policy makers	WHO, 2015b
3	To develop a Ministry of Health or Social Services with primary responsibility for the entire long-term care system, rather than just a segment of it	Health	All population	WHO, 2015a
358	Primary care services for older people should integrate health promotion, prevention, and disease management	Health	Old age people	OECD, 2009; WHO, 2021b
179	To regulate, select, and integrate evidence-based medical, health, and social services to adequately support older adults at home, in the community, or in institutions	Health	Old age people	WHO, 2016
206	To guide research and innovation to ensure that public and private sector developers and providers (including health and care services, medical devices, and pharmaceuticals) meet the needs of older adults, including those with limited resources	Health	Professionals/policy makers	WHO, 2015b; WHO, 2017b
415	To develop quality management systems that identify critical care points, with a focus on optimising functional ability and well-being	Health	Old age people	WHO, 2016
119	To develop digital memory aids	Health	All population	WHO, 2019a
270	Infectious disease control programmes in institutions, extending beyond hospitals to take in other facilities, including those for older people	Health	Old age people	WHO, 2012
59	To implement cognitive brain-training programmes	Health	Old age people	WHO, 2019b
368	To promote international networks in each region focused on geriatric issues	Health	Professionals/policy makers	WHO, 2012
198	To foster international exchanges of information on best practices in the evaluation and promotion of continuous training in competencies for the health and social care of older people	Health	Professionals/policy makers	WHO, 2012

254	To include an assessment of eating habits during geriatric evaluations	Health	Old age people	OECD, 2009
209	To promote health monitoring through home visits (e.g. reviewing health status and assessing adherence to prescribed medications, and ensuring that individuals are capable of self-care after an episode in an institution, whether acute or long-term)	Health	All population	OECD, 2009; WHO, 2015a
140	To ensure the monitoring of long-term care quality, its impact on functional ability and well-being, and the continuous improvement of care based on outcome	Health	All population	WHO, 2015b; WHO, 2016; WHO, 2017b; UN, 2020; EC, 2022a; EC, 2022b
51	Better clinical research is urgently needed on the aetiology and treatments of key health conditions affecting older adults, including musculoskeletal and sensory impairments, cardiovascular diseases and risk factors such as hypertension and diabetes, mental disorders, dementia and cognitive decline, cancer, and geriatric syndromes such as frailty	Health	Old age people	WHO, 2016
18	To adopt and implement WHO guidelines on integrated care for older people	Health	Old age people	WHO, 2015b; WHO, 2019c
146	To guarantee that protocols and guidelines concerning the training, ongoing education, and supervision of care workers are strictly followed, enforced through the licensing or accreditation process for care providers and facilities	Labor	Professionals/policy makers	WHO, 2015a
167	To establish or expand geriatric education programs, develop national guidelines on geriatric education, and define standards for geriatric training	Education	Professionals/policy makers	WHO, 2012; WHO, 2015b
272	Informal carers who provide long-term care should have opportunities to increase their knowledge, capacity and skills with regard to aspects of climate change that affect older people	Social Welfare	All population	UN, 2022
397	To provide occupational health and safety policies and programmes applicable to older people and informal workers	Labor	All population	WHO, 2019c
391	To provide continuing education, supervision, and other forms of support for existing paid caregivers	Labor	All population	WHO, 2015b; WHO, 2017b; OECD, 2017b; WHO, 2019c; EC, 2021
453	To promote social prescribing, which connects patients to local non-clinical sources of support	Social Welfare	Old age people	WHO, 2021a
468	Strong community engagement to ensure linkage and integration between climate issue and primary health care	Health	Old age people	UN, 2022
163	To ensure the capacity of health-care facilities to protect and enhance individual and community health by implementing WHO's operational framework for building climate-resilient health systems, utilising guidance for climate-resilient and environmentally sustainable health-care facilities, along with the accompanying toolkit and checklists for assessing susceptibility to various climate-related hazards	Health	All population	UN, 2022
91	To develop and enforce compliance with accessibility standards in buildings, transportation, information and communication technologies, and other assistive technologies	Technology	Old age people	WHO, 2015b
529	To implement validated community-level tools for assessing an older person's intrinsic capacity and frailty, in order to establish personalised care goals	Health	All population	WHO, 2017c
213	Help desks within local communities or online	Health	All population	EC, 2022a
525	To use of geocoded maps to assess the accessibility of locations	Transportation	All population	WHO, 2020

408	To provide sustainably grown, local food for staff and residents while incorporating on-site biodiverse gardens	Agriculture	All population	UN, 2022
494	To support voluntary and mutually agreed technology transfer that includes services, innovations, knowledge and best practices	Technology	Professionals/policy makers	WHO, 2015b; WHO, 2017b
317	To conduct a nutritional assessment of older individuals through anthropometric measurements, clinical biochemistry, and dietary evaluation	Health	Old age people	WHO, 2015a
364	To promote and support 'age-friendly' tourism destinations and tourist packages including accessibility and m-Health (e.g. age-friendly hotels)	Domestic commerce	All population	EC, 2018
405	To provide essential health care at a nationally defined minimum level that meets the criteria of availability, accessibility, acceptability, and quality	Health	All population	WHO, 2015a
159	To ensure access to essential medicines, vaccines, and innovations by facilitating the early market entry of generic essential medicines	Health	All population	WHO, 2019b
160	To ensure access to essential medicines, vaccines, and innovations by promoting the use of generic medicines among both consumers and healthcare professionals	Health	Professionals/policy makers	WHO, 2019b
158	To ensure access to essential medicines, vaccines, and innovations aimed at optimising older people's intrinsic capacities and functional abilities, including strategies such as reducing prices for essential medicines	Health	Old age people	WHO, 2015b; WHO, 2017b; WHO, 2019b; UN, 2020; EC, 2021b; WHO, 2021b
300	To establish long-term care insurance, with individuals contributing proportionally to their income through payroll or pension contributions, ensuring coverage is extended to all, regardless of income level or the availability of alternative informal caregiving options	Health	All population	WHO, 2019a; EC, 2021a; EC, 2021b
210	Health system: to design systems to foster the self-management of older people	Health	Old age people	WHO, 2015a; WHO, 2015b; WHO, 2017b
199	To foster self-management of service access by offering peer support, training, information, and advice to both older people and their caregivers	Health	All population	WHO, 2015a; WHO, 2015b; WHO, 2016
277	To introduce or strengthen legislation that promotes non-discrimination, on the basis of aspects such as gender, in the provision of health services	Civil Rights	Old age people	WHO, 2019c
8	To acknowledge the right of older people with significant losses of capacity to appropriate care and support, and to enshrine this aspect in national legislation to ensure access to quality services, with special attention to poor and marginalised older individuals	Civil Rights	Old age people	WHO, 2016
276	To introduce or strengthen legislation that promotes non-discrimination, on the basis of aspects such as age, in the provision of health services	Civil Rights	Old age people	WHO, 2019c
79	Co-payment reductions or exemptions for long-term care	Health	All population	OECD, 2017a
407	To provide social assistance within the home and community for the poorest and oldest individuals (often overlapping categories) and for those who lack family support (for example, through targeted cash transfers delivered within family or community networks)	Social Welfare	Old age people	WHO, 2015a
520	A universal and comprehensive health system for long-term care, provided at home, in communities, or within institutions, enables older adults who depend on care to continue their daily lives. Universal access to healthcare and social services must be guaranteed, with financial protection mechanisms, including cost-sharing regulations to support low-income families, particularly older individuals. This includes affordable coverage for medicines and assistive devices. Implementing universal health coverage should focus on reducing out-of-pocket payments, extending coverage, and expanding the	Health	All population	WHO, 2012; WHO, 2015b; WHO, 2016; OECD, 2017b; OECD, 2019a; OECD, 2019b;

	necessary services for older people. Publicly funded, accessible health services, including healthcare and long-term care, are essential for supporting the well-being of older adults			WHO, 2019c; UN, 2020; WHO, 2021b
449	Services should be situated as close as possible to where older adults live, including delivering care in their homes and providing community-based support	Social Welfare	Old age people	WHO, 2015a; WHO, 2016; WHO, 2017b; WHO, 2019c; UN Women, 2023
273	Information about services should be available in different languages	Immigration	All population	EC, 2022a
207	Health Equity Audits	Health	All population	EC, 2022a
20	To address access to financial products for cancer survivors	Domestic commerce	All population	EC, 2021b
98	To develop national standards, guidelines, protocols and accreditation for provision of community social care (i.e. to promote human rights for older people and their caregivers)	Social Welfare	Old age people	UN, 2020
338	To plan and develop 'dementia-friendly' communities that support individuals living with dementia in leading full and independent lives to the greatest extent possible	Housing	All population	EC, 2022a; OECD, 2019a
180	To assess financial practices and services to ensure fairness towards long-term survivors	Social Welfare	Old age people	EC, 2021b
299	To cover long-term care costs through a comprehensive social protection system, ensuring that out-of-pocket expenses for recipients of home care remain low enough for them to afford other essential living expenses, such as food, housing, and heating	Health	All population	WHO, 2017b; OECD, 2017a; UN, 2020; WHO, 2020; EC, 2021a; EC, 2022b
161	To ensure an adequately trained workforce including ageing issues into health professionals' curricula	Health	Professionals/policy makers	WHO, 2015a; WHO, 2015b; WHO, 2016; WHO, 2017b; WHO, 2021b; EC, 2022a
123	Emergency shelter provided for victims of older people abuse	Civil Rights	Old age people	WHO, 2015a
509	Telehealth services for rural populations or remote monitoring of chronic patients (in rural areas) (cancer patients and survivors)	Health	Old age people	WHO, 2015a; OECD, 2017a; WHO, 2019c; OECD, 2019a; EC, 2021b
148	To provide telehealth services for rural populations, including remote monitoring for chronic patients, such as cancer patients and survivors	Health	All population	WHO, 2015b; WHO, 2017b
457	To steward the development of the infrastructure and support necessary to ensure that long-term care is included under universal health coverage	Health	All population	WHO, 2015a; WHO, 2015b; WHO, 2017b
19	To adopt staff training programs that comprehensively cover geriatrics and gerontology, enhancing capacity planning for the future workforce (training should equip staff to perform basic screenings to assess functioning, including vision, hearing, cognition, nutritional status, and oral health; to manage common health conditions in older adults, such as frailty, osteoporosis, and arthritis; to understand how	Health	Professionals/policy makers	WHO, 2012; WHO, 2015a; WHO, 2017a; OECD, 2019a; WHO, 2020; EC,

	depression, dementia, and harmful alcohol use manifest in older individuals; to identify signs of neglect or abuse)			2022b; WHO, 2022b
54	Care schemes for informal carers	Health	All population	EC, 2022b; EC, 2021a; OECD, 2017a
156	To ensure the development and implementation of national care standards, guidelines, protocols for person centred integrated long-term care provision	Health	Professionals/policy makers	WHO, 2015b
222	To identify access to long-term care as a public health priority and a human right, including essential aids such as hearing and vision devices, basic mobility equipment (e.g. canes and walkers), personal hygiene tools, and adjustable furniture or bedding solutions	Health	All population	WHO, 2015a; WHO, 2015b
291	To adopt a life course paradigm within the healthcare system	Health	All population	OECD, 2017a; OECD, 2017b; WHO, 2021b; WHO, 2022a; WHO, 2022b
42	Automated medication dispensers	Health	All population	WHO, 2019a
307	Mobile pharmacies	Health	All population	EC, 2022a
111	To develop new models of care facilities (small-scale living facilities, mixed-care facilities, e-village for people with dementia)	Health	All population	OECD, 2019a
306	Mobile healthcare units	Health	All population	EC, 2022a
523	To use of call centres to communicate efficiently and cost-effectively with older people or their caregivers about the availability of services and to deliver standardised advices	Health	Old age people	WHO, 2017c
537	To improve accessibility for older adults with mobility issues and reduced intrinsic capacity in healthcare settings by training providers to enhance communication with visually or hearing-impaired individuals	Health	Professionals/policy makers	WHO, 2019c
105	To develop digital patient records through the centralization of patient data, institutional platforms, data registries, and electronic health records, fostering an open data culture that ensures adequate, effective, and shared recording of medical information	Health	All population	OECD, 2015; EC, 2018; OECD, 2019a; EC, 2022b
527	To use ICT for primary-care coordination in self-management of the therapy	Health	All population	OECD, 2009
214	To enhance understanding of data protection and privacy, address related legislative issues, and support 'open' data records for patients benefit	Health	Professionals/policy makers	EC, 2018; EC, 2022a; EC, 2022b; OECD, 2015
346	To plan initiatives that enhance support for patient empowerment and self-management of diabetes, chronic respiratory diseases, and cardiovascular disease (CVD), incorporating digital tools as integrated components of the care pathway used by both healthcare professionals and patients	Health	All population	OECD, 2019a; EC, 2022a
76	Cooperation and integration of healthcare services with social enterprises and non-profit organisations	Health	All population	EC, 2021a; WHO, 2016
216	To ensure high-quality long-term care by providing tools and resources that enable caregivers to deliver good-quality integrated care	Health	All population	WHO, 2015a; OECD, 2019a; EC, 2021b
220	Horizontal integrated care: coordination among different service levels, health/social services, and across different setting and providers	Health	Professionals/policy makers	OECD, 2009; WHO, 2015a; WHO, 2016; OECD, 2015; WHO, 2021b

514	To develop integrated and personalised health and care services/pathway for older adults	Health	Old age people	WHO, 2017b; EC, 2018; UN, 2020; WHO, 2021b
530	Vertical integrated care: coordination between a wide range of services, including health promotion and disease prevention, screening, early detection and acute care, ongoing management of chronic conditions, rehabilitation and palliative care	Health	Old age people	OECD, 2009; WHO, 2015a; WHO, 2016; WHO, 2021b
274	Integration of health promotion and disease prevention in the health system	Health	All population	EC, 2022a
192	To focus on the management of comorbidities in care	Health	All population	EC, 2022a
4	To establish a national policy on long-term care that encompasses homes, communities, and institutions, while encouraging the testing of approaches to further develop home-, community-, and institution-based care systems	Health	All population	WHO, 2012; WHO, 2015a; WHO, 2017a; OECD, 2019a; WHO, 2020; EC, 2022b; WHO, 2022b
193	Follow-up to monitor the effectiveness of support for informal caregivers and the overall strategy in long-term care	Health	Professionals/policy makers	EC, 2022a; EC, 2022b
331	Personalised medicine for cancer diagnosis and treatment	Health	All population	EC, 2021b
414	To implement a basic package of support for home care and informal caregivers, including alternative options for day care	Social Welfare	All population	WHO, 2012
477	To support community welfare by providing a comprehensive approach to long-term care that engages both informal and formal caregivers (including men, younger individuals, and non-family members like older volunteers and peers), stimulating collaboration between the public and private sectors and involving all levels of government, civil society, and the nonprofit sector	Social Welfare	Old age people	WHO, 2017b; OECD, 2019a; WHO, 2019c; WHO, 2021b
467	To strengthen the role of primary care in coordinating care for dependent and frail older adults	Health	Old age people	OECD, 2019a; OECD, 2019b
383	To promote the shift of services to outpatient or primary care clinics, as well as home and palliative care, to reduce costs	Health	Professionals/policy makers	WHO, 2019b
432	To include the work dimension in remobilization measures and rehabilitation programs for individuals with long-term care needs	Health	All population	EC, 2022b
63	To develop a common definition of ‘quality’ in care	Health	Professionals/policy makers	EC, 2022b
309	More regular follow-up of chronically-ill patients	Health	All population	OECD, 2009
184	To exploit innovative technology to improve efficiency in care	Health	All population	EC, 2021a
319	To offer community-based service provision for older people in need of long-term care, including those with disabilities	Health	All population	WHO, 2019c; EC, 2021a
236	To implement updated evidence-based guidelines for healthcare professionals	Health	All population	EC, 2022a
380	To promote the training of formal caregivers in the use of new digital technologies for older adults, including coaching and monitoring to support their development	Labor	Professionals/policy makers	EC, 2018; UN, 2020
448	To promote self-assessment as a tool for older adults to evaluate their health and support healthy ageing	Labor	All population	OECD, 2017a
155	To ensure that immigrant care workers are equipped with the essential skills and knowledge to effectively meet the expectations of their roles	Immigration	Professionals/policy makers	WHO, 2015a

518	To train health and social care workers in function-centred and integrated care, emphasising relational skills (e.g. active listening to older adults), and equipping them to conduct Healthy Aging assessments and develop care plans, which are crucial for implementing older-person-centred and integrated care	Health	Professionals/policy makers	WHO, 2015a; WHO, 2017c
360	To establish national standards for the training of professional caregivers	Health	Professionals/policy makers	WHO, 2015b; WHO, 2017b
542	To provide training that supports career opportunities, aiming to attract and retain paid caregivers	Labor	All population	WHO, 2015a; WHO, 2015b
479	To support immigration of highly educated people	Immigration	All population	EC, 2021a
55	To implement disease-specific education programs for caregivers	Social Welfare	All population	WHO, 2019a; WHO, 2016
7	To establish accreditation mechanisms for care providers, both institutional and professional, and implement monitoring systems to protect the rights of care recipients	Health	Professionals/policy makers	WHO, 2015b; WHO, 2016; WHO, 2017b
373	To promote new workforce cadres (such as care coordinators, case managers, and community care workers)	Health	Professionals/policy makers	WHO, 2015a; WHO, 2015b; WHO, 2017b
430	To provide reinforcement equipment for health and care settings	Health	Professionals/policy makers	EC, 2022b
431	To reinforce the medical workforce by increasing the number of caregivers and creating local job opportunities	Health	Professionals/policy makers	EC, 2022b; WHO, 2017c
318	To offer attractive working conditions to address health staff shortages	Labor	Professionals/policy makers	EC, 2021a
218	To implement hiring subsidies for healthcare workers	Labor	Professionals/policy makers	OECD, 2017a
541	To increase career opportunities for those providing services to older people	Labor	All population	WHO, 2015a; WHO, 2015b
543	To improve remuneration for those providing services to older people by increasing benefits	Labor	All population	WHO, 2015a; WHO, 2015b
245	To improve working conditions for those providing services to older adults by ensuring appropriate workloads	Labor	All population	WHO, 2015a; WHO, 2015b; WHO, 2017b
540	Improve remuneration for those providing services to older people by increasing their wages	Labor	All population	WHO, 2015a; WHO, 2015b
252	To address the shortage of healthcare staff by promoting cross-border mobility of health professionals	Immigration	Professionals/policy makers	EC, 2021a
271	To refocus the informal care workforce on tasks best suited for informal caregivers, such as mental health and spiritual support, rather than traditional formal care tasks	Health	All population	WHO, 2022b
481	To support informal caregivers improving existing services (e.g. longer opening hours of day centres, provision of mobile services also during the night)	Social Welfare	All population	WHO, 2019c; EC, 2022b
498	To provide targeted relief services, such as holiday care, for non-autonomous individuals to support caregivers	Social Welfare	All population	EC, 2022b
539	To improve working conditions for those providing services to older people by empowering care workers with meaningful decision-making authority	Labor	All population	WHO, 2015a; WHO, 2015b
287	To support legalised migration	Immigration	All population	EC, 2021a

308	Mobility policies: car-sharing, free public transport for commuting to work, and accessible transportation to healthcare facilities or workplaces	Domestic commerce	Old age people	WHO, 2019a; EC, 2021a
454	To offer social skills training, psychoeducation for coping and understanding, cognitive behavioural therapy, mindfulness training, and psychopharmacology, including antidepressants	Health	Old age people	WHO, 2021a
114	To implement digital interventions, including messaging services	Social Welfare	Old age people	WHO, 2021a
354	Policymakers and all stakeholders, including the private sector, should ensure that information and communication technologies (ICTs) are available, affordable, and accessible to older adults	Technology	Old age people	WHO, 2021a; WHO, 2021b
460	Strategies and programs related to ICTs should incorporate accessibility requirements for digital information, products, and services designed to reduce social isolation and loneliness among older adults	Technology	Old age people	WHO, 2021a
476	To support community activities for vulnerable older adults, including those living in nursing and care homes	Housing	Old age people	WHO, 2019b; EC, 2022b
256	To increase access to innovative support models for older adults to combat social isolation, including tele-links to social service providers	Social Welfare	Old age people	WHO, 2012
459	Strategies and programs related to ICTs should provide appropriate digital knowledge and training to help older adults adopt new technologies	Technology	Old age people	WHO, 2012: WHO, 2021a
2	'Connector services' target individuals at risk of loneliness and social isolation through outreach efforts, such as door-to-door visits, guided conversations, and motivational interviews	Social Welfare	Old age people	WHO, 2021a
463	To strengthen research capacities and incentives for innovation by promoting multidisciplinary research that incorporates older adults in all stages, addressing their needs and preferences, including in quality of care research	Health	Old age people	WHO, 2015b; WHO, 2016; WHO, 2017b; WHO, 2017c; UN, 2020; EC, 2022a
552	Municipal multi-care facilities, such as those for healthy aging, childcare/kindergarten, and women's activity centers, should be promoted. Examples of activities include: skill transmission from older to younger women (such as sewing, knitting, weaving, cooking, art creation, and so on); knowledge transfer across generations - such as teaching fundamental IT and social media tools to older women; old age people who were doctors, teachers, etc., assisting others and/or teaching youngsters in the centers	Social Welfare	All population	UN Women, 2023
554	The establishment of multi-care centers at the municipal level should be based on the voluntary contributions of staff members who also use the facility, the symbolic fee paid by beneficiaries, the support and sponsorship of family members, the payment made by working women for cooking, sewing, and weaving classes, and the payment made by working women for child care and educational activities	Social Welfare	All population	UN Women, 2023
572	Cooperation between social services, the criminal justice system, the health sector, and civil society to stop elder abuse: When older people ask for assistance leaving violent environments, develop a top-notch multisectoral response	Civil Rights	Professionals/policy makers	UN, 2023
573	Key players in avoiding old people abuse include police officers, prosecutors, attorneys, notaries, judges, psychosocial counselors, and financial community members who participate in training and capacity-building initiatives	Labor	Professionals/policy makers	UN, 2023
574	To guarantee older people who have experienced violence and abuse have access to legal aid and support: assistance with decision-making and legal support	Civil Rights	Old age people	UN, 2023
577	To encourage designers, companies, and public organizations to offer more intelligent digital, financial, and other services in order to foster innovation for the silver economy and value older people's continuous output and purchasing power as well as their participation to social and economic activities	Macroeconomics	Professionals/policy makers	UNECE, 2022

Strategy 4: Support for a longer working life

ID	Actions	Policy sectors	Target	Report
5	To abolish age-discriminatory laws	Civil Rights	Old age people	WHO, 2015b; WHO, 2017b; OECD, 2017a; WHO, 2019c; UN, 2020; WHO, 2020
235	To develop implementing bodies (e.g., labor inspection agencies and occupational health services) that provide employers with guidelines, run information campaigns, and carry out preventive actions on workplace safety	Labor	All population	OECD, 2017b
423	To reduce negative stereotypes about caregivers' roles / social recognition of careworker (Providing opportunities for continuing education and career progression could further help change perceptions that caregiving is a dead-end job. Providing academic opportunities for caregivers and establishing a serious research agenda may also shift perceptions and the value placed on caregiving, as well as fostering the rigorous identification of effective practices and care models)	Health	Professionals/policy makers	WHO, 2015a
381	To promote complementary educational measures for managers on prevention of psychosocial risks and harassment at work	Labor	All population	EC, 2022a
241	To improve physical aspects in workplace	Labor	All population	EC, 2021a
422	To reduce exposure to hazardous substances and radiation	Labor	All population	EC, 2021b
397	To provide occupational health and safety policies and programmes applicable to older people and informal workers	Labor	All population	WHO, 2019c
492	To support mental health in the workplace	Labor	All population	EC, 2022b
427	Workplace safety regulations: Strictly enforce regulations to protect older workers from injuries	Labor	All population	OECD, 2017a; OECD, 2017b; WHO, 2019c; WHO, 2021b; EC, 2022b
472	To support agreements between gyms and enterprises to promote physical activity in workplace setting	Labor	All population	EC, 2022a
323	To provide a package of placement, training, and counseling measures targeted at (disadvantaged) older workers / making available in the community programs for retraining that are customized to the requirements of older workers and can enable them to work for longer	Labor	Old age people	WHO, 2015a; OECD, 2017a; WHO, 2019c; WHO, 2021b
382	Promoting formal labor market participation is the most efficient approach of boosting coverage through earning-based pension schemes	Labor	All population	OECD, 2017b
28	To promote age-discrimination legislation in life-long learning	Civil Rights	Old age people	WHO, 2015b; WHO, 2017b; UN, 2020; WHO, 2020
27	To implement age-discrimination legislation in employment	Labor	Old age people	WHO, 2015b; WHO, 2017b; OECD, 2017a;

				WHO, 2019c; UN, 2020; WHO, 2020
29	To implement age-discrimination legislation to ensure participation and access to labour benefits	Labor	Old age people	UN, 2020; WHO, 2017b; WHO, 2019c
40	To ensure the right to work beyond the pensionable age: ban on obligatory retirement when attaining the statutory retirement age, so that persons who can and desire to do so can opt to continue to work beyond the statutory retirement age or to progressively phase in their retirement	Labor	Old age people	WHO, 2015a; OECD, 2017a; OECD, 2019a; WHO, 2020; EC, 2021a
435	To promote the research on research on the connections between ageism, other forms of prejudice and discrimination, and older people abuse	Civil Rights	Old age people	WHO, 2022a
17	Adjusting career requirements	Labor	Old age people	EC, 2021a
71	Continuous learning for health workers, continual support and supervision	Health	Professionals/policy makers	WHO, 2015a; EC, 2021b
432	To include the work dimension in remobilization measures and rehabilitation programs for individuals with long-term care needs	Health	All population	EC, 2022b
90	To provide an interactive platform that connects people working on creating innovative solutions with older people who wish to assist and/or invest in company growth, exchange expertise with the younger generation, or participate in test-bed activities	Labor	All population	EC, 2018
87	To develop the long-term care workforce through training and task-shifting (also including men, younger people and non-family members such as older volunteers and peers)	Health	All population	WHO, 2015a; WHO, 2015b
151	To guarantee that older workers may share their skills with other colleagues	Labor	All population	WHO, 2015a
363	To promote age-friendly universities and further education with the goal of boosting the employability of older adults. Help higher education institutions strengthen their provision of higher education for older persons	Education	All population	EC, 2018
189	Fiscal incentives for recruiting older workers (changing employers' perceptions toward the worth and contributions of older workers)	Macroeconomics	Old age people	WHO, 2015a; WHO, 2019c; EC, 2021a
259	To boost employment of persons with disabilities, which needs appropriate accommodations and an accessible work environment	Civil Rights	All population	EC, 2021a
301	Long-term leave and reduced working hours (mixed income from paid employment and pensions; national or sector-level partial retirement programs based on flexible/partial retirement, maybe with changing roles within the same organization). Flexibility in retirement systems that allow "the postponement of take-up of pensions" and "the receipt of pension income to be combined with work"	Labor	Old age people	OECD, 2017a; OECD, 2017b; EC, 2018; WHO, 2019a; WHO, 2019c; UN, 2020; WHO, 2021b; WHO, 2022
370	To increase mobility in the labor market of older workers, counseling, skills assessments, and job search support are needed early on, ideally within the notice period before individuals become jobless	Labor	All population	OECD, 2017a; OECD, 2017b
470	To map national employment and social protection programs by researching cancer survivors' return to work	Labor	All population	EC, 2021b
266	To increase the minimum retirement age or the required periods of contribution	Labor	Old age people	OECD, 2009
47	To ban the formal early retirement schemes	Labor	Old age people	OECD, 2009

442	Facilitate return-to-work programmes for people with mental health problems; tackling stigma and stereotypes, preventing stress, burn-out and bullying; right to disconnect	Labor	All population	EC, 2022a
21	Adult education and training in support of longer-term employment and for jobless persons	Labor	All population	EC, 2018; OECD, 2017a; EC, 2021a; OECD, 2019a; OECD, 2019b
22	Adult education and training to help "return" to work following a sick leave, as well as for jobless persons with impairments	Labor	All population	EC, 2021b
292	Lifelong learning programs to develop skills for doing non-routine and non-manual jobs for older workers: Provide chances for older individuals to learn new skills, especially in areas like information technology and new farming practices	Labor	Old age people	WHO, 2015b; WHO, 2017b; OECD, 2017a; OECD, 2017b; EC, 2018; OECD, 2019a; OECD, 2019b; WHO, 2019a; WHO, 2019c; UN, 2020; EC, 2021a; WHO, 2021b; WHO, 2021b; EC, 2022b
485	Support cancer survivors' reintegration into the workplace through re-skilling and up-skilling programs	Labor	All population	EC, 2021b
205	Guidance for funding, legal, prototype, and incubator services; European networking and EU-STARTUP projects; Work sessions, gathering experts and entrepreneurial stakeholders (incubators/accelerators, chambers of commerce, industrial parks)	Macroeconomics	All population	EC, 2018
430	To provide reinforcement equipment for health and care settings	Health	Professionals/policy makers	EC, 2022b
431	To reinforce the medical workforce by increasing the number of caregivers and creating local job opportunities	Health	Professionals/policy makers	EC, 2022b; WHO, 2017c
11	Adapting work methods to workers' ageing, integrating social partners such as trade unions, nonprofit groups, and employer organizations: flexible working practices	Labor	Old age people	WHO, 2015a; Who, 2015b; WHO, 2017b; OECD, 2019a; WHO, 2019a; WHO, 2019c
12	Adapting work methods to workers' ageing, integrating social partners such as trade unions, nonprofit groups, and employer organizations: job-sharing	Labor	Old age people	WHO, 2019a; WHO, 2019c
13	Adapting work practices to workers' aging and/or balancing work and family responsibilities with social partners such as trade unions, nonprofit groups, and employer organizations: part-time job	Labor	Old age people	WHO, 2019a; WHO, 2019c
14	Adapting work methods to workers' ageing, integrating social partners such as trade unions, nonprofit groups, and employer organizations: working from home / telework	Labor	Old age people	WHO, 2019a; WHO, 2019c; EC, 2021a
284	To set labor standards for preventing abuse in the workforce	Labor	All population	EC, 2022b
426	To regulate of working time	Labor	All population	OECD, 2017a

371	To encourage mobility in the employment market for older workers: Reliable processes are required to test and certify people's abilities and competences, make skills available to employers, and set a baseline for future learning. In the event of a job loss, this might assist employees find a suitable position	Labor	All population	OECD, 2017b
258	To encourage and support the labor engagement of persons living with diabetes or CVD	Labor	All population	EC, 2022a
411	To provide space-sharing facilities	Labor	All population	EC, 2018
440	Retaining older entrepreneurs completely inside the labor market by removing disincentives in tax	Macroeconomics	Old age people	EC, 2021a
441	Retaining older entrepreneurs completely inside the labor market by raising knowledge about the drivers of success for 50+ entrepreneurs	Macroeconomics	Old age people	EC, 2018
439	Retaining older entrepreneurs completely inside the labor market by removing disincentives in social security system	Labor	Old age people	EC, 2021a
534	To ensure well-designed sickness programs for workers	Labor	All population	OECD, 2017a; OECD, 2017b; EC, 2021b
191	To promote working arrangements that are enable a variety of employee normal working patterns in order to support a balance between work and family responsibilities: flextime	Labor	All population	WHO, 2015a; WHO, 2015b; OECD, 2017a; OECD, 2017b; WHO, 2017b; WHO, 2019a; EC, 2021a; EC, 2022b; EC, 2022a; UN Women, 2023
124	To promote the employment of older people	Labor	Old age people	WHO, 2015a; EC, 2021a
444	Rights of collective bargaining	Labor	All population	EC, 2022b
538	Age management in collective bargaining	Labor	Old age people	OECD, 2017b
491	To support the labour unions	Labor	All population	EC, 2022b
536	To enhance knowledge of worker rights	Labor	All population	EC, 2022b
293	To promote lifestyle management activities and guidelines in workplaces to improve health and health behaviours	Health	All population	WHO, 2019a; OECD, 2017a
418	Support recognition of legal status of worker for informal caregivers	Labor	All population	EC, 2022b
483	Support of informal caregivers: increasing the ceiling on the number of hours in paid work outside the home	Labor	All population	EC, 2022b
438	Retaining older entrepreneurs completely inside the labour market by ensuring access to finance	Domestic commerce	Old age people	EC, 2021a
546	To promote working arrangements that are enable a variety of employee normal working patterns in order to support a balance between work and family responsibilities: part-time working	Labor	All population	WHO, 2015a; WHO, 2015b; OECD, 2017a; OECD, 2017b; WHO, 2017b; WHO, 2019a; EC, 2021a; EC, 2022b; EC, 2022a; UN Women, 2023

547	To promote working arrangements that are enable a variety of employee normal working patterns in order to support a balance between work and family responsibilities: telework or ICT-mobile work	Labor	All population	UN Women, 2023
549	To provide specific programs of employment for caregivers through employment services institutions	Labor	All population	UN Women, 2023
550	To establish a mobile service to visit and care for handicapped people during the day, whether a guardian or caregiver is employed	Health	All population	UN Women, 2023
577	To encourage designers, companies, and public organizations to offer more intelligent digital, financial, and other services in order to foster innovation for the silver economy and value older people's continuous output and purchasing power as well as their participation to social and economic activities	Macroeconomics	Professionals/policy makers	UNECE, 2022
263	To enhance men's engagement in unpaid jobs (such as domestic chores and family care) to minimize gender inequality in the workplace and education	Civil Rights	All population	EC, 2021a; EC, 2022b

Strategy 5: Support permanence at home

ID	Actions	Policy sectors	Target	Report
149	Ensure that older people have information about housing options	Housing	Old age people	WHO, 2015a
237	To implement a policy that supports older adults' transition into more suitable housing	Housing	Old age people	WHO, 2015a
461	Ensure the strengthening of legal security of tenure for all the protection of citizens from illegal eviction (for older people)	Housing	All population	WHO, 2019c
471	To support "Housing First" policies to address later-life homelessness	Housing	Old age people	WHO, 2019c
233	To implement policies that support equitable access to quality and affordable housing for older people: social housing, assisted-living facilities, continuing-care communities and shared-living arrangements, including hostels and care homes	Housing	Old age people	WHO, 2015a; WHO, 2019c; WHO, 2021b
194	For long-term care in communities and homes, professionals should inform caregivers of vulnerable older adults about the risks of heat waves and appropriate responses, providing recommendations for body cooling (e.g. applying ice towels, minimising clothing, using water-soaked cotton clothing, and employing fans with water applied to the body at air temperatures above 38°C, as older adults have lower sweating rates with advanced age)	Health	Professionals/policy makers	UN, 2022
532	Wearables and mobile health applications to data collection for self-monitoring of health status	Health	All population	WHO, 2015a
179	To regulate, select, and integrate evidence-based medical, health, and social services to adequately support older adults at home, in the community, or in institutions	Health	Old age people	WHO, 2016
507	To develop technological and social innovations for home- and community-based services for older populations	Health	Old age people	WHO, 2015a; WHO, 2017b; EC, 2022b
209	To promote health monitoring through home visits (e.g. reviewing health status and assessing adherence to prescribed medications, and ensuring that individuals are capable of self-care after an episode in an institution, whether acute or long-term)	Health	All population	OECD, 2009; WHO, 2015a
423	To reduce negative stereotypes about caregivers' roles / social recognition of careworker (Providing opportunities for continuing education and career progression could further help change perceptions that caregiving is a dead-end job. Providing academic opportunities for caregivers and establishing a serious research agenda may also shift perceptions and the value placed on caregiving, as well as fostering the rigorous identification of effective practices and care models)	Health	Professionals/policy makers	WHO, 2015a

272	Informal carers who provide long-term care should have opportunities to increase their knowledge, capacity and skills with regard to aspects of climate change that affect older people	Social Welfare	All population	UN, 2022
187	To implement financial aid for informal carers of seniors	Social Welfare	All population	UN, 2022
397	To provide occupational health and safety policies and programmes applicable to older people and informal workers	Labor	All population	WHO, 2019c
391	To provide continuing education, supervision, and other forms of support for existing paid caregivers	Labor	All population	WHO, 2015b; WHO, 2017b; OECD, 2017b; WHO, 2019c; EC, 2021
84	Design housing that facilitates community integration	Housing	All population	WHO, 2015a
376	To promote sanitation, heating, and fuel efficiency in housing, while supporting home repair, maintenance, assessment, and modification through measures such as minimum housing standards	Housing	All population	WHO, 2019c
446	To develop schemes to maintain housing in good condition: cleaning and repair services	Housing	Old age people	WHO, 2015a
64	To support communication activities about adaptable and smart home solutions towards users and suppliers	Housing	Old age people	EC, 2018
508	Technologies can enhance an older person's safety and security at home. For instance, sensors and cameras can monitor the environment and analyse data to detect events such as falls, smoke alarm activations, or instances of wandering	Housing	Old age people	WHO, 2015a
181	To expand housing options and assist with home modifications that allow older adults to age in a place suited to their needs without financial burden	Social Welfare	Old age people	WHO, 2015b; WHO, 2017b
298	Loans, grants, or direct transfers (subsidies) for home modifications aimed at enhancing physical accessibility, comfort, and safety should be provided directly to older adults or to landlords whose older residents meet specific criteria, such as income level. Landlords must agree not to raise an older person's rent as a result of these adaptations	Housing	Old age people	WHO, 2015a
535	To promote exercise of moderate intensity and involved frequent professional contacts, when the interventions were: home-based; unsupervised and informal; used walking as the promoted exercise	Health	Old age people	OECD, 2009
228	Implement evidence-based multicomponent falls prevention programs, including tailored exercise programs, physical therapy, and balance retraining. Increase access to preventive measures for high-risk groups of older adults, such as the use of assistive devices like hip protectors. Address environmental hazards through modifications and conduct medication reviews. Additionally, improve training and access to relevant information for informal caregivers in the community	Health	Old age people	WHO, 2012; WHO, 2016; WHO, 2017b; WHO, 2019b; OECD, 2019a
300	To establish long-term care insurance, with individuals contributing proportionally to their income through payroll or pension contributions, ensuring coverage is extended to all, regardless of income level or the availability of alternative informal caregiving options	Health	All population	WHO, 2019a; EC, 2021a; EC, 2021b
210	Health system: to design systems to foster the self-management of older people	Health	Old age people	WHO, 2015a; WHO, 2015b; WHO, 2017b
199	To foster self-management of service access by offering peer support, training, information, and advice to both older people and their caregivers	Health	All population	WHO, 2015a; WHO, 2015b; WHO, 2016

407	To provide social assistance within the home and community for the poorest and oldest individuals (often overlapping categories) and for those who lack family support (for example, through targeted cash transfers delivered within family or community networks)	Social Welfare	Old age people	WHO, 2015a
98	To develop national standards, guidelines, protocols and accreditation for provision of community social care (i.e. to promote human rights for older people and their caregivers)	Social Welfare	Old age people	UN, 2020
338	To plan and develop 'dementia-friendly' communities that support individuals living with dementia in leading full and independent lives to the greatest extent possible	Housing	All population	EC, 2022a; OECD, 2019a
299	To cover long-term care costs through a comprehensive social protection system, ensuring that out-of-pocket expenses for recipients of home care remain low enough for them to afford other essential living expenses, such as food, housing, and heating	Health	All population	WHO, 2017b; OECD, 2017a; UN, 2020; WHO, 2020; EC, 2021a; EC, 2022b
222	To identify access to long-term care as a public health priority and a human right, including essential aids such as hearing and vision devices, basic mobility equipment (e.g. canes and walkers), personal hygiene tools, and adjustable furniture or bedding solutions	Health	All population	WHO, 2015a; WHO, 2015b
523	To use of call centres to communicate efficiently and cost-effectively with older people or their caregivers about the availability of services and to deliver standardised advices	Health	Old age people	WHO, 2017c
527	To use ICT for primary-care coordination in self-management of the therapy	Health	All population	OECD, 2009
346	To plan initiatives that enhance support for patient empowerment and self-management of diabetes, chronic respiratory diseases, and cardiovascular disease (CVD), incorporating digital tools as integrated components of the care pathway used by both healthcare professionals and patients	Health	All population	OECD, 2019a; EC, 2022a
473	To support an interconnected person-centred pathway in health systems that provides health promotion, disease prevention, screening and early detection, treatment, rehabilitation and support to quality of life for patients with chronic diseases; Ensure the establishment of formal mechanisms for ability-oriented, person centred integrated long-term care, for example through case management, advance care planning and collaboration between paid and unpaid caregivers	Health	All population	WHO, 2015a; WHO, 2015b; WHO, 2016; WHO, 2017b; EC, 2018; UN, 2020; EC, 2021a; EC, 2021b; EC, 2022a; EC, 2022b
414	To implement a basic package of support for home care and informal caregivers, including alternative options for day care	Social Welfare	All population	WHO, 2012
477	To support community welfare by providing a comprehensive approach to long-term care that engages both informal and formal caregivers (including men, younger individuals, and non-family members like older volunteers and peers), stimulating collaboration between the public and private sectors and involving all levels of government, civil society, and the non-profit sector	Social Welfare	Old age people	WHO, 2017b; OECD, 2019a; WHO, 2019c; WHO, 2021b
383	To promote the shift of services to outpatient or primary care clinics, as well as home and palliative care, to reduce costs	Health	Professionals/policy makers	WHO, 2019b
283	To involve older people themselves in service planning	Health	Old age people	WHO, 2016
380	To promote the training of formal caregivers in the use of new digital technologies for older adults, including coaching and monitoring to support their development	Labor	Professionals/policy makers	EC, 2018; UN, 2020
7	To establish accreditation mechanisms for care providers, both institutional and professional, and implement monitoring systems to protect the rights of care recipients	Health	Professionals/policy makers	WHO, 2015b; WHO, 2016; WHO, 2017b
271	To refocus the informal care workforce on tasks best suited for informal caregivers, such as mental health and spiritual support, rather than traditional formal care tasks	Health	All population	WHO, 2022b

191	Allow flexible working time arrangements to combine work and caregiving for older workers: flex time	Labor	All population	WHO, 2015a; WHO, 2015b; OECD, 2017a; OECD, 2017b; WHO, 2017b; WHO, 2019a; EC, 2021a; EC, 2022b; EC, 2022a; UN Women, 2023
498	To provide targeted relief services, such as holiday care, for non-autonomous individuals to support caregivers	Social Welfare	All population	EC, 2022b
539	To improve working conditions for those providing services to older people by empowering care workers with meaningful decision-making authority	Labor	All population	WHO, 2015a; WHO, 2015b
418	Support recognition of legal status of worker for informal caregivers	Labor	All population	EC, 2022b
483	Support of informal caregivers: increasing the ceiling on the number of hours in paid work outside the home	Labor	All population	EC, 2022b
512	The design of housing (e.g. communal areas) should reduce social isolation and loneliness	Housing	Old age people	WHO, 2021a
452	Smart home solutions to prevent incidents and eliminate barriers to independence: fiscal support for adapted housing solutions and ‘smart homes’ with sensors and automated systems for electrical appliances, lighting and heating	Domestic commerce	Old age people	WHO, 2019a; EC, 2021a
546	Support flexible working time arrangements to combine work and caregiving for older workers: part-time working	Labor	All population	WHO, 2015a; WHO, 2015b; OECD, 2017a; OECD, 2017b; WHO, 2017b; WHO, 2019a; EC, 2021a; EC, 2022b; EC, 2022a; UN Women, 2023
547	Promote working arrangements that are enable a variety of employee normal working patterns in order to support a balance between work and family responsibilities: telework or ICT-mobile work	Labor	All population	UN Women, 2023
571	Financial assistance for older adults fleeing violent conditions to live independently and in safe homes	Housing	Old age people	UN, 2023

Strategy 6: Social and cultural engagement

ID	Actions	Policy sectors	Target	Report
99	To develop partnerships for change involving older adults, their families, caregivers, and communities	Health	All population	UN, 2020
152	To ensure that public transport offers adequate stops and stations, allowing older adults to access senior centres, religious buildings, and visit their families	Transportation	Old age people	WHO, 2015a
378	To promote the civic engagement of older adults and strengthen volunteering and peer programs by providing information on volunteer opportunities	Social Welfare	Old age people	OECD, 2009; WHO, 2015a;

				WHO, 2012; WHO, 2019c
84	To design housing that facilitates community integration	Housing	All population	WHO, 2015a
521	To establish goals for nutrition and involve older adults in group food preparation within community housing or meal arrangements	Health	Old age people	OECD, 2009
372	To promote networks among stakeholders to foster ‘age-friendly’ cities and communities: Multi-interest participatory platforms (ensuring adequate representation will allow smaller settlements, such as towns and rural communities, to be included in national discussions, and provide a voice for underrepresented groups, including ethnic minorities, people with disabilities, indigenous populations, and those in low-income neighbourhoods)	Housing	All population	WHO, 2023
108	To develop a training package for general population to ensure competencies in ageing	Health	All population	EC, 2022a
251	To improve urban settings by reducing air and noise pollution through active mobility initiatives (such as walking and cycling), green infrastructure, and tactical urbanism, which reorganises public space to prioritise pedestrians and cyclists over private motorised vehicles	Housing	All population	WHO, 2019c; EC, 2022a
250	To enhance urban settings by promoting physical activity (e.g., safe walking areas, parks, and cycle paths) and emotional well-being through active mobility (walking and cycling), green infrastructure, and tactical urbanism that reorganises public space to prioritise pedestrians and cyclists over private motorised vehicles	Housing	All population	WHO, 2012; EC, 2022a; UN, 2022
175	EU silver tourism roadmap	Culture	Old age people	EC, 2018
400	To provide social opportunities as well as accessible information on leisure and social activities	Culture	Old age people	WHO, 2015b; WHO, 2017b
25	To implement age-based free participation or discounts for older adults to support access to cultural events	Domestic commerce	Old age people	EC, 2018
364	To promote and support ‘age-friendly’ tourism destinations and tourist packages including accessibility and m-Health (e.g. age-friendly hotels)	Domestic commerce	All population	EC, 2018
26	To offer age-based free participation or discounts for older adults to promote access to tourism activities	Domestic commerce	Old age people	EC, 2018
480	To promote intergenerational learning through mentoring and experience sharing in schools, community activities, libraries, museums, cultural and sports associations, and religious institutions	Culture	All population	WHO, 2012; OECD, 2017a; UN, 2020; EC, 2021a
28	To promote age-discrimination legislation in life-long learning	Civil Rights	Old age people	WHO, 2015b; WHO, 2017b; UN, 2020; WHO, 2020
58	To co-design public health strategies and person-centred care models that prioritise the specific needs, preferences, and competencies of older adults	Health	All population	WHO, 2015a; EC, 2022a
267	To increase university supply	Education	All population	EC, 2018
113	To enhance digital connectivity among workers for the purpose of knowledge exchange and collaboration	Labor	All population	EC, 2018; EC, 2021a; WHO, 2020; WHO, 2017c
265	To increase social dialogue	Labor	All population	EC, 2022b
498	To provide targeted relief services, such as holiday care, for non-autonomous individuals to support caregivers	Social Welfare	All population	EC, 2022b
513	To design public spaces with features like good lighting, benches, and accessible public toilets to reduce social isolation and loneliness, urban planning must prioritise physical accessibility and comfort for older adults	Housing	Old age people	WHO, 2015a; WHO, 2021a; UN, 2022

454	To offer social skills training, psychoeducation for coping and understanding, cognitive behavioural therapy, mindfulness training, and psychopharmacology, including antidepressants	Health	Old age people	WHO, 2021a
269	Individual and relationship-level interventions target those at risk of loneliness and social isolation, such as cognitive behavioural therapy to address maladaptive social cognition, which shows promise in reducing feelings of loneliness	Health	Old age people	WHO, 2021a
134	To engage civil society and private sector groups to support marginalised, excluded and vulnerable groups	Civil Rights	All population	UN, 2020
512	The design of housing (e.g. communal areas) should reduce social isolation and loneliness	Housing	Old age people	WHO, 2021a
196	To foster intergenerational relations through positive media reporting and public image campaigns	Civil Rights	All population	WHO, 2012
474	To support and stimulate intergenerational dialogue and collaboration	Culture	All population	WHO, 2012; WHO, 2019c; UN, 2020
475	To support co-housing among multigenerational residents to foster intergenerational contact and create communities that encourage daily social interaction and interdependence between generations	Housing	All population	EC, 2021a; OECD, 2017a
6	To enhance accessibility of public transport (including cross-border options and accommodations for disabled individuals) in both rural and urban areas to enable older people to fully participate in family, community life, and the workforce	Transportation	Old age people	WHO, 2015a; EC, 2018; WHO, 2019c
389	To provide community spaces where older people can gather, such as senior centres and community centres	Housing	Old age people	WHO, 2015a; WHO, 2015b; WHO, 2017b; WHO, 2019c
116	Digital interventions should include social networking sites	Social Welfare	Old age people	WHO, 2021a
395	To provide information and opportunities for social activities to promote inclusion, participation, and reduce loneliness and social isolation, such as involvement in community organisations and charitable initiatives	Social Welfare	Old age people	WHO, 2015a; OECD, 2009; UN, 2020
460	Strategies and programs related to ICTs should incorporate accessibility requirements for digital information, products, and services designed to reduce social isolation and loneliness among older adults	Technology	Old age people	WHO, 2021a
476	To support community activities for vulnerable older adults, including those living in nursing and care homes	Housing	Old age people	WHO, 2019b; EC, 2022b
88	Develop a standardised international tools to measure social isolation and loneliness	Health	Professionals/policy makers	WHO, 2021a
115	Digital interventions should include online discussion groups and forums	Social Welfare	Old age people	WHO, 2021a
394	To provide information about leisure activities to promote inclusion, participation, and reduce loneliness and social isolation	Domestic commerce	Old age people	WHO, 2015a; UN, 2020
445	To encourage the robotics and gaming industries to prioritise the needs and interests of older adults, collaborating to develop enjoyable products that reduce loneliness, promote mental health	Technology	Old age people	EC, 2018
511	To design restaurants, shops, and cultural institutions (e.g. libraries, museums, etc.) with a focus on accessibility and inclusivity to help reduce social isolation and loneliness	Housing	Old age people	WHO, 2021a
398	To offer leisure opportunities to promote inclusion, participation, and reduce loneliness and social isolation	Domestic commerce	Old age people	UN, 2020
2	‘Connector services’ target individuals at risk of loneliness and social isolation through outreach efforts, such as door-to-door visits, guided conversations, and motivational interviews	Social Welfare	Old age people	WHO, 2021a
388	To provide community spaces where older people can gather, such as public parks	Housing	Old age people	WHO, 2015a; WHO, 2015b;

				WHO, 2017b; WHO, 2019c
1	'Befriending services' that provide supportive relationships, either in person or over the phone, typically through volunteers	Social Welfare	Old age people	WHO, 2021a
173	To estimate effectiveness, costs, and cost-effectiveness of interventions to reduce social isolation and loneliness	Health	Old age people	WHO, 2021a
281	To invest in older people through community groups, organisations focused on older individuals, and self-help groups	Social Welfare	Old age people	WHO, 2016
329	Peer support and social activity groups: to support voluntary organisations to enhance older people's access to social activities	Social Welfare	Old age people	WHO, 2015a; WHO, 2021a
489	To support the development of older people's organisations	Social Welfare	Old age people	WHO, 2015b; WHO, 2017b
282	Involve older people in political processes that impact their rights by engaging them in the planning, implementation, and evaluation of local health, social services, and recreation programs, as well as in the development of research agendas on active ageing (both as advisors and investigators). This includes fostering broader coalitions and ensuring the political incorporation of minority groups, as well as actively involving older adults in policies and laws that concern them	Civil Rights	Old age people	WHO, 2015a; WHO, 2016; WHO, 2017b; WHO, 2019c; WHO, 2021b; WHO, 2021b
325	Participation of patients and citizens	Civil Rights	All population	WHO, 2016; EC, 2022a
244	To improve the understanding of the prevalence, distribution, and trends of social isolation	Health	Old age people	WHO, 2021a
552	Municipal multi-care facilities, such as those for healthy aging, childcare/kindergarten, and women's activity centres, should be promoted. Examples of activities include: skill transmission from older to younger women (such as sewing, knitting, weaving, cooking, art creation, and so on); knowledge transfer across generations - such as teaching fundamental IT and social media tools to older women; old age people who were doctors, teachers, etc., assisting others and/or teaching youngsters in the centres	Social Welfare	All population	UN Women, 2023
554	The establishment of multi-care centres at the municipal level should be based on the voluntary contributions of staff members who also use the facility, the symbolic fee paid by beneficiaries, the support and sponsorship of family members, the payment made by working women for cooking, sewing, and weaving classes, and the payment made by working women for child care and educational activities	Social Welfare	All population	UN Women, 2023
576	To include senior citizens in the design and development of more age-friendly goods and services	Macroeconomics	Professionals/policy makers	UNECE, 2022

Strategy 7: Market regulation

ID	Actions	Policy sectors	Target	Report
504	To impose taxes on alcohol	Macroeconomics	All population	OECD, 2009; WHO, 2019a; OECD, 2017a; EC, 2021b; OECD, 2019a

428	To make regulations on the availability of alcohol: i.e. limited hours of alcohol sale	Health	All population	WHO, 2019a; WHO, 2019b; OECD, 2019a; EC, 2022a
336	To place health warnings on alcohol content	Health	All population	WHO, 2019b
289	To implement legislation and regulations to restrict alcohol advertising, with a focus on more effectively framing such advertising, particularly in digital and social media	Agriculture	All population	OECD, 2019a; WHO, 2019b; WHO, 2019a; EC, 2022a
290	To implement legislation and regulations to restrict the advertising of unhealthy foods	Agriculture	All population	OECD, 2019a
232	To implement nutrition labelling to reduce total energy intake (kcal), sugars, sodium and fats	Agriculture	All population	OECD, 2019a; WHO, 2019b; EC, 2022a
502	To impose taxes on sugar and soft drinks	Macroeconomics	All population	EC, 2021b
505	To impose taxes on unhealthy foods and other economic initiatives that boost the cost of healthy and sustainable food options	Macroeconomics	All population	WHO, 2019a; WHO, 2019b; WHO, 2019c; OECD, 2017a; EC, 2021b
217	To increase taxes on tobacco	Macroeconomics	All population	OECD, 2009; WHO, 2019a; EC, 2021b; EC, 2002a
503	To impose taxes on novel tobacco products	Macroeconomics	All population	WHO, 2019b; EC, 2021b
295	To promote the limitation of portions and food package sizes	Health	All population	EC, 2022a
433	To replace trans-fats and saturated fats with unsaturated fats	Agriculture	All population	EC, 2022a
128	To encourage healthy nutrition through the provision of nutritionally balanced dishes in canteens / snack machines / institutions for the seniors or social services serving the seniors in a non-institutional environment	Labor	All population	OECD, 2009; EC, 2022a
429	Regulations on the availability of unhealthy foods	Domestic commerce	All population	WHO, 2019a
44	Ban all flavours in every tobacco product and strengthen the ban by prohibiting flavour accessories, additives (EU tobacco control framework)	Domestic commerce	All population	EC, 2022a
46	To ban the display of tobacco products at points of sale (EU tobacco control framework)	Domestic commerce	All population	EC, 2022a
462	Strengthen packaging rules by introducing mandatory plain standardised packaging with graphic health warnings covering 80% of the front and the back of all tobacco products and introducing pack inserts (EU tobacco control framework)	Domestic commerce	All population	WHO, 2019b; EC, 2022a
510	The advertising, promotion, and sponsorship (TAPS) ban should include tobacco brand names and corporate promotion (EU tobacco control framework)	Domestic commerce	All population	EC, 2022a; EC, 2021b
129	To encourage local availability of healthy and affordable food (in restaurants, etc.)	Domestic commerce	All population	OECD, 2009
48	Ban tobacco product filters and filters marketed for use with tobacco products (EU tobacco control framework)	Domestic commerce	All population	EC, 2022a
294	To limit cross-border purchasing of tobacco and related products by private individuals	Foreign Trade	All population	EC, 2022a

170	To establish programs to improve diet by implementing national regulations on prepared or processed foods	Agriculture	All population	OECD, 2009
341	To plan to effectively promote strategies such as shelf placement, promotional policies, packaging of private brands, and nudging in major distribution channels (supermarkets) to encourage consumers to choose healthier options	Domestic commerce	All population	EC, 2022a
133	To enforce existing regulations on tobacco availability to limit access	Macroeconomics	All population	WHO, 2019a; EC, 2022a; OECD, 2019a
164	To ensure the implementation of legislation related to novel tobacco products and avoid legislative gaps in response to new forms of use	Domestic commerce	All population	EC, 2022a
75	To control health price levels	Health	All population	WHO, 2019b
159	To ensure access to essential medicines, vaccines, and innovations by facilitating the early market entry of generic essential medicines	Health	All population	WHO, 2019b
158	To ensure access to essential medicines, vaccines, and innovations aimed at optimising older people's intrinsic capacities and functional abilities, including strategies such as reducing prices for essential medicines	Health	Old age people	WHO, 2015b; WHO, 2017b; WHO, 2019b; UN, 2020; EC, 2021b; WHO, 2021b
42	Automated medication dispensers	Health	All population	WHO, 2019a
445	To encourage the robotics and gaming industries to prioritise the needs and interests of older adults, collaborating to develop enjoyable products that reduce loneliness, promote mental health	Technology	Old age people	EC, 2018

Strategy 8: Home and cities adaptation

**ID**	**Actions**	**Policy sectors**	**Target**	**Report**
24	To offer affordable and accessible public and specialised community transport	Transportation	All population	WHO, 2015a; WHO, 2019c; WHO, 2021b
304	To assess age-friendliness of communities and apply an equity lens	Health	Old age people	WHO, 2019c
375	To promote research on innovations that enhance age-friendly environments, particularly in the workplace	Health	Old age people	WHO, 2015b; WHO, 2017b
30	Age-friendly cities and communities must have comprehensive, inclusive plans for climate change preparedness, mitigation and adaptation	Housing	Old age people	UN, 2022
237	To implement a policy that supports older adults' transition into more suitable housing	Housing	Old age people	WHO, 2015a
377	To promote sanitation, heating and fuel efficiency in housing, and support home repair, maintenance, assessment and modification through measures such as subsidies	Domestic commerce	Old age people	WHO, 2019c; UN Women, 2023
366	To promote equity-enhancing built environments, including land-use patterns and neighbourhood designs that support physical activity for all	Housing	Old age people	WHO, 2019c
233	To implement policies that support equitable access to quality and affordable housing for older people: social housing, assisted-living facilities, continuing-care communities and shared-living arrangements, including hostels and care homes	Housing	Old age people	WHO, 2015a; WHO, 2019c; WHO, 2021b

434	To develop a National Plan for Ageing and Health in collaboration with all relevant stakeholders, national and regional plans to foster Healthy Ageing. These plans should establish clear lines of responsibility, along with mechanisms for coordination, accountability, monitoring, and reporting across all relevant sectors. A national programme should be developed to support activities aligned with the WHO Global Network for Age-friendly Cities and Communities. National frameworks should be established for action on Healthy Ageing that engage all sectors, different levels of government, and civil society (including associations for older people, families, and carers), while ensuring an age-friendly primary care approach	Housing	Old age people	WHO, 2015a; WHO, 2015b; WHO, 2016; WHO, 2017b; OECD, 2017b; WHO, 2019c; WHO, 2020; OECD, 2021b; EC, 2022b
144	Safe transport options for access to basic services, food and healthcare services	Transportation	Old age people	WHO, 2015a
507	To develop technological and social innovations for home- and community-based services for older populations	Health	Old age people	WHO, 2015a; WHO, 2017b; EC, 2022b
150	To ensure that older people are provided with priority seating	Transportation	Old age people	WHO, 2015a
152	To ensure that public transport offers adequate stops and stations, allowing older adults to access senior centres, religious buildings, and visit their families	Transportation	Old age people	WHO, 2015a
153	To ensure that structures and landscapes are accessible, safe, and thoughtfully designed	Housing	Old age people	WHO, 2015a
143	To ensure safe road conditions for both drivers and pedestrians crossing streets and provide priority parking for older adults with disabilities	Housing	Old age people	WHO, 2015a
84	To design housing that facilitates community integration	Housing	All population	WHO, 2015a
376	To promote sanitation, heating, and fuel efficiency in housing, while supporting home repair, maintenance, assessment, and modification through measures such as minimum housing standards	Housing	All population	WHO, 2019c
221	Housing must be accessible and well-designed, using materials that promote sustainable energy efficiency for cooling and insulation, while minimising energy demand for heating and cooling	Housing	Old age people	WHO, 2015a; WHO, 2021b;UN, 2022
508	Technologies can enhance an older person’s safety and security at home. For instance, sensors and cameras can monitor the environment and analyse data to detect events such as falls, smoke alarm activations, or instances of wandering	Housing	Old age people	WHO, 2015a
451	Smart home solutions (sensors and automated systems for electrical appliances, lighting and heating)	Housing	Old age people	EC, 2018; OECD, 2019a; EC, 2021a
181	To expand housing options and assist with home modifications that allow older adults to age in a place suited to their needs without financial burden	Social Welfare	Old age people	WHO, 2015b; WHO, 2017b
392	To provide early assessment of housing needs	Housing	Old age people	WHO, 2015a
335	To install water fountains in urban developments	Housing	All population	EC, 2022a
91	To develop and enforce compliance with accessibility standards in buildings, transportation, information and communication technologies, and other assistive technologies	Technology	Old age people	WHO, 2015b
359	Private and public buildings and institutions, including health and social care facilities, must be accessible and well-designed, utilising materials that promote sustainable energy efficiency for cooling and insulation while minimising energy demand for heating and cooling. Ensure the application of recommendations for climate-friendly hospitals and long-term care facilities by developing effective plans for heat reduction and management tailored to local conditions. This includes using rooftop sprinklers, outdoor sunshades to protect common areas, heat-reflective window glass, installing evaporative coolers, and ensuring adequate natural ventilation	Health	All population	UN, 2022

372	To promote networks among stakeholders to foster ‘age-friendly’ cities and communities: Multi-interest participatory platforms (ensuring adequate representation will allow smaller settlements, such as towns and rural communities, to be included in national discussions, and provide a voice for underrepresented groups, including ethnic minorities, people with disabilities, indigenous populations, and those in low-income neighbourhoods)	Housing	All population	WHO, 2023
374	To promote partnerships and networks among stakeholders for fostering age-friendly cities and communities, it is essential to engage a diverse range of participants, including older adults, families and caregivers, organisations representing the interests of older people (such as councils and commissions), organisations for people with disabilities, and groups advocating for patients and their families, particularly regarding non-communicable diseases. Additionally, include organisations representing marginalised groups, representatives from government agencies, community leaders (from urban, rural, remote, indigenous, ethnic minority, and displaced communities), relevant ministries (such as health, social welfare, local government, transport, housing, and finance), regional and local authorities, members of parliament, international organisations, professional associations, donors and philanthropists, academia and research groups, the media, and the private sector	Housing	All population	WHO, 2023
298	Loans, grants, or direct transfers (subsidies) for home modifications aimed at enhancing physical accessibility, comfort, and safety should be provided directly to older adults or to landlords whose older residents meet specific criteria, such as income level. Landlords must agree not to raise an older person's rent as a result of these adaptations	Housing	Old age people	WHO, 2015a
251	To improve urban settings by reducing air and noise pollution through active mobility initiatives (such as walking and cycling), green infrastructure, and tactical urbanism, which reorganises public space to prioritise pedestrians and cyclists over private motorised vehicles	Housing	All population	WHO, 2019c; EC, 2022a
369	To promote investment in active mobility infrastructures	Transportation	All population	EC, 2021b
100	To develop pedestrian infrastructure that uses universal design principles	Housing	All population	WHO, 2015a
250	To enhance urban settings by promoting physical activity (e.g., safe walking areas, parks, and cycle paths) and emotional well-being through active mobility (walking and cycling), green infrastructure, and tactical urbanism that reorganises public space to prioritise pedestrians and cyclists over private motorised vehicles	Housing	All population	WHO, 2012; EC, 2022a; UN, 2022
544	To ensure that housing is not overcrowded	Housing	Old age people	WHO, 2015a
313	National and subnational health plans should be reviewed and revised considering a variety of climate-related situations, such as prolonged, extreme heat waves, sudden floods or spikes in air pollution, opportunities to promote and support older people’s resilience to climate change	Health	Old age people	UN, 2022
228	Implement evidence-based multicomponent falls prevention programs, including tailored exercise programs, physical therapy, and balance retraining. Increase access to preventive measures for high-risk groups of older adults, such as the use of assistive devices like hip protectors. Address environmental hazards through modifications and conduct medication reviews. Additionally, improve training and access to relevant information for informal caregivers in the community	Health	Old age people	WHO, 2012; WHO, 2016; WHO, 2017b; WHO, 2019b; OECD, 2019a
34	To align strategies with the “green transition” by encouraging urban and city planners, architects, and engineers to identify climate-adaptive, nature-based solutions for green (vegetated) and blue (water) spaces. These solutions provide significant benefits for both people and biodiversity in cities and include increasing the number of parks, tree-lined streets, paths, urban gardens, green roofs, and building facades	Environment	All population	EC, 2022a; UN 2022
385	To protect people from tobacco smoke by implementing new smoke-free spaces	Health	All population	OECD, 2009; EC, 2022a
169	To establish extended smoke-free areas	Labor	All population	EC, 2022a

92	To develop and ensure gender-responsive, affordable, accessible, and sustainable mobility by adhering to accessibility standards in buildings and implementing safe transport systems, pavements, and roads	Transportation	All population	UN, 2020
449	Services should be situated as close as possible to where older adults live, including delivering care in their homes and providing community-based support	Social Welfare	Old age people	WHO, 2015a; WHO, 2016; WHO, 2017b; WHO, 2019c; UN Women, 2023
338	To plan and develop 'dementia-friendly' communities that support individuals living with dementia in leading full and independent lives to the greatest extent possible	Housing	All population	EC, 2022a; OECD, 2019a
81	To create age-friendly health infrastructures, including elevators, escalators, ramps, and doorways that are accessible for individuals with significant physical limitations or those using wheelchairs. Ensure that stairs are designed to be suitable (not too high or steep) with hand railings, incorporate non-slip flooring, provide rest areas with comfortable seating, and use large, clear, well-lit signs. Additionally, improve accessibility for older adults with mobility issues and reduced intrinsic capacity in healthcare settings by installing wheelchair ramps and providing ample seating	Health	All population	WHO, 2015a; WHO, 2019c
111	To develop new models of care facilities (small-scale living facilities, mixed-care facilities, e-village for people with dementia)	Health	All population	OECD, 2019a
262	To increase of ambulatory and community care infrastructures	Health	All population	EC, 2021a
243	To improve transport (shuttle buses, specialised driverless busses for the frailer) also ensuring connectivity across borders	Transportation	All population	EC, 2018; EC, 2021a
403	To provide bicycle garages	Labor	All population	EC, 2022a
513	To design public spaces with features like good lighting, benches, and accessible public toilets to reduce social isolation and loneliness, urban planning must prioritise physical accessibility and comfort for older adults	Housing	Old age people	WHO, 2015a; WHO, 2021a; UN, 2022
134	To engage civil society and private sector groups to support marginalised, excluded and vulnerable groups	Civil Rights	All population	UN, 2020
512	The design of housing (e.g. communal areas) should reduce social isolation and loneliness	Housing	Old age people	WHO, 2021a
6	To enhance accessibility of public transport (including cross-border options and accommodations for disabled individuals) in both rural and urban areas to enable older people to fully participate in family, community life, and the workforce	Transportation	Old age people	WHO, 2015a; EC, 2018; WHO, 2019c
389	To provide community spaces where older people can gather, such as senior centres and community centres	Housing	Old age people	WHO, 2015a; WHO, 2015b; WHO, 2017b; WHO, 2019c
511	To design restaurants, shops, and cultural institutions (e.g. libraries, museums, etc.) with a focus on accessibility and inclusivity to help reduce social isolation and loneliness	Housing	Old age people	WHO, 2021a
412	Public services, including transportation and safety services (police, fire), must be adaptive to the needs of older adults. Ensure that front-line transportation operators are trained to understand the needs of older individuals and to follow non-discriminatory practices	Transportation	Professionals/policy makers	WHO, 2015a; OECD, 2019a; WHO, 2021a
43	Automation and digitalisation of transports	Transportation	All population	EC, 2021a
388	To provide community spaces where older people can gather, such as public parks	Housing	Old age people	WHO, 2015a; WHO, 2015b;

				WHO, 2017b; WHO, 2019c
452	Smart home solutions to prevent incidents and eliminate barriers to independence: fiscal support for adapted housing solutions and 'smart homes' with sensors and automated systems for electrical appliances, lighting and heating	Domestic commerce	Old age people	WHO, 2019a; EC, 2021a

Strategy 9: Environmental interventions

ID	Actions	Policy sectors	Target	Report
30	Age-friendly cities and communities must have comprehensive, inclusive plans for climate change preparedness, mitigation and adaptation	Housing	Old age people	UN, 2022
377	To promote sanitation, heating and fuel efficiency in housing, and support home repair, maintenance, assessment and modification through measures such as subsidies	Domestic commerce	Old age people	WHO, 2019c; UN Women, 2023
419	To reduce air pollution exposure among vulnerable groups, particularly older adults with pre-existing conditions and those from lower socioeconomic backgrounds, through education and communication initiatives	Environment	Old age people	WHO, 2019c
194	For long-term care in communities and homes, professionals should inform caregivers of vulnerable older adults about the risks of heat waves and appropriate responses, providing recommendations for body cooling (e.g. applying ice towels, minimising clothing, using water-soaked cotton clothing, and employing fans with water applied to the body at air temperatures above 38°C, as older adults have lower sweating rates with advanced age)	Health	Professionals/policy makers	UN, 2022
272	Informal carers who provide long-term care should have opportunities to increase their knowledge, capacity and skills with regard to aspects of climate change that affect older people	Social Welfare	All population	UN, 2022
422	To reduce exposure to hazardous substances and radiation	Labor	All population	EC, 2021b
469	To develop strong intergenerational communication, collaboration and solidarity, including older people and supporting them in engaging in climate actions, rather than pitting generations against each other	Environment	All population	WHO, 2021b; UN, 2022
351	Policy and law and interventions for advocacy and communications could be guided by the reports of the Independent Expert on the enjoyment of all human rights by older persons and the evidence-based recommendations of the Global report on ageism (topic climate safety)	Environment	All population	WHO, 2021b; UN, 2022
468	Strong community engagement to ensure linkage and integration between climate issue and primary health care	Health	Old age people	UN, 2022
163	To ensure the capacity of health-care facilities to protect and enhance individual and community health by implementing WHO's operational framework for building climate-resilient health systems, utilising guidance for climate-resilient and environmentally sustainable health-care facilities, along with the accompanying toolkit and checklists for assessing susceptibility to various climate-related hazards	Health	All population	UN, 2022
203	Global, regional and national responses to climate change should ensure that attention is paid to older people and avoid institutional and interpersonal discrimination according to age	Environment	Old age people	UN, 2022
359	Private and public buildings and institutions, including health and social care facilities, must be accessible and well-designed, utilising materials that promote sustainable energy efficiency for cooling and insulation while minimising energy demand for heating and cooling. Ensure the application of recommendations for climate-friendly hospitals and long-term care facilities by developing effective plans for heat reduction and management tailored to local conditions. This includes using rooftop	Health	All population	UN, 2022

	sprinklers, outdoor sun shades to protect common areas, heat-reflective window glass, installing evaporative coolers, and ensuring adequate natural ventilation			
186	To finance green transition (green energy)	Energy	All population	WHO, 2021b; EC, 2022a
408	To provide sustainably grown, local food for staff and residents while incorporating on-site biodiverse gardens	Agriculture	All population	UN, 2022
69	To promote conserving and/or harvesting water and avoiding bottled water when safe alternatives exist	Environment	All population	UN, 2022
424	To promote the reduction, reuse, recycling, composting and use of alternatives to waste incineration	Environment	All population	UN, 2022
515	To tighten limits on the concentrations of specific pollutants in surface or groundwater that could contribute to cancer incidence, including through the consumption of fish and shellfish	Environment	All population	EC, 2021b
52	Promote building facilities adapted to local climatic conditions and optimized for reduced energy and resource demands	Energy	All population	UN, 2022
421	Promote the reduction of energy consumption and costs through efficiency and conservation measures	Energy	All population	UN, 2022
361	Promote the production and/or consumption of clean and renewable energy to ensure reliable, resilient operation	Energy	All population	UN, 2022
526	Promote the use of alternative fuels for vehicle fleets	Energy	All population	UN, 2022
251	To improve urban settings by reducing air and noise pollution through active mobility initiatives (such as walking and cycling), green infrastructure, and tactical urbanism, which reorganises public space to prioritise pedestrians and cyclists over private motorised vehicles	Housing	All population	WHO, 2019c; EC, 2022a
60	To promote collaboration beyond the environment and health sectors to encompass infrastructure, employment, education, social protection, housing, energy, and transport, particularly in cities with high concentrations of older people and in small island developing states	Government Operations	All population	WHO, 2017b; UN, 2022
312	Promote the multi-sectoral collaboration (including energy, technology (e.g., for air-conditioning), transport, water and sanitation and waste management, finance) to include healthy ageing in global climate strategies: health and social care providers should be actively engaged in making decisions on the climate crisis and leading	Government Operations	Old age people	UN, 2022
409	Provide water fountains in workplaces	Labor	All population	EC, 2022a
250	To enhance urban settings by promoting physical activity (e.g., safe walking areas, parks, and cycle paths) and emotional well-being through active mobility (walking and cycling), green infrastructure, and tactical urbanism that reorganises public space to prioritise pedestrians and cyclists over private motorised vehicles	Housing	All population	WHO, 2012; EC, 2022a; UN, 2022
313	National and subnational health plans should be reviewed and revised considering a variety of climate-related situations, such as prolonged, extreme heat waves, sudden floods or spikes in air pollution, opportunities to promote and support older people's resilience to climate change	Health	Old age people	UN, 2022
246	Improved monitoring, modelling and air quality plans will help local authorities to achieve cleaner air	Environment	All population	EC, 2021b
34	To align strategies with the "green transition" by encouraging urban and city planners, architects, and engineers to identify climate-adaptive, nature-based solutions for green (vegetated) and blue (water) spaces. These solutions provide significant benefits for both people and biodiversity in cities and include increasing the number of parks, tree-lined streets, paths, urban gardens, green roofs, and building facades	Environment	All population	EC, 2022a; UN 2022
32	To align air quality standards with EU and WHO recommendations	Environment	All population	EC, 2021b

Strategy 10: Communication, media, and advertising

ID	Actions	Policy sectors	Target	Report
215	Helplines to provide information to anonymous callers and referrals for actual/potential victims of age discrimination	Civil Rights	Old age people	WHO, 2015a
149	Ensure that older people have information about housing options	Housing	Old age people	WHO, 2015a
280	To promote investment on technologies and ICT (i.e. e-health and telemedicine)	Health	All population	WHO, 2021b
145	Ensure that captioning is available for TV broadcasts for those with hearing impairments.	Technology	All population	WHO, 2015a
419	To reduce air pollution exposure among vulnerable groups, particularly older adults with pre-existing conditions and those from lower socioeconomic backgrounds, through education and communication initiatives	Environment	Old age people	WHO, 2019c
240	To improve health literacy among older adults by providing access to resources in age-friendly formats, while considering equity factors such as language barriers in the delivery of health information	Education	Old age people	WHO, 2019c
194	For long-term care in communities and homes, professionals should inform caregivers of vulnerable older adults about the risks of heat waves and appropriate responses, providing recommendations for body cooling (e.g. applying ice towels, minimising clothing, using water-soaked cotton clothing, and employing fans with water applied to the body at air temperatures above 38°C, as older adults have lower sweating rates with advanced age)	Health	Professionals/policy makers	UN, 2022
532	Wearables and mobile health applications to data collection for self-monitoring of health status	Health	All population	WHO, 2015a
103	To develop the market for m-Health devices and services tailored for older adults (i.e, digital clinical records, automated reminders, prompts, and warnings integrated into clinical health-record systems to assist healthcare personnel in meeting quality standards, systematically documenting diagnostic test results, and recording the care provided)	Health	Old age people	Who, 2015a; EC, 2018; EC, 2021a; EC, 2022b
545	To provide clear and accessible information concerning health and social services available for older people	Health	Old age people	WHO, 2015a
110	Development of big data infrastructures	Technology	Professionals/policy makers	OECD, 2015
211	More general competencies in communication, teamwork, information technology and public health for health workers	Health	Professionals/policy makers	WHO, 2015a
197	To foster international cooperation on quality of care measurement and the exchange of best practices by implementing quality of care initiatives at various levels of governance. This includes sharing best practice guidelines, developing appropriate protocols and standards, standardising or aligning guidelines, and ensuring standardised packaging. Align different quality standards and interpretations across the EU while monitoring and exchanging information on national health initiatives	Health	Professionals/policy makers	WHO, 2012; WHO, 2017b; WHO, 2017c; UN, 2020; EC,2021b; EC, 2022a; EC, 2022b
235	To develop implementing bodies (e.g., labor inspection agencies and occupational health services) that provide employers with guidelines, run information campaigns, and carry out preventive actions on workplace safety	Labor	All population	OECD, 2017b
343	To improve health information and raise awareness of NCDs and their risk factors among professionals in various sectors (e.g. healthcare, education, workplace)	Labor	All population	EC, 2022a
469	To develop strong intergenerational communication, collaboration and solidarity, including older people and supporting them in engaging in climate actions, rather than pitting generations against each other	Environment	All population	WHO, 2021b; UN, 2022
120	To display distances in kilometres and travel times in minutes for car, public transportation, and walking	Transportation	All population	WHO, 2020
147	To ensure that information about transport options and timetables are available in accessible formats	Transportation	Old age people	WHO, 2015a

351	Policy and law and interventions for advocacy and communications could be guided by the reports of the Independent Expert on the enjoyment of all human rights by older persons and the evidence-based recommendations of the Global report on ageism (topic climate safety)	Environment	All population	WHO, 2021b; UN, 2022
64	To support communication activities about adaptable and smart home solutions towards users and suppliers	Housing	Old age people	EC, 2018
61	To combat ‘fake news’ and ‘infodemic’	Law and Crime	All population	EC, 2022a
342	To plan improvements in health information to increase awareness of NCDs and their risk factors among professionals in various sectors (e.g. health, education, etc.)	Health	Professionals/policy makers	EC, 2022a
344	To plan improvements in health information to increase awareness of NCDs and their risk factors among the general population and vulnerable groups	Health	All population	EC, 2022a
416	To raise awareness of the benefits of an active and healthy lifestyle and to incentivize prevention over treatment	Health	All population	EC, 2018
91	To develop and enforce compliance with accessibility standards in buildings, transportation, information and communication technologies, and other assistive technologies	Technology	Old age people	WHO, 2015b
56	To utilise communication policies, such as informative media campaigns, labelling requirements, and advertising restrictions, to shift consumer preferences away from harmful behaviours	Health	All population	OECD, 2019a
303	To implement a mass media campaign focused on prevention and health promotion	Health	All population	OECD, 2019a; EC, 2022b
404	Providing community education about disaster risk-management through brochures, posters, television and radio can be used to increase the visibility of older people and highlight both their needs and capacities and attention to the type of communication made to the seniors (consider sensory loss and low literacy)	Domestic commerce	All population	WHO, 2015a
372	To promote networks among stakeholders to foster ‘age-friendly’ cities and communities: Multi-interest participatory platforms (ensuring adequate representation will allow smaller settlements, such as towns and rural communities, to be included in national discussions, and provide a voice for underrepresented groups, including ethnic minorities, people with disabilities, indigenous populations, and those in low-income neighbourhoods)	Housing	All population	WHO, 2023
213	Help desks within local communities or online	Health	All population	EC, 2022a
396	To provide information in formats such as large print, “easy read” and pictures that meet the needs of older people, enabling them to make free and informed decisions	Health	Old age people	WHO, 2015b;WHO, 2017b
525	To use of geocoded maps to assess the accessibility of locations	Transportation	All population	WHO, 2020
517	To develop technologies for environmental information (for example, on air pollution)	Environment	All population	EC, 2018
336	To place health warnings on alcohol content	Health	All population	WHO, 2019b
106	To provide a bundle of scientifically reliable information, based on non-industry-funded research, for the general public	Health	All population	EC, 2022a
107	To develop a package of scientifically accurate information, based on research that is not industry-funded, for health professionals	Health	Professionals/policy makers	EC, 2022a
289	To implement legislation and regulations to restrict alcohol advertising, with a focus on more effectively framing such advertising, particularly in digital and social media	Agriculture	All population	OECD, 2019a; WHO, 2019b; WHO, 2019a; EC, 2022a

112	To provide nutritional guidance for the seniors to ensure that their meals are balanced. Dietary demands may fluctuate during the seniors' lifespan, necessitating recurring and adaptable counsel. Such advice should be designed in a way that allows the seniors to internalize the information	Health	Old age people	OECD, 2009
229	To implement a mass media campaign on healthy diets, including social marketing, to lower the intake of total fat, saturated fats, sugars, and salt, and encourage the intake of fruits and vegetables	Health	All population	EC, 2021b; EC, 2022a
290	To implement legislation and regulations to restrict the advertising of unhealthy foods	Agriculture	All population	OECD, 2019a
232	To implement nutrition labelling to reduce total energy intake (kcal), sugars, sodium and fats	Agriculture	All population	OECD, 2019a; WHO, 2019b; EC, 2022a
46	To ban the display of tobacco products at points of sale (EU tobacco control framework)	Domestic commerce	All population	EC, 2022a
462	Strengthen packaging rules by introducing mandatory plain standardised packaging with graphic health warnings covering 80% of the front and the back of all tobacco products and introducing pack inserts (EU tobacco control framework)	Domestic commerce	All population	WHO, 2019b; EC, 2022a
510	The advertising, promotion, and sponsorship (TAPS) ban should include tobacco brand names and corporate promotion (EU tobacco control framework)	Domestic commerce	All population	EC, 2022a; EC, 2021b
362	To promote programmes or exercise supported by the advice of a health professional accompanied by written material or advice from a primary-care professional and/or exercise specialist / provide advice about physical activity in all health and social care settings for older people, specifically targeting sedentary people, with a focus on promoting moderate intensity physical activity (particularly walking) and providing ongoing support	Health	Old age people	OECD, 2009; WHO, 2012
70	To promote continue to provide data on vaccine-preventable diseases and vaccination coverage among older people in order to obtain a better understanding of disease epidemiology	Health	All population	WHO, 2012
228	Implement evidence-based multicomponent falls prevention programs, including tailored exercise programs, physical therapy, and balance retraining. Increase access to preventive measures for high-risk groups of older adults, such as the use of assistive devices like hip protectors. Address environmental hazards through modifications and conduct medication reviews. Additionally, improve training and access to relevant information for informal caregivers in the community	Health	Old age people	WHO, 2012; WHO, 2016; WHO, 2017b; WHO, 2019b; OECD, 2019a
96	To develop coordinated approaches to more effectively frame advertising of food high in fat, sugar or salt, in particular on the digital and social media areas	Agriculture	All population	EC, 2022a
175	EU silver tourism roadmap	Culture	Old age people	EC, 2018
400	To provide social opportunities as well as accessible information on leisure and social activities	Culture	Old age people	WHO, 2015b; WHO, 2017b
364	To promote and support ‘age-friendly’ tourism destinations and tourist packages including accessibility and m-Health (e.g. age-friendly hotels)	Domestic commerce	All population	EC, 2018
160	To ensure access to essential medicines, vaccines, and innovations by promoting the use of generic medicines among both consumers and healthcare professionals	Health	Professionals/policy makers	WHO, 2019b
199	To foster self-management of service access by offering peer support, training, information, and advice to both older people and their caregivers	Health	All population	WHO, 2015a; WHO, 2015b; WHO, 2016
247	To increase the acceptability of greater taxes and transfers via openness of the process	Macroeconomics	All population	WHO, 2019a
53	To promote campaigns to raise awareness of ageism and knowledge on healthy ageing	Civil Rights	All population	WHO, 2015a; WHO, 2015b; WHO, 2017b; UN, 2020; EC, 2022a; UN 2022

154	To ensure that the media presents a balanced portrayal of aging	Civil Rights	All population	WHO, 2016; UN, 2020
273	Information about services should be available in different languages	Immigration	All population	EC, 2022a
339	Plan to address health literacy using a community-based approach: health education, teach people how to care for themselves and each other as they get older; educate and empower people on the services use; health information and media campaign (development of websites and brochures, and other publicity measures)	Education	All population	WHO, 2015b; WHO, 2017b; OECD, 2017a; OECD, 2019a; OECD, 2019b; UN 2020; EC, 2021b; EC, 2022a
509	Telehealth services for rural populations or remote monitoring of chronic patients (in rural areas) (cancer patients and survivors)	Health	Old age people	WHO, 2015a; OECD, 2017a; WHO, 2019c; OECD, 2019a; EC, 2021b
297	To connect health data across care settings	Health	All population	EC, 2018; OECD, 2019a
523	To use of call centres to communicate efficiently and cost-effectively with older people or their caregivers about the availability of services and to deliver standardised advices	Health	Old age people	WHO, 2017c
105	To develop digital patient records through the centralization of patient data, institutional platforms, data registries, and electronic health records, fostering an open data culture that ensures adequate, effective, and shared recording of medical information	Health	All population	OECD, 2015; EC, 2018; OECD, 2019a; EC, 2022b
524	To promote use of digital tools to support health promotion, prevention, and management	Health	All population	EC, 2022a
527	To use ICT for primary-care coordination in self-management of the therapy	Health	All population	OECD, 2009
214	To enhance understanding of data protection and privacy, address related legislative issues, and support 'open' data records for patients benefit	Health	Professionals/policy makers	EC, 2018; EC, 2022a; EC, 2022b; OECD, 2015
249	To improve the availability of non-communicable disease (NCD) data for decision-makers by creating mechanisms that enable effective communication between researchers and policymakers. To establish more effective methods to bridge the gap between knowledge generation and its application, considering the policy context. To facilitate the creation of relevant and timely evidence, conduct pertinent research on ageing and health, and identify cost-effective health system interventions suitable for local settings. To enhance communication by synthesising and packaging research findings in a way that policymakers can easily use. To empower decision-makers to utilise this information by fostering a culture that values evidence and its application.	Health	All population	WHO, 2015b; WHO, 2016; WHO, 2017b; EC, 2022a
488	To support the development of interoperable ICT solutions and their application across the EU	Technology	All population	EC, 2018
346	To plan initiatives that enhance support for patient empowerment and self-management of diabetes, chronic respiratory diseases, and cardiovascular disease (CVD), incorporating digital tools as integrated components of the care pathway used by both healthcare professionals and patients	Health	All population	OECD, 2019a; EC, 2022a
113	To enhance digital connectivity among workers for the purpose of knowledge exchange and collaboration	Labor	All population	EC, 2018; EC, 2021a; WHO, 2020; WHO, 2017c

90	To provide an interactive platform that connects people working on creating innovative solutions with older people who wish to assist and/or invest in company growth, exchange expertise with the younger generation, or participate in test-bed activities	Labor	All population	EC, 2018
118	Digital interventions include instruction in using the Internet and computers, as well as help for video communication	Social Welfare	Old age people	OECD, 2017b; WHO, 2021a
380	To promote the training of formal caregivers in the use of new digital technologies for older adults, including coaching and monitoring to support their development	Labor	Professionals/policy makers	EC, 2018; UN, 2020
265	To increase social dialogue	Labor	All population	EC, 2022b
516	To improve security perceptions, engage the media to reduce sensationalist reporting of crimes against older people and promote good pictures of older people engaged in their communities	Law and Crime	Old age people	WHO, 2015a
196	To foster intergenerational relations through positive media reporting and public image campaigns	Civil Rights	All population	WHO, 2012
474	To support and stimulate intergenerational dialogue and collaboration	Culture	All population	WHO, 2012; WHO, 2019c; UN, 2020
57	To encourage coalitions and initiatives to raise awareness about the concerns of social isolation in the seniors	Social Welfare	Old age people	WHO, 2021a
114	To implement digital interventions, including messaging services	Social Welfare	Old age people	WHO, 2021a
116	Digital interventions should include social networking sites	Social Welfare	Old age people	WHO, 2021a
117	To promote digital interventions include telephone befriending	Social Welfare	Old age people	WHO, 2021a
395	To provide information and opportunities for social activities to promote inclusion, participation, and reduce loneliness and social isolation, such as involvement in community organisations and charitable initiatives	Social Welfare	Old age people	WHO, 2015a; OECD, 2009; UN, 2020
354	Policymakers and all stakeholders, including the private sector, should ensure that information and communication technologies (ICTs) are available, affordable, and accessible to older adults	Technology	Old age people	WHO, 2021a; WHO, 2021b
460	Strategies and programs related to ICTs should incorporate accessibility requirements for digital information, products, and services designed to reduce social isolation and loneliness among older adults	Technology	Old age people	WHO, 2021a
115	Digital interventions should include online discussion groups and forums	Social Welfare	Old age people	WHO, 2021a
394	To provide information about leisure activities to promote inclusion, participation, and reduce loneliness and social isolation	Domestic commerce	Old age people	WHO, 2015a; UN, 2020
459	Strategies and programs related to ICTs should provide appropriate digital knowledge and training to help older adults adopt new technologies	Technology	Old age people	WHO, 2012: WHO, 2021a
355	Postal network development	Government Operations	All population	EC, 2021a
384	To safeguard the right to stay offline and offer alternatives for individuals who cannot or do not want to connect digitally.	Civil Rights	Old age people	WHO, 2021a
553	Advocacy/Media efforts to enhance knowledge of unpaid care work and gender stereotypes, employing local celebrities or artists that advocate for gender equality	Social Welfare	All population	UN Women, 2023
557	A more thorough approach places more emphasis on ability, affordability, and accessibility in order to assess and eliminate barriers to e-government. In addition to assessing the level of vulnerability and digital inclusion, these indicators can be used to direct proactive preventive actions and the development of targeted solutions. Developers have access to comprehensive data about digital access, affordability, and ability, which they may use to fill in some gaps in the design and implementation of e-government, increasing overall utilization rates and user satisfaction.	Governmental Operations	Professionals/policy makers	UN, 2022
558	The integrated e-government architecture focuses on improving data, design, and delivery (enablers) to eliminate barriers related to ability, affordability, and access in order to guarantee that no one is left	Governmental Operations	Professionals/policy makers	UN, 2022

	behind. This framework necessitates close coordination between departments, ministries, and affiliated organizations because digital projects typically have multiple facets			
567	To find the most effective ways to engage people, meet their needs, and assess the effects of digital services, governments should use data-driven, experimental, and AI-assisted data gathering techniques in addition to dynamic simulation models. New strategies that use systems thinking, foresight, pilot projects, and sandboxes to create and validate conceptual frameworks for innovative solutions should be created in order to take use of data-driven policy modelling.	Governmental Operations	Professionals/policy makers	UN, 2022

Strategy 11: Fostering better laws

ID	Actions	Policy sectors	Target	Report
86	To design win-win solutions that benefit both older and younger populations while rejecting zero-sum game narratives	Social Welfare	All population	WHO, 2022b
23	Advocacy for better services	Social Welfare	Old age people	WHO, 2017c
434	To develop a National Plan for Ageing and Health in collaboration with all relevant stakeholders, national and regional plans to foster Healthy Ageing. These plans should establish clear lines of responsibility, along with mechanisms for coordination, accountability, monitoring, and reporting across all relevant sectors. A national programme should be developed to support activities aligned with the WHO Global Network for Age-friendly Cities and Communities. National frameworks should be established for action on Healthy Ageing that engage all sectors, different levels of government, and civil society (including associations for older people, families, and carers), while ensuring an age-friendly primary care approach	Housing	Old age people	WHO, 2015a; WHO, 2015b; WHO, 2016; WHO, 2017b; OECD, 2017b; WHO, 2019c; WHO, 2020; OECD, 2021b; EC, 2022b
5	To abolish age-discriminatory laws	Civil Rights	Old age people	WHO, 2015b; WHO, 2017b; OECD, 2017a; WHO, 2019c; UN, 2020; WHO, 2020
82	To create formal structures and provide opportunities, capacity, and activities for translating research and evidence on ageing to inform policy-making	Health	Professionals/policy makers	WHO, 2015b; WHO, 2016; WHO, 2017b; WHO, 2021a
179	To regulate, select, and integrate evidence-based medical, health, and social services to adequately support older adults at home, in the community, or in institutions	Health	Old age people	WHO, 2016
197	To foster international cooperation on quality of care measurement and the exchange of best practices by implementing quality of care initiatives at various levels of governance. This includes sharing best practice guidelines, developing appropriate protocols and standards, standardising or aligning guidelines, and ensuring standardised packaging. Align different quality standards and interpretations across the EU while monitoring and exchanging information on national health initiatives	Health	Professionals/policy makers	WHO, 2012; WHO, 2017b; WHO, 2017c; UN, 2020; EC,2021b; EC, 2022a; EC, 2022b
260	To increase levels of public spending and investments in care provision, ensuring a balanced approach between private and public provision while reinforcing solid universal public services	Health	All population	WHO, 2016; WHO, 2017b; WHO, 2017c; OECD,

				2019a; OECD, 2019b; WHO, 2021b; EC, 2021a; EC, 2022b
347	To plan an increase in the capacity of healthcare systems to identify and treat individuals with unhealthy diets and to enhance brief interventions	Health	All population	EC, 2022a
425	To integrate global evidence on effective practices in diverse contexts and establish basic standards to encourage the testing of approaches that further develop systems of long-term care, whether home-based, community-based, or institutional	Health	All population	WHO, 2015b; WHO, 2017b
365	To promote and support multi-sectoral and intersectoral collaboration with diverse stakeholders (partnership) to design and evaluate actions to foster functional ability in ageing	Health	Old age people	WHO, 2015b; WHO, 2016; WHO, 2017b; WHO, 2020
95	To develop contingency plans for humanitarian emergencies that ensure an inclusive response for all ages. Emergency supplies, such as medical equipment, backup generators, food, and water, should be prepared and stockpiled for long-term care facilities, including healthcare centres. Older adults should be actively involved in developing policies, legislation, and programs, as well as in monitoring their implementation	Domestic commerce	Old age people	WHO, 2015a; UN, 2020; UN, 2022
348	To plan an increase in the capacity of health systems to identify and treat individuals with issues related to physical activity or inactivity, and to enhance brief interventions and physical activity prescriptions	Health	All population	WHO, 2019b; EC, 2022a
235	To develop implementing bodies (e.g., labor inspection agencies and occupational health services) that provide employers with guidelines, run information campaigns, and carry out preventive actions on workplace safety	Labor	All population	OECD, 2017b
150	To ensure that older people are provided with priority seating	Transportation	Old age people	WHO, 2015a
351	Policy and law and interventions for advocacy and communications could be guided by the reports of the Independent Expert on the enjoyment of all human rights by older persons and the evidence-based recommendations of the Global report on ageism (topic climate safety)	Environment	All population	WHO, 2021b; UN, 2022
468	Strong community engagement to ensure linkage and integration between climate issue and primary health care	Health	Old age people	UN, 2022
166	To establish a focal point on ageing and health within the Ministry of Health	Health	All population	WHO, 2017b; WHO, 2020
372	To promote networks among stakeholders to foster ‘age-friendly’ cities and communities: Multi-interest participatory platforms (ensuring adequate representation will allow smaller settlements, such as towns and rural communities, to be included in national discussions, and provide a voice for underrepresented groups, including ethnic minorities, people with disabilities, indigenous populations, and those in low-income neighbourhoods)	Housing	All population	WHO, 2023
125	To encourage data-sharing and linkages across sectors (such as health, social welfare, labour, education, environment, transportation)	Government Operations	Professionals/policy makers	WHO, 2015b;WHO, 2017b
296	To link the monitoring of Healthy Ageing metrics to the evaluation of national, intersectoral, and multisectoral policies and programs, ensuring alignment with other international efforts, such as the Sustainable Development Goals	Government Operations	Old age people	WHO, 2015b;WHO, 2017b
285	Learning and Collaboration Between Countries	Government Operations	All population	WHO, 2017c
60	To promote collaboration beyond the environment and health sectors to encompass infrastructure, employment, education, social protection, housing, energy, and transport, particularly in cities with high concentrations of older people and in small island developing states	Government Operations	All population	WHO, 2017b; UN, 2022

289	To implement legislation and regulations to restrict alcohol advertising, with a focus on more effectively framing such advertising, particularly in digital and social media	Agriculture	All population	OECD, 2019a; WHO, 2019b; WHO, 2019a; EC, 2022a
290	To implement legislation and regulations to restrict the advertising of unhealthy foods	Agriculture	All population	OECD, 2019a
326	To create partnerships to promote health across sectors, professionals, between public, private, and non-government agencies	Health	All population	OECD, 2009
102	To develop tax benefits to promote physical activity	Macroeconomics	All population	EC, 2022a
35	To allocate adequate resources to implement action plans while ensuring that public resources are effectively managed to facilitate Healthy Ageing	Health	Professionals/policy makers	WHO, 2015b; WHO, 2017b
313	National and subnational health plans should be reviewed and revised considering a variety of climate-related situations, such as prolonged, extreme heat waves, sudden floods or spikes in air pollution, opportunities to promote and support older people's resilience to climate change	Health	Old age people	UN, 2022
170	To establish programs to improve diet by implementing national regulations on prepared or processed foods	Agriculture	All population	OECD, 2009
341	To plan to effectively promote strategies such as shelf placement, promotional policies, packaging of private brands, and nudging in major distribution channels (supermarkets) to encourage consumers to choose healthier options	Domestic commerce	All population	EC, 2022a
261	To increase national commitment to healthy ageing strategies by establishing a broad political and operational platform that enables and legitimises effective multi-sectoral actions	Government Operations	Old age people	WHO, 2017b; WHO, 2020
322	To orient health systems around the intrinsic capacity and functional ability of older adults. Sustainably finance the programs, services, and system realignments needed to promote Healthy Ageing	Health	Old age people	WHO, 2015b; WHO, 2017b
450	To set national targets for providing one balanced meal a day	Health	Old age people	OECD, 2009
68	Connection with World report on ageing and health	Health	Professionals/policy makers	WHO, 2016
34	To align strategies with the "green transition" by encouraging urban and city planners, architects, and engineers to identify climate-adaptive, nature-based solutions for green (vegetated) and blue (water) spaces. These solutions provide significant benefits for both people and biodiversity in cities and include increasing the number of parks, tree-lined streets, paths, urban gardens, green roofs, and building facades	Environment	All population	EC, 2022a; UN 2022
32	To align air quality standards with EU and WHO recommendations	Environment	All population	EC, 2021b
33	To align mobility policies with the European Sustainable and Smart Mobility Strategy	Transportation	All population	EC, 2021b
133	To enforce existing regulations on tobacco availability to limit access	Macroeconomics	All population	WHO, 2019a; EC, 2022a; OECD, 2019a
78	To coordinate and support the enforcement of existing regulations	Law and Crime	Professionals/policy makers	EC, 2022a
164	To ensure the implementation of legislation related to novel tobacco products and avoid legislative gaps in response to new forms of use	Domestic commerce	All population	EC, 2022a
159	To ensure access to essential medicines, vaccines, and innovations by facilitating the early market entry of generic essential medicines	Health	All population	WHO, 2019b
188	Financing for long-term care will be crucial and may involve incorporating long-term care into social insurance. This insurance scheme should include individual needs assessments and offer a range of	Health	All population	WHO, 2015a; WHO, 2021b

	services, such as home care, domestic assistance, public health nurses, day care, and short-term stay centres			
277	To introduce or strengthen legislation that promotes non-discrimination, on the basis of aspects such as gender, in the provision of health services	Civil Rights	Old age people	WHO, 2019c
28	To promote age-discrimination legislation in life-long learning	Civil Rights	Old age people	WHO, 2015b; WHO, 2017b; UN, 2020; WHO, 2020
27	To implement age-discrimination legislation in employment	Labor	Old age people	WHO, 2015b; WHO, 2017b; OECD, 2017a; WHO, 2019c; UN, 2020; WHO, 2020
29	To implement age-discrimination legislation to ensure participation and access to labour benefits	Labor	Old age people	UN, 2020; WHO, 2017b; WHO, 2019c
121	To diversify the tax mix for health and long-term care financing to optimise a combination that generates stable and sufficient revenue in an equitable manner. Identify and implement sustainable mechanisms for resourcing long-term care	Macroeconomics	All population	WHO, 2015b; WHO, 2017b; WHO, 2019a
268	To increase the visibility of older persons within the Sustainable Development Goals	Civil Rights	Old age people	WHO, 2020
74	To contribute to the Global Campaign to Combat Ageism by building a global coalition to improve data collection, share knowledge, and coordinate efforts to prevent and respond to ageism	Civil Rights	Professionals/policy makers	WHO, 2021b; WHO, 2022a
349	To plan to reduce health inequalities, specifically by addressing social determinants (i.e. education level)	Education	All population	EC, 2022a
139	To ensure mechanisms for the enforcement of age-discrimination legislation	Civil Rights	Old age people	WHO, 2015b; WHO, 2017b; UN, 2020; WHO, 2020; WHO, 2021a
299	To cover long-term care costs through a comprehensive social protection system, ensuring that out-of-pocket expenses for recipients of home care remain low enough for them to afford other essential living expenses, such as food, housing, and heating	Health	All population	WHO, 2017b; OECD, 2017a; UN, 2020; WHO, 2020; EC, 2021a; EC, 2022b
156	To ensure the development and implementation of national care standards, guidelines, protocols for person centred integrated long-term care provision	Health	Professionals/policy makers	WHO, 2015b
249	To improve the availability of non-communicable disease (NCD) data for decision-makers by creating mechanisms that enable effective communication between researchers and policymakers. To establish more effective methods to bridge the gap between knowledge generation and its application, considering the policy context. To facilitate the creation of relevant and timely evidence, conduct pertinent research on ageing and health, and identify cost-effective health system interventions suitable for local settings. To enhance communication by synthesising and packaging research findings in a way that policymakers can easily use. To empower decision-makers to utilise this information by fostering a culture that values evidence and its application.	Health	All population	WHO, 2015b; WHO, 2016; WHO, 2017b; EC, 2022a
104	To develop coordination mechanisms between healthcare and long-term care, as nursing homes can serve as useful sites for sheltering community-dwelling individuals who require care during and immediately after a disaster	Health	Old age people	WHO, 2015a

4	To establish a national policy on long-term care that encompasses homes, communities, and institutions, while encouraging the testing of approaches to further develop home-, community-, and institution-based care systems	Health	All population	WHO, 2012; WHO, 2015a; WHO, 2017a; OECD, 2019a; WHO, 2020; EC, 2022b; WHO, 2022b
171	To establish clearer protocols for drug use and implement methods to ensure compliance with these protocols. In this context, disease or case-management programs can serve as effective vehicles for implementing such arrangements	Health	All population	OECD, 2009
316	To nurture leadership and build capacity at all levels for integrated action across sectors, engaging all sectors and administrative levels. To establish collaborative leadership through a new governance model based on cooperation within government, across sectors and stakeholders, and among different levels of government to achieve the goals of the UN Decade of Healthy Ageing	Government Operations	All population	UN, 2020; WHO, 2023
278	To introduce new technologies in a systematic way	Technology	Professionals/policy makers	WHO, 2019b
283	To involve older people themselves in service planning	Health	Old age people	WHO, 2016
353	To guide policy, law, and human resources interventions by the reports of the Independent Expert on the enjoyment of all human rights by older persons, along with the evidence-based recommendations from the Global Report on Ageism	Labor	All population	WHO, 2021b; UN, 2022
491	To support the labour unions	Labor	All population	EC, 2022b
536	To enhance knowledge of worker rights	Labor	All population	EC, 2022b
539	To improve working conditions for those providing services to older people by empowering care workers with meaningful decision-making authority	Labor	All population	WHO, 2015a; WHO, 2015b
437	To respond to older people abuse in the community by encouraging the prosecution of offenders	Law and Crime	Old age people	UN, 2020
282	Involve older people in political processes that impact their rights by engaging them in the planning, implementation, and evaluation of local health, social services, and recreation programs, as well as in the development of research agendas on active ageing (both as advisors and investigators). This includes fostering broader coalitions and ensuring the political incorporation of minority groups, as well as actively involving older adults in policies and laws that concern them	Civil Rights	Old age people	WHO, 2015a; WHO, 2016; WHO, 2017b; WHO, 2019c; WHO, 2021b; WHO, 2021b
352	To guide policy, law, and interventions in education by using the reports of the Independent Expert on the enjoyment of all human rights by older persons, along with the evidence-based recommendations from the Global Report on Ageism	Civil Rights	All population	WHO, 2021b; UN, 2022
556	To make plans to reduce digital inequality through e-governance, assisting underprivileged groups left out of traditional social protection programs and enabling seniors and people with disabilities to access digital services	Technology	Professionals/policy makers	UN, 2022
558	The integrated e-government architecture focuses on improving data, design, and delivery (enablers) to eliminate barriers related to ability, affordability, and access in order to guarantee that no one is left behind. This framework necessitates close coordination between departments, ministries, and affiliated organizations because digital projects typically have multiple facets	Governmental Operations	Professionals/policy makers	UN, 2022
559	A dynamic data-design-delivery framework that integrates the quickly evolving digital age tools and technology and promotes the development of well-thought-out and grounded e-government	Governmental Operations	Professionals/policy makers	UN, 2022

568	Enacting comprehensive national legislation to combat the problem of elder abuse includes establishing standards for what behaviours are acceptable and unacceptable, protecting survivors legally, coordinating the response of all pertinent parties, and punishing those who engage in such abuse. In addition to taking into consideration the unique needs of older people as a non-homogeneous group—particularly the intersectional and cumulative factors that increase inequality and raise risks in older age—laws and policies pertaining to violence, abuse, and neglect must be in line with human rights, including the principles of equality and non-discrimination	Civil Rights	Professionals/policy makers	UNECE, 2022; UN, 2023
569	Adopting and implementing national action plans that enable states to consider the underlying causes of abuse and its effects on their elderly populations is one way to prevent elder abuse. These plans should include and define goals, priorities, assigned responsibilities, a schedule and evaluation system, and sufficient funding for implementation	Civil Rights	Professionals/policy makers	UN, 2023
572	Cooperation between social services, the criminal justice system, the health sector, and civil society to stop elder abuse: When older people ask for assistance leaving violent environments, develop a top-notch multi-sectoral response	Civil Rights	Professionals/policy makers	UN, 2023
575	To make ageism a top political priority	Civil Rights	Professionals/policy makers	UN, 2023
576	To include senior citizens in the design and development of more age-friendly goods and services	Macroeconomics	Professionals/policy makers	UNECE, 2022

Strategy 12: Monitoring and evaluation for better policy making

ID	Actions	Policy sectors	Target	Report
304	To assess age-friendliness of communities and apply an equity lens	Health	Old age people	WHO, 2019c
89	Develop accountability systems, such as the use of equality impact evaluation and indicators, to strengthen personal and community skills.	Health	All population	WHO, 2019c
302	To create a case for investment by examining and presenting the costs and advantages of pursuing, preventing and reducing the effects of older people abuse	Civil Rights	Old age people	WHO, 2022a
434	To develop a National Plan for Ageing and Health in collaboration with all relevant stakeholders, national and regional plans to foster Healthy Ageing. These plans should establish clear lines of responsibility, along with mechanisms for coordination, accountability, monitoring, and reporting across all relevant sectors. A national programme should be developed to support activities aligned with the WHO Global Network for Age-friendly Cities and Communities. National frameworks should be established for action on Healthy Ageing that engage all sectors, different levels of government, and civil society (including associations for older people, families, and carers), while ensuring an age-friendly primary care approach	Housing	Old age people	WHO, 2015a; WHO, 2015b; WHO, 2016; WHO, 2017b; OECD, 2017b; WHO, 2019c; WHO, 2020; OECD, 2021b; EC, 2022b
253	To develop incentives to boost education and training of data analysts and bioinformatics professionals in using big data	Health	Professionals/policy makers	OECD, 2015
532	Wearables and mobile health applications to data collection for self-monitoring of health status	Health	All population	WHO, 2015a
142	To guarantee that older individuals are meaningfully and statistically represented in population-based research with adequate power to analyse data, as well as included in therapeutic trials and other studies (e.g., climate change effects). Older persons are included in clinical trials because they are most likely to	Health	Old age people	OECD, 2009; WHO, 2015b; WHO, 2017b; UN, 2022

	utilize the medications. This will allow greater knowledge on dose, effectiveness, and long-term impacts of an unwanted kind			
82	To create formal structures and provide opportunities, capacity, and activities for translating research and evidence on ageing to inform policy-making	Health	Professionals/policy makers	WHO, 2015b; WHO, 2016; WHO, 2017b; WHO, 2021a
197	To foster international cooperation on quality of care measurement and the exchange of best practices by implementing quality of care initiatives at various levels of governance. This includes sharing best practice guidelines, developing appropriate protocols and standards, standardising or aligning guidelines, and ensuring standardised packaging. Align different quality standards and interpretations across the EU while monitoring and exchanging information on national health initiatives	Health	Professionals/policy makers	WHO, 2012; WHO, 2017b; WHO, 2017c; UN, 2020; EC,2021b; EC, 2022a; EC, 2022b
270	Infectious disease control programmes in institutions, extending beyond hospitals to take in other facilities, including those for older people	Health	Old age people	WHO, 2012
80	To establish a national multi-stakeholder forum or committee on ageing and health to share experiences, best practices, and lessons learned: Encourage collaboration among important stakeholders, such as care-dependent individuals and their caregivers, non-profit groups, and the public and commercial sectors, to offer long-term care and health promotion	Government Operations	Old age people	WHO, 2015b; WHO, 2016; WHO, 2017b; WHO, 2020; UN 2020
198	To foster international exchanges of information on best practices in the evaluation and promotion of continuous training in competencies for the health and social care of older people	Health	Professionals/policy makers	WHO, 2012
95	To develop contingency plans for humanitarian emergencies that ensure an inclusive response for all ages. Emergency supplies, such as medical equipment, backup generators, food, and water, should be prepared and stockpiled for long-term care facilities, including healthcare centres. Older adults should be actively involved in developing policies, legislation, and programs, as well as in monitoring their implementation	Domestic commerce	Old age people	WHO, 2015a; UN, 2020; UN, 2022
254	To include an assessment of eating habits during geriatric evaluations	Health	Old age people	OECD, 2009
67	To perform periodic, population-based monitoring of older people, including those in long-term care facilities; increasing the ability of surveys and reporting systems to monitor health and social care services, particularly preventative programs, and their usage and access by older people	Health	Old age people	WHO, 2012; WHO, 2015b; WHO, 2017b
126	To encourage monitoring, surveillance, and reporting in accordance with accepted worldwide metrics	Health	Professionals/policy makers	WHO, 2015b;WHO, 2017b; EC, 2022b
140	To ensure the monitoring of long-term care quality, its impact on functional ability and well-being, and the continuous improvement of care based on outcome	Health	All population	WHO, 2015b; WHO, 2016; WHO, 2017b; UN, 2020; EC, 2022a; EC, 2022b
51	Better clinical research is urgently needed on the aetiology and treatments of key health conditions affecting older adults, including musculoskeletal and sensory impairments, cardiovascular diseases and risk factors such as hypertension and diabetes, mental disorders, dementia and cognitive decline, cancer, and geriatric syndromes such as frailty	Health	Old age people	WHO, 2016
436	Research on the Causes and Interventions for Frailty	Health	Old age people	WHO, 2017c

37	To evaluate national health system responses to ageing populations and propose recommendations for realignment	Health	Old age people	WHO, 2015b; WHO, 2016; WHO, 2017b
176	To assess the economic and quality-of-life impact on the home and caregivers of older adults	Health	All population	WHO, 2017c
320	To orient health systems around the intrinsic capacity and functional ability of older people: To adapt information systems for collecting, analysing, and reporting data on intrinsic capacity and trends in capacity. Data on intrinsic capacity and functional ability should be collected, analysed, and reported, disaggregated by age, sex, and other intersectional variables	Health	Old age people	WHO, 2015b; WHO, 2017b; WHO, 2021b
31	To agree on methodologies to measure, analyse, characterize, and monitor Healthy Ageing, which addresses critical policy concerns and older people's expectations	Health	All population	WHO, 2016; WHO, 2017b
466	Improving data, research, and innovation to speed the implementation of healthy aging policy	Health	All population	WHO, 2015b; WHO, 2020
141	To guarantee that national vital registration and data are disaggregated by age and gender throughout the life course, as well as essential social and economic aspects	Government Operations	Old age people	WHO, 2017b
296	To link the monitoring of Healthy Ageing metrics to the evaluation of national, intersectoral, and multi-sectoral policies and programs, ensuring alignment with other international efforts, such as the Sustainable Development Goals	Government Operations	Old age people	WHO, 2015b;WHO, 2017b
70	To promote continue to provide data on vaccine-preventable diseases and vaccination coverage among older people in order to obtain a better understanding of disease epidemiology	Health	All population	WHO, 2012
72	Continuous monitoring of tobacco use and of the impact of the tobacco control policy	Health	All population	EC, 2022a
77	To create cooperation and sharing of experience and good practice on effective measures to increase physical activity levels among older persons, in order to support their implementation and evaluation	Health	Old age people	WHO, 2012
138	To ensure effective coordination across sectors of implementation and monitoring of interventions and policies, for example through task forces	Government Operations	Old age people	WHO, 2015b; WHO, 2017b
246	Improved monitoring, modelling and air quality plans will help local authorities to achieve cleaner air	Environment	All population	EC, 2021b
85	To redact design/review and pilot-test evidence-based guidelines for population and/or opportunistic screening on diabetes, CVD and clinical risk factors, including, for example, the joint definition of ethical guidance, minimum standards, and packages for the training of healthcare professionals	Health	Professionals/policy makers	EC, 2022a
177	To evaluate cost of care and barriers to access	Health	Old age people	WHO, 2017c
74	To contribute to the Global Campaign to Combat Ageism by building a global coalition to improve data collection, share knowledge, and coordinate efforts to prevent and respond to ageism	Civil Rights	Professionals/policy makers	WHO, 2021b; WHO, 2022a
455	Social stratification in health records to assist the assessment of health inequalities / evidence-based and age-and sex-disaggregated information regarding aging and health and the contribution of older persons / evaluating health policies and programs in connection to inequities	Health	All population	OECD, 2015; WHO, 2015a; WHO, 2015b; WHO, 2017b; WHO, 2021b; EC, 2022a
242	To improve the evidence base for older people maltreatment and strengthen capacity for research on effective interventions	Civil Rights	Old age people	WHO, 2012
279	To invest in data collecting on the costs of older people abuse and the cost-effectiveness of treatments. Create a database with extensive information on the treatments, the rigor with which they were evaluated, and the type of abuse targeted. The database should also contain a summary of the most promising policies, legislation, and human rights instruments	Civil Rights	Professionals/policy makers	WHO, 2016; WHO, 2022a
41	To automate the storing of this information on trajectories of functioning of the aged, such that patterns in functioning over time may be routinely established	Health	Old age people	WHO, 2016

65	Comprehensive assessments of older people's health and social care needs: Using cross-sectional and longitudinal nationally representative data on older people's health status and needs; data collection on premature mortality, lifestyle behaviours, other risk factors (e.g., obesity, blood pressure, blood sugar level), and NCD severity; collection and dissemination of disaggregated information on age and healthy aging; to implement longitudinal community-level data collection; collection of age and socioeconomic disaggregated information; collection of health information on ability and capacity evaluations, intrinsic capacity, and functional ability; focus on risk and protective factors	Health	All population	WHO, 2012; Who, 2015b; WHO, 2017b; WHO, 2017c; OECD, 2019b; WHO, 2020; UN, 2020; EC, 2022a; WHO, 2022a
248	To strengthen surveys and reporting systems for aging populations, and to stimulate the interchange of novel forms of providing care that are sensitive to the requirements of older persons	Health	All population	WHO, 2012; WHO, 2017b; WHO, 2017c
214	To enhance understanding of data protection and privacy, address related legislative issues, and support 'open' data records for patients benefit	Health	Professionals/policy makers	EC, 2018; EC, 2022a; EC, 2022b; OECD, 2015
249	To improve the availability of non-communicable disease (NCD) data for decision-makers by creating mechanisms that enable effective communication between researchers and policymakers. To establish more effective methods to bridge the gap between knowledge generation and its application, considering the policy context. To facilitate the creation of relevant and timely evidence, conduct pertinent research on ageing and health, and identify cost-effective health system interventions suitable for local settings. To enhance communication by synthesising and packaging research findings in a way that policymakers can easily use. To empower decision-makers to utilise this information by fostering a culture that values evidence and its application.	Health	All population	WHO, 2015b; WHO, 2016; WHO, 2017b; EC, 2022a
470	To map national employment and social protection programs by researching cancer survivors' return to work	Labor	All population	EC, 2021b
88	Develop a standardised international tools to measure social isolation and loneliness	Health	Professionals/policy makers	WHO, 2021a
173	To estimate effectiveness, costs, and cost-effectiveness of interventions to reduce social isolation and loneliness	Health	Old age people	WHO, 2021a
551	Long-term monitoring and the application of time-use models within household consumption surveys, as well as the integration of time-use surveys into national data systems to capture the multitasking aspect of unpaid care work	Social Welfare	Professionals/policy makers	UN Women, 2023
557	A more thorough approach places more emphasis on ability, affordability, and accessibility in order to assess and eliminate barriers to e-government. In addition to assessing the level of vulnerability and digital inclusion, these indicators can be used to direct proactive preventive actions and the development of targeted solutions. Developers have access to comprehensive data about digital access, affordability, and ability, which they may use to fill in some gaps in the design and implementation of e-government, increasing overall utilization rates and user satisfaction	Governmental Operations	Professionals/policy makers	UN, 2022
558	The integrated e-government architecture focuses on improving data, design, and delivery (enablers) to eliminate barriers related to ability, affordability, and access in order to guarantee that no one is left behind. This framework necessitates close coordination between departments, ministries, and affiliated organizations because digital projects typically have multiple facets	Governmental Operations	Professionals/policy makers	UN, 2022

564	The integrated e-government architecture focuses on improving data, design, and delivery (enablers) to eliminate barriers related to ability, affordability, and access: monitoring, evaluation, and learning framework. This ensures that no one is left behind	Governmental Operations	Professionals/policy makers	UN, 2022
565	Ensuring that adequate financial, political and human resources to the e-government goals	Governmental Operations	Professionals/policy makers	UN, 2022
566	Governments can streamline and optimize the use of IT resources by utilizing cloud technology (public, private, hybrid, and multi-vendor), which encourages the adoption of new digital technologies: Cloud technology offers	Governmental Operations	Professionals/policy makers	UN, 2022
567	To find the most effective ways to engage people, meet their needs, and assess the effects of digital services, governments should use data-driven, experimental, and AI-assisted data gathering techniques in addition to dynamic simulation models. New strategies that use systems thinking, foresight, pilot projects, and sandboxes to create and validate conceptual frameworks for innovative solutions should be created in order to take use of data-driven policy modelling	Governmental Operations	Professionals/policy makers	UN, 2022
570	Monitoring procedures at health care and long-term care facilities to shield senior citizens from deprivations of freedom that could lead to abuse, neglect, and violent crimes	Civil Rights	Professionals/policy makers	UN, 2023

Strategy 13: Research and Development

ID	Actions	Policy sectors	Target	Report
280	To promote investment on technologies and ICT (i.e. e-health and telemedicine)	Health	All population	WHO, 2021b
225	Identify social-psychological determinants of unhealthy ageing	Health	Professionals/policy makers	EC, 2022b
82	To create formal structures and provide opportunities, capacity, and activities for translating research and evidence on ageing to inform policy-making	Health	Professionals/policy makers	WHO, 2015b; WHO, 2016a; WHO, 2017b; WHO, 2021a
179	To regulate, select, and integrate evidence-based medical, health, and social services to adequately support older adults at home, in the community, or in institutions	Health	Old age people	WHO, 2016a
206	To guide research and innovation to ensure that public and private sector developers and providers (including health and care services, medical devices, and pharmaceuticals) meet the needs of older adults, including those with limited resources	Health	Professionals/policy makers	WHO, 2015b; WHO, 2017b
314	To promote the new assistive technologies that may extend the autonomy, independence and participation of older people with disabilities	Social Welfare	All population	WHO, 2015b; WHO, 2016a; EC, 2021b
224	Identify research gaps and encourage research in these areas	Health	All population	WHO, 2017bf
531	To promote Wearables and mobile health applications to data collection for research	Health	Old age people	EC, 2021b
285	Learning and Collaboration Between Countries	Government Operations	All population	WHO, 2017c
379	To promote the development of globally competitive products, including wearable technologies, functional foods, personalized nutrition, and preventive medicine	Foreign Trade	All population	EC, 2018
178	To provide evidence for identify critical periods for action to prevent unhealthy ageing	Health	All population	WHO, 2016a

435	To promote the research on research on the connections between ageism, other forms of prejudice and discrimination, and older people abuse	Civil Rights	Old age people	WHO, 2022a
268	To increase the visibility of older persons within the Sustainable Development Goals	Civil Rights	Old age people	WHO, 2020bf
242	To improve the evidence base for older people maltreatment and strengthen capacity for research on effective interventions	Civil Rights	Old age people	WHO, 2012
488	To support the development of interoperable ICT solutions and their application across the EU	Technology	All population	EC, 2018
278	To introduce new technologies in a systematic way	Technology	Professionals/policy makers	WHO, 2019b
184	To exploit innovative technology to improve efficiency in care	Health	All population	EC, 2021b
66	To support comprehensive Geriatric Assessment & Research	Health	Old age people	WHO, 2017c
90	To provide an interactive platform that connects people working on creating innovative solutions with older people who wish to assist and/or invest in company growth, exchange expertise with the younger generation, or participate in test-bed activities.	Labor	All population	EC, 2018
445	To encourage the robotics and gaming industries to prioritise the needs and interests of older adults, collaborating to develop enjoyable products that reduce loneliness, promote mental health	Technology	Old age people	EC, 2018
486	To support research around driverless cars and public transport for the older customer	Transportation	Old age people	EC, 2018
282	Involve older people in political processes that impact their rights by engaging them in the planning, implementation, and evaluation of local health, social services, and recreation programs, as well as in the development of research agendas on active ageing (both as advisors and investigators). This includes fostering broader coalitions and ensuring the political incorporation of minority groups, as well as actively involving older adults in policies and laws that concern them	Civil Rights	Old age people	WHO, 2015a; WHO, 2016a; WHO, 2017b; WHO, 2019c; Greer, 2021; WHO, 2021b
463	To strengthen research capacities and incentives for innovation by promoting multidisciplinary research that incorporates older adults in all stages, addressing their needs and preferences, including in quality of care research	Health	Old age people	WHO, 2015b; WHO, 2016a; WHO, 2017b; WHO, 2017c; UN, 2020; EC, 2022a

Strategy 14: Miscellanea

ID	Actions	Policy sectors	Target	Report
215	Helplines to provide information to anonymous callers and referrals for actual/potential victims of age discrimination	Civil Rights	Old age people	WHO, 2015a
49	To promote bank use safety – this can be monitored to detect suspicious patterns and may help to identify older people at risk of financial abuse	Law and Crime	Old age people	WHO, 2015a
311	Multidisciplinary teams of professionals from various disciplines who cooperate to address and resolve cases of older people abuse	Civil Rights	Old age people	WHO, 2015a
95	Develop contingency plans for humanitarian emergencies to ensure an age inclusive response: Emergency supplies such as medical supplies, back-up generators and food and water should be	Domestic commerce	Old age people	WHO, 2015a; UN, 2020; UN, 2022

	prepared and stocked for long-term care facilities, as in health facilities / Older people should be involved in the development of policies, legislation and programmes, and in monitoring implementation			
404	Providing community education about disaster risk-management through brochures, posters, television and radio can be used to increase the visibility of older people and highlight both their needs and capacities and attention to the type of communication made to the seniors (consider sensory loss and low literacy)	Domestic commerce	All population	WHO, 2015a
202	Promote genetic counselling programmes	Health	All population	EC, 2022a
288	Legislating the use of international non-proprietary names	Health	All population	WHO, 2019b
522	To promote the use of guidance and tools to prevent ageism and older people abuse in care provision	Civil Rights	Old age people	WHO, 2015a; WHO, 2017b; UN, 2020; WHO, 2022a
123	Emergency shelter provided for victims of older people abuse	Civil Rights	Old age people	WHO, 2015af
162	Ensuring that persons with a minority racial or ethnic origin may fully realize their potential would result in improved social and economic consequences for everybody.	Civil Rights	All population	EC, 2021a
137	Ensure balanced distribution of workforce within countries and development of workforce to match demand for services	International Affairs	All population	WHO, 2015b; WHO, 2017bf
479	To support immigration of highly educated people	Immigration	All population	EC, 2021a
437	To respond to older people abuse in the community by encouraging the prosecution of offenders	Law and Crime	Old age people	UN, 2020

3 List of reports included

1. **European Commission 2018** Technopolis Group United Kingdom. The Silver Economy: Final Report. A study prepared for the European Commission, DG Communications Networks, Content & Technology. European Union. 2018. DOI 10.2759/685036
2. **European Commission 2021a** EU Green Paper on Ageing: Fostering solidarity and responsibility between generations. 2021
3. **European Commission 2021b.** 2021 Long-Term Care Report: Trends, challenges and opportunities in an ageing society. Volume I. Joint Report prepared by the Social Protection Committee (SPC) and the European Commission (DG EMPL). European Union. 2021
4. **European Commission 2021c.** Europe's Beating Cancer Plan Communication from the commission to the European Parliament and the Council. 2021
5. **European Commission 2022a.** Healthier Together EU Non-Communicable Diseases Initiative. European Union. 2022
6. **European Commission 2022b.** Commission Staff Working Document. Summary of consultation activities. Accompanying the document Commission Communication on the European care strategy. 2022
7. **OECD 2009** Oxley H. Policies for Healthy Ageing: An Overview. OECD Health Working Papers No. 42. 2009 DOI 10.1787/226757488706
8. **OECD 2015** OECD. Ageing: Debate the Issues. OECD Insights, OECD Publishing, Paris. 2015. DOI 10.1787/9789264242654-en
9. **OECD 2017a** OECD. Preventing Ageing Unequally. OECD Publishing, Paris. 2017. DOI 10.1787/9789264279087-en
10. **OECD 2017b** OECD. Meeting of the OECD Council at Ministerial Level. Preventing Ageing Unequally - Action Plan. Paris. 2017
11. **OECD 2019a** OECD. Promoting Healthy Ageing Background report for the 2019 Japanese G20 Presidency. 2019
12. **OECD 2019b** OECD. Promoting Healthy Ageing. 2019
13. **United Nation 2020** UN Decade of Healthy Ageing: Plan of Action 2021–2030. United Nation. 2020
14. **United Nations 2022** UN Decade of Healthy Ageing 2021-2030 in a Climate-changing World. 2022
15. **United Nations 2023** United Nation. General Assembly. Violence against and abuse and neglect of older persons Report of the Independent Expert on the enjoyment of all human rights by older persons, Claudia Mahler. Human Rights Council. Fifty-fourth session. 11 September–6 October 2023

16. **United Nations Economic Commission for Europe 2022** A sustainable world for all ages. Joining Forces for Solidarity and Equal Opportunities Throughout Life. Proceedings of the 2022 UNECE Ministerial Conference on Ageing 16-17 June 2022 Rome, Italy

17. **United Nation Women 2023** Baseline study on care economy in Bosnia and Herzegovina. Overview of the Key Denominators, Policy and Programming Options. Sarajevo, Bosnia and Herzegovina. UN Women. 2023

18. **WHO 2012** World Health Organization. Regional Office for Europe. Strategy and action plan for healthy ageing in Europe, 2012–2020. Regional Committee for Europe. Sixty-second session. Malta, 10–13 September 2012

19. **WHO 2015a** World Health Organization. World report on Ageing and Health. 2015

20. **WHO 2015b** World Health Organization. Multisectoral action for a life course approach to healthy ageing: draft global strategy and plan of action on ageing and health. 2015

21. **WHO 2016** World Health Organization. Multisectoral action for a life course approach to healthy ageing: draft global strategy and plan of action on ageing and health. 2016

22. **WHO 2017b** World Health Organization. Global strategy and action plan on ageing and health. Geneva: World Health Organization. 2017. Licence: CC BY-NC-SA 3.0 IGO

23. **WHO 2017a** World Health Organization. Integrated care for older people Guidelines on community-level interventions to manage declines in intrinsic capacity. Geneva: World Health Organization. 2017. Licence: CC BY-NC-SA 3.0 IGO

24. **WHO 2017c** World Health Organization. WHO Clinical Consortium on Healthy Ageing. Report of consortium meeting 1–2 December 2016 in Geneva, Switzerland. Geneva: World Health Organization. 2017 (WHO/FWC/ALC/17.2). Licence: CC BY-NC-SA 3.0 IGO

25. **WHO 2019a** Cylus J, Figueras J, Normand C. Will population ageing spell the end of the welfare state? A review of evidence and policy options. World Health Organization. 2019

26. **WHO 2019b** Williams G, Cylus J, Roubal T, Ong P, Barber S. Sustainable health financing with an ageing population: Will population ageing lead to uncontrolled health expenditure growth? The economics of healthy and active ageing series. 2019

27. **WHO 2019c** World Health Organization. Regional Office for Europe. Reducing Inequities in Health Across the Life-course: Later Life and Healthy Ageing. Later life and healthy ageing. Copenhagen: WHO Regional Office for Europe. 2019. Licence: CC BY-NC-SA 3.0 IGO

28. **WHO 2020** World Health Organization. Decade of healthy ageing: baseline report. Geneva: World Health Organization. 2020. Licence: CC BY-NC-SA 3.0 IGO

29. **WHO 2021a** World Health Organization. Social isolation and loneliness among older people: advocacy brief. Geneva: World Health Organization. 2021. Licence: CC BY-NC-SA 3.0 IGO

30. **WHO 2021b** Greer SL, Linch J, Reeves A et al. Ageing and health: the politics of better policies. European observatory of health system and policies. Cambridge University Press. 2021. DOI 10.1017/9781108973236

31. **WHO 2022a** World Health Organization. Tackling abuse of older people: five priorities for the United Nations Decade of Healthy Ageing (2021–2030). Geneva: World Health Organization. 2022. Licence: CC BY-NC-SA 3.0 IGO

32. **WHO 2022b** Greer SL, Lynch JF, Reeves A, Raj M, Gingrich J, Falkenbach M, Cylus J, Bambra C. The Politics of Healthy Ageing: Myths and Realities. The Economics of Healthy and Active Ageing series. 2022

33. **WHO 2023** World Health Organization. National programmes for age-friendly cities and communities: a guide. Geneva: World Health Organization. 2023. Licence: CC BY-NC-SA 3.0 IGO
